# Supplementary material for: A differential requirement for ciliary transition zone proteins in human and mouse neural progenitor fate specification
Source: Nat Commun. 2025 Apr 5;16:3258. doi: 10.1038/s41467-025-58554-3 (PMC11972330; doi:10.1038/s41467-025-58554-3)
Supplement: Supplementary file 1 — Supplementary Information [file 41467_2025_58554_MOESM1_ESM.pdf]

SUPPLEMENTARY MATERIAL

A differential requirement for ciliary transition zone proteins in human and mouse neural progenitor fate specification

Antonia Wiegering[1,2], Isabelle Anselme[1,2], Ludovica Brunetti[1,2], Laura Metayer-Derout[1,2], Damelys Calderon[3], Sophie Thomas[3], Stéphane Nedelec[4,5], Alexis Eschstruth[1,2], Valentina Serpieri[6], Martin Catala[1,2], Christophe Antoniewski[2], Sylvie Schneider-Maunoury[1,2#], Aline Stedman[1,2#]

[1]Sorbonne Université, CNRS, Inserm, Development, Adaptation and Aging, Dev2A, F-75005 Paris, France
[2]Sorbonne Université, CNRS, Inserm, Institut de Biologie Paris Seine, Paris, France.
[3]INSERM UMR 1163, Institut Imagine, Université Paris Cité, Paris, France.
[4]Sorbonne Université, Inserm, Institut du Fer à Moulin, UMR-S 1270, Paris, France.
[5]Université Paris Cité, CNRS, Inserm U1340, Institut Jacques Monod, Paris, France.
[6]Department of Molecular Medicine, University of Pavia, Pavia, Italy.

[#]These authors jointly supervised this work

Corresponding authors:
antonia.wiegering@sorbonne-universite.fr
aline.stedman@sorbonne-universite.fr
sylvie.schneider-maunoury@sorbonne-universite.fr

a

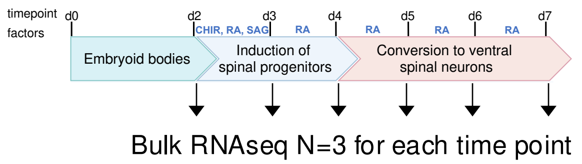

b

ENSEMBL ID	SYMBOL	GENENAME	log2(FC)	StdErr	P-value	P-adj
ENSMUSG00000031734	Irx3	Iroquois related homeobox 3	2,55320738	0,19097095	9,10E-41	1,30E-37
ENSMUSG00000045608	Dbx2	developing brain homeobox 2	2,91831216	0,23764821	1,16E-34	1,38E-31
ENSMUSG00000026468	Lhx4	LIM homeobox protein 4	-2,9380576	0,24563724	5,69E-33	6,37E-30
ENSMUSG00000031737	Irx5	Iroquois homeobox 5	2,50242649	0,22365503	4,63E-29	4,40E-26
ENSMUSG00000028736	Pax7	paired box 7	2,82332962	0,26018955	1,97E-27	1,70E-24
ENSMUSG00000025407	Gli1	GLI-Kruppel family member GLI1	-1,9410742	0,21344431	9,54E-20	4,91E-17
ENSMUSG00000028681	Ptch2	patched 2	-1,7762704	0,2057826	6,04E-18	2,30E-15
ENSMUSG00000033282	Rpgrip1l	Rpgrip1-like	-1,6304203	0,19973818	3,27E-16	1,07E-13
ENSMUSG00000042258	Isl1	ISL1 transcription factor, LIM/homeodomain	-2,0816022	0,25860359	8,32E-16	2,55E-13
ENSMUSG00000004231	Pax2	paired box 2	2,00227118	0,24969976	1,07E-15	3,23E-13
ENSMUSG00000035187	Nkx6-1	NK6 homeobox 1	-1,9061719	0,23892466	1,49E-15	4,35E-13
ENSMUSG00000026934	Lhx3	LIM homeobox protein 3	-1,9997871	0,26092015	1,80E-14	4,82E-12
ENSMUSG00000064325	Hhip	Hedgehog-interacting protein	-1,5537733	0,23469718	3,58E-11	6,56E-09
ENSMUSG00000021466	Ptch1	patched 1	-1,2540242	0,21122335	2,90E-09	4,18E-07
ENSMUSG00000027168	Pax6	paired box 6	1,27061788	0,26745032	2,03E-06	0,00015112
ENSMUSG00000001566	Mnx1	motor neuron and pancreas homeobox 1	-1,0246289	0,22843394	7,28E-06	0,00047731
ENSMUSG00000039830	Olig2	oligodendrocyte transcription factor 2	-0,9222057	0,25121862	0,00024167	0,01061787
ENSMUSG00000030507	Foxa2	developing brain homeobox 1	0,78473085	0,22126663	0,00039033	0,0158666

c **d**

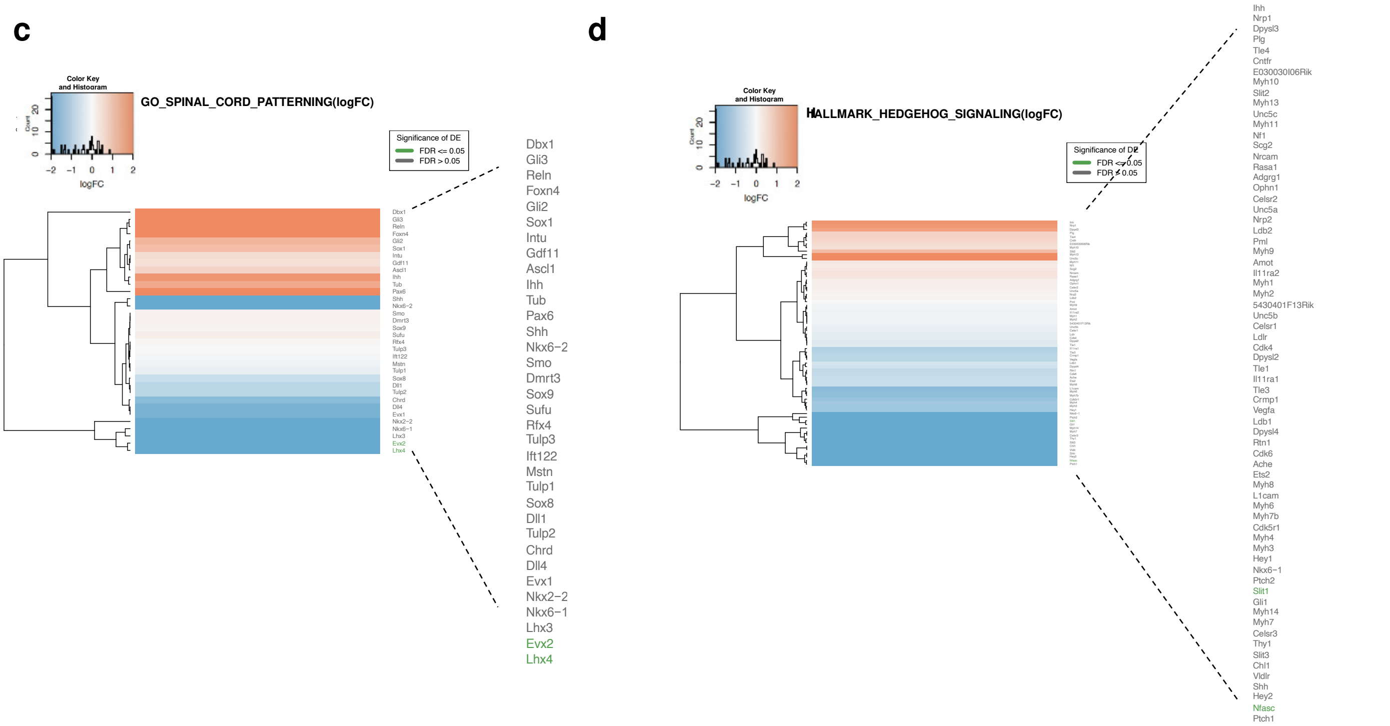

e **f** **g** **h**

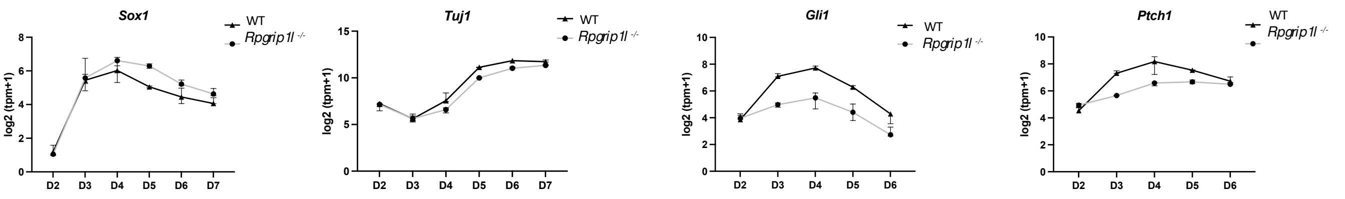

Supplementary Figure 1: Longitudinal transcriptomic analysis of WT and *Rpgrip1l*$^{-/-}$ spinal organoids. **a** Diagram depicting the time points of spinal differentiation chosen for bulk RNAseq analysis. **b** Table listing selected top-differentially expressed genes between WT and *Rpgrip1l*$^{-/-}$ day 4 spinal organoids obtained by DEseq2 analysis. **c**, **d** Ensemble of Gene Set Enrichment Analyses (*EGSEA*) performed on differentially expressed genes between WT and *Rpgrip1l*$^{-/-}$ organoids on day 4. Heatmaps ranked 1st for c5 GO Gene Sets (c), and h Hallmark Signatures (d) are shown. **e-h** Temporal analysis of *Sox1* (NPs), *Tuj1* (early neurons), *Gli1* and *Patched1* (SHH targets) in the course of the differentiation of WT and *Rpgrip1l*$^{-/-}$ organoids. Log2(tpm+1) data from bulk RNASeq analysis are displayed as mean ± SEM (N=3 independent experiments for each genotype).

a

Guide	Sequence + PAM	Crispr Score	Off Target
RPGRIP1L_exon3	ACGGACAATGAAGTCTCGCC AGG	92	0-0-0-8-38

b

Cell line	Origin	# clone	Genotype	Method	Mutation
PCli033-A	PHENOCELL (PCI)	42	+/+	InDel	-
PCli033-A	PHENOCELL (PCI)	46	+/+	InDel	-
PCli033-A	PHENOCELL (PCI)	20	-/-	InDel	c.114_118del; p.Arg39Glyfs*8<br>c.116del; p.Arg39Profs*44
PCli033-A	PHENOCELL (PCI)	44	-/-	InDel	c.115dup; p.Arg39Profs*10<br>c.115dup; p.Arg39Profs*10
UCSFi001-A	Conklin Lab Gladstone/UCS	19	+/+	InDel	-
UCSFi001-A	Conklin Lab Gladstone/UCS	62	+/+	InDel	-
UCSFi001-A	Conklin Lab Gladstone/UCS	44	-/-	InDel	c.114_115insG; p.Arg39Alafs*10<br>c.116_117delinsT;p.Arg39Leufs*44
UCSFi001-A	Conklin Lab Gladstone/UCS	65	-/-	InDel	c.114_115dup; p.Arg39Leufs*45<br>c.114_115dup; p.Arg39Leufs*45

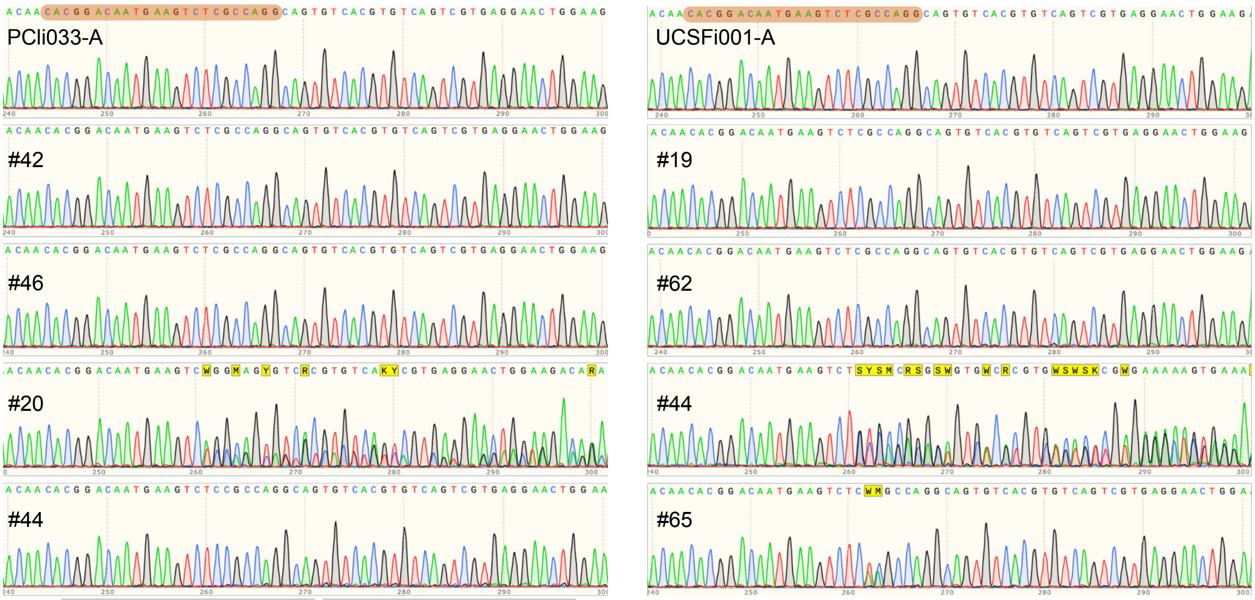

c

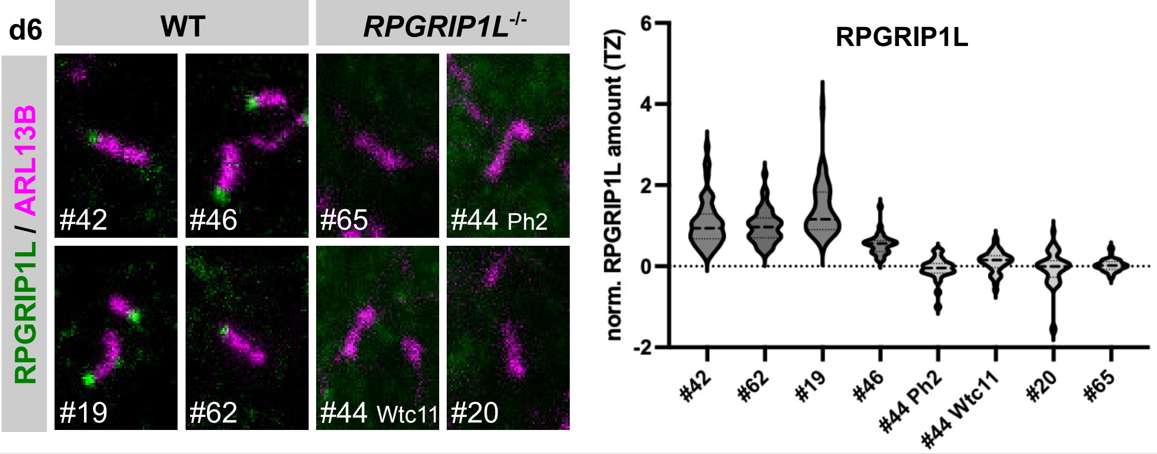

d

on/off target	CFD Score
ontarget_mm0_exon_RPGRIP1L_chr16_53730171_F	1.00
ontarget_mm0_exon_RPGRIP1L_chr16_53570171_R	1.00
mm4_exon_NPRL2/CYB561D2/XXcos-LUCA11.5_chr3_50385586_F	0.18
mm4_exon_NPRL2/CYB561D2/XXcos-LUCA11.5_chr3_50385586_R	0.18

e

```
           1                                                                                                                     118
 RPGRIP1L                                  ACGGACAATGAAGTCTCGCCAGG
GRCh38.p14 CGTTCATCAACATGCTGCAGCTGCTGGGGGTGGCGGCCAATGAGGTCTCGCACGGTAGTGCCAGGGCTCAGGCTGCAGTATAGCTGGAACACATCCCGGAGACTGGCCCTCTTGTGCC
    Ph2_42 CGTTCATCAACATGCTGCAGCTGCTGGGGGTGGCGGCCAATGAGGTCTCGCACGGTAGTGCCAGGGCTCAGGCTGCAGTATAGCTGGAACACATCCCGGAGACTGGCCCTCTTGTGCC
    Ph2_46 CGTTCATCAACATGCTGCAGCTGCTGGGGGTGGCGGCCAATGAGGTCTCGCACGGTAGTGCCAGGGCTCAGGCTGCAGTATAGCTGGAACACATCCCGGAGACTGGCCCTCTTGTGCC
    Ph2_20 CGTTCATCAACATGCTGCAGCTGCTGGGGGTGGCGGCCAATGAGGTCTCGCACGGTAGTGCCAGGGCTCAGGCTGCAGTATAGCTGGAACACATCCCGGAGACTGGCCCTCTTGTGCC
    Ph2_44 CGTTCATCAACATGCTGCAGCTGCTGGGGGTGGCGGCCAATGAGGTCTCGCACGGTAGTGCCAGGGCTCAGGCTGCAGTATAGCTGGAACACATCCCGGAGACTGGCCCTCTTGTGCC
  Wtc11_19 CGTTCATCAACATGCTGCAGCTGCTGGGGGTGGCGGCCAATGAGGTCTCGCACGGTAGTGCCAGGGCTCAGGCTGCAGTATAGCTGGAACACATCCCGGAGACTGGCCCTCTTGTGCC
  Wtc11_62 CGTTCATCAACATGCTGCAGCTGCTGGGGGTGGCGGCCAATGAGGTCTCGCACGGTAGTGCCAGGGCTCAGGCTGCAGTATAGCTGGAACACATCCCGGAGACTGGCCCTCTTGTGCC
  Wtc11_44 CGTTCATCAACATGCTGCAGCTGCTGGGGGTGGCGGCCAATGAGGTCTCGCACGGTAGTGCCAGGGCTCAGGCTGCAGTATAGCTGGAACACATCCCGGAGACTGGCCCTCTTGTGCC
  Wtc11_65 CGTTCATCAACATGCTGCAGCTGCTGGGGGTGGCGGCCAATGAGGTCTCGCACGGTAGTGCCAGGGCTCAGGCTGCAGTATAGCTGGAACACATCCCGGAGACTGGCCCTCTTGTGCC
 Consensus CGTTCATCAACATGCTGCAGCTGCTGGGGGTGGCGGCCAATGAGGTCTCGCACGGTAGTGCCAGGGCTCAGGCTGCAGTATAGCTGGAACACATCCCGGAGACTGGCCCTCTTGTGCC
```

Supplementary Figure 2: Generation of RPGRIP1L-deficient hiPSC lines. **a** Sequence of the CRISPR-guide that was used for the generation of mutations in exon 3 of *RPGRIP1L*. The Crispr Score corresponds to the CFD specificity score based on the CFD off-target model. The Off Target field indicates the number of off-targets for each number of mismatches (8 off-targets with 3 mismatches and 38 off-targets with 4 mismatches). None of these mismatches are in the 12 bp adjacent to the PAM. **b** List of hiPSC clones that were used in this project. The original hiPSC line (PCli033-A or UCSFi001-A), clone number, genotype and specific mutation are indicated for each clone. Sequencing chromatograms are presented for each clone and original cell line. The CRISPR-guide sequence is indicated in orange. **c** Immunofluorescence of RPGRIP1L in WT and RPGRIP1L-deficient cilia in spinal organoids at day 6. Cilia are labeled by ARL13B and RPGRIP1L. Data are shown as median with quartiles. N=3 experimental replicates per clone. **d** Identified off-targets and corresponding CFD scores for the *RPGRIP1L_exon3* guide. **e** Off-target analyses via sequencing of the *NPRL2* locus. Sequence of the CRISPR-guide is indicated above. Bases highlighted in yellow and green indicate mismatches between the guide sequence and the off-target sequence. No mutation in *NPRL2* was detectable around the off-target site in WT and KO hiPSC clones.

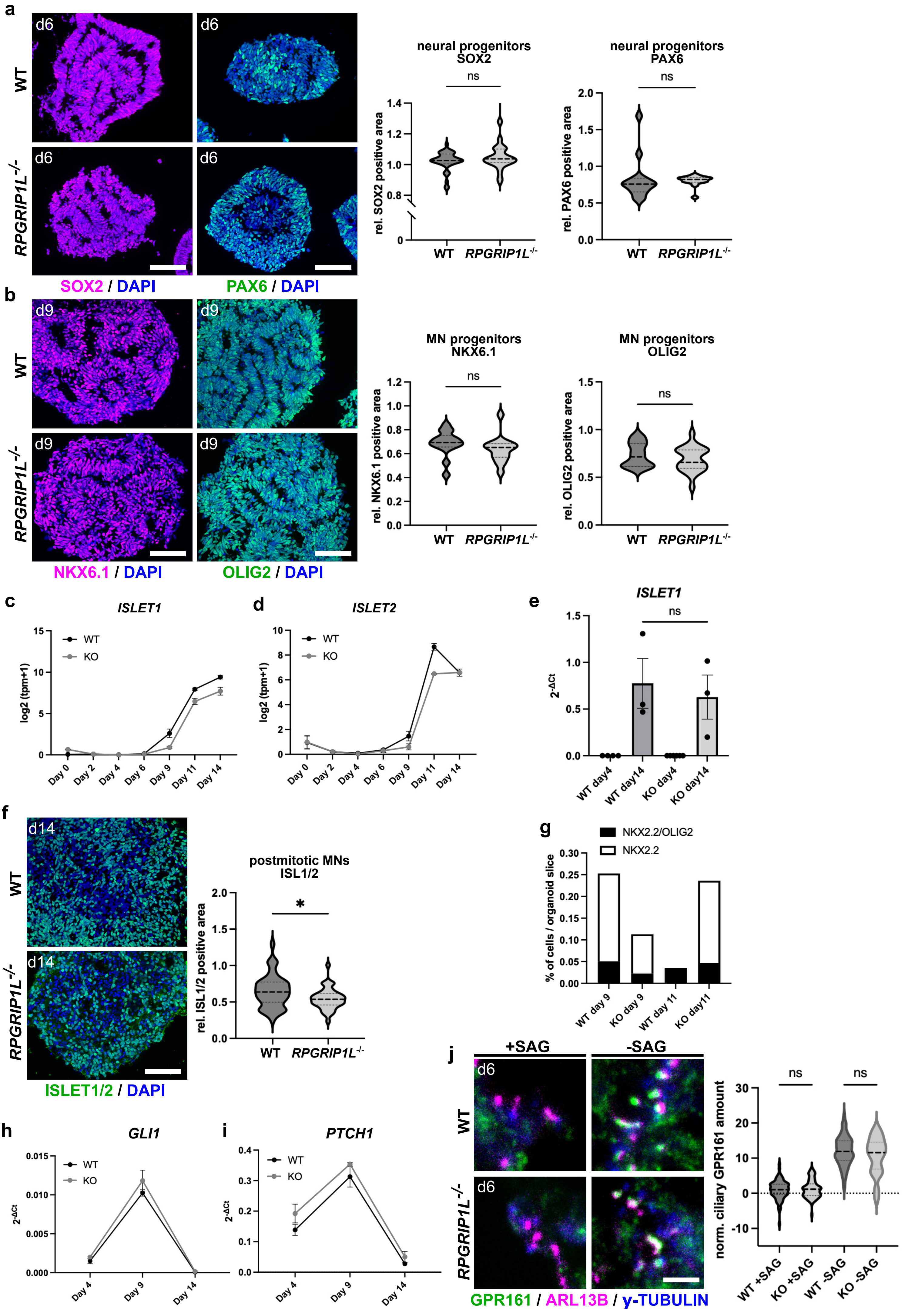

a
d6
WT
RPGRIP1L-/-
SOX2 / DAPI
PAX6 / DAPI
neural progenitors SOX2
ns
rel. SOX2 positive area
WT
RPGRIP1L-/-
neural progenitors PAX6
rel. PAX6 positive area
b
d9
NKX6.1 / DAPI
OLIG2 / DAPI
MN progenitors NKX6.1
rel. NKX6.1 positive area
MN progenitors OLIG2
rel. OLIG2 positive area
c
ISLET1
WT
KO
log2 (tpm+1)
Day 0
Day 2
Day 4
Day 6
Day 9
Day 11
Day 14
d
ISLET2
e
ISLET1
2-ΔCt
WT day4
WT day14
KO day4
KO day14
f
d14
postmitotic MNs ISL1/2
*
rel. ISL1/2 positive area
ISLET1/2 / DAPI
g
NKX2.2/OLIG2
NKX2.2
% of cells / organoid slice
WT day 9
KO day 9
WT day 11
KO day11
h
GLI1
i
PTCH1
j
+SAG
-SAG
GPR161 / ARL13B / γ-TUBULIN
norm. ciliary GPR161 amount
WT +SAG
KO +SAG
WT -SAG
KO -SAG

Supplementary Figure 3: Phenotyping of WT and RPGRIP1L-deficient organoids. a, b IF analysis of day 6 neural progenitors and day 9 pMNs in WT and *RPGRIP1L*$^{-/-}$ organoids. Quantifications are shown as median with quartiles. Statistics: unpaired t tests with Welch's correction. **c**, **d** Log2(tpm+1)-graphs show temporal expression of *ISLET1* and *ISLET2* in WT and *RPGRIP1L*$^{-/-}$ organoids. Data are shown as mean ± SEM. **e** qPCR analysis of *ISLET1* in WT and *RPGRIP1L*$^{-/-}$ organoids at days 4 and 14. Statistics: Kruskal-Wallis with Dunn's multiple comparison test. **f** IF analysis of MNs in WT and *RPGRIP1L*$^{-/-}$ organoids at day 14. Quantifications are shown as median with quartiles. Statistics: unpaired t tests with Welch's correction (p=0.0125). **g** Quantification of the percentage of NKX2.2-positive and NKX2.2/OLIG2 double-positive cells per organoid section for WT and RPGRIP1L-deficient organoids at days 9 and 11. Statistics: Chi-square tests. **h**, **i** *GLI1* and *PTCH1* qPCR analyses in WT and *RPGRIP1L*$^{-/-}$ organoids. Data are shown as mean ± SEM. Statistics: Mann-Whitney tests at each time point. **j** IF analysis of ciliary GPR161 amount in WT and *RPGRIP1L*$^{-/-}$ day 6 organoids treated either with SAG or DMSO. Quantifications are shown as median with quartiles. Statistics: Kruskal-Wallis with Dunn's multiple comparison test. **a-j** N: number of independent experiments, n: number of different WT or KO clones per experiment. For a, b, f, g and j, 2 WT and 2 KO clones from each iPSC line) (n=4 for each). For c and d, 2 WT and 1 KO clones from each line (n=4 WT and n=2 KO). For e, h, and i, 2 WT and 2 KO clones from each line (n=4 for each) at day 4; 2 WT and 2 KO PCli033-A clones and 1 WT and 1 KO UCSFi001-A clones (n=3 for each) at day 9 and day 14. N=3 for a, b, f; N=2 for g; N=1 for c, d, e, h, i, j. Scale bars: 100 µm in a, b, f; 2.5 µm in j.

a

Guide	Sequence + PAM	Crispr Score	Off Target
RPGRIP1L_exon3	ACGGACAATGAAGTCTCGCC AGG	92	0-0-0-8-38
RPGRIP1L_exon27	TATGCTTAGTTAGACGTGAA AGG	96	0-0-1-5-61

b

Cell line	Origin	# clone	Genotype	Method	Mutation
PCli033-A	PHENOCELL (PCI)	F6	+/-	KO	+ / *Δex3-ex27*
PCli033-A	PHENOCELL (PCI)	C5	-/-	KO	*Δex3-x27 / Δex3-ex27*

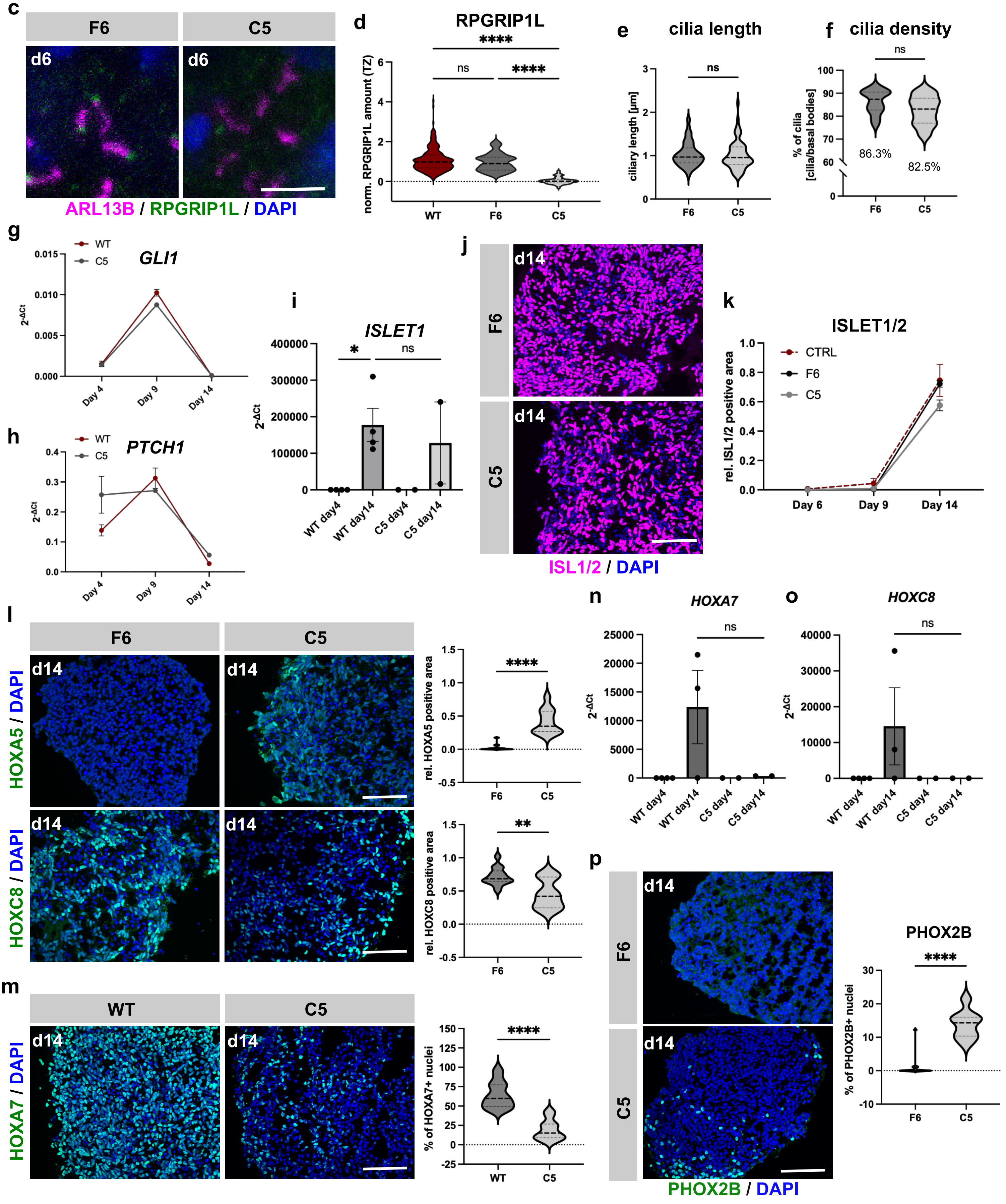

Supplementary Figure 4: Phenotyping spinal organoids from full-deletion RPGRIP1L hiPSCs. **a** Sequences of the CRISPR-guides in RPGRIP1L exons 3 and 27. The CRISPR Scores are CFD specificity scores based on the CFD off-target model. Off-targets for each number of mismatches are indicated, one of which is in the 12 bp adjacent to the PAM. **b** The hiPSC line of origin, clone number, genotype and mutation are indicated for each clone. **c** Immunofluorescence for RPGRIP1L in *RPGRIP1L*$^{+/-}$ and *RPGRIP1L*$^{-/-}$ day 6 organoids. **d** Quantification of ciliary RPGRIP1L amounts in WT, *RPGRIP1L*$^{+/-}$ and *RPGRIP1L*$^{-/-}$ spinal organoids at day 6. WT is described in Supplementary Fig. 2. Data are shown as median with quartiles. Statistics: Kruskal-Wallis with Dunn's multiple comparison test ($p < 0.0001$). **e**, **f** Quantifications of ciliary length and density in *RPGRIP1L*$^{+/-}$ and *RPGRIP1L*$^{-/-}$ day 6 organoids. Data are shown as median with quartiles. Statistics: Mann-Whitney test. **g**, **h** *GLI1* and *PTCH1* qPCR analyses in WT and *RPGRIP1L*$^{-/-}$ organoids. Statistics: Mann-Whitney tests at each time point. **i** qPCR analysis of *ISLET1* in WT and *RPGRIP1L*$^{-/-}$ organoids at day 4 and day 14. Statistics: Kruskal-Wallis and Dunn's multiple comparison test ($p=0.0268$). **j** Immunofluorescence analysis of MNs (ISLET1/2) in *RPGRIP1L*$^{+/-}$ and *RPGRIP1L*$^{-/-}$ organoids. **k** Quantifications of relative ISLET1/2 positive area in WT, *RPGRIP1L*$^{+/-}$ and *RPGRIP1L*$^{-/-}$ organoids. Data are shown as mean ± SEM. Statistics: Kruskal Wallis with Dunn's multiple comparison test. **l, m** Immunofluorescence analysis of HOXA5, HOXC8 and HOXA7 in WT (m), *RPGRIP1L*$^{+/-}$ (l) and *RPGRIP1L*$^{-/-}$ (l, m) day 14 organoids. Quantifications are shown as median with quartiles. Statistics: unpaired t tests with Welch's correction (l: **$p=0.0089$; ****$p < 0.0001$) (m: $p < 0.0001$). **n**, **o** qPCR analyses of *HOXA7* and *HOXC8* in WT and *RPGRIP1L*$^{-/-}$ organoids at days 4 and 14. Statistics: Kruskal-Wallis and Dunn's multiple comparison test. **p** Immunofluorescence analysis of PHOX2B in *RPGRIP1L*$^{+/-}$ and *RPGRIP1L*$^{-/-}$ day 14 organoids. Quantifications are shown as median with quartiles. Statistics: unpaired t tests with Welch's correction ($p < 0.0001$). **d-p** N: number of independent experiments; n: number of different clones analyzed per experiment. For d, k and m, 2 WT clones from each line (n=4). For WT, N=4 in d and N=1 in k and m. N=3 for F6 and C5. For g, h, i, n and o, 2 WT clones from each line at day 4 (n=4) and 2 PCli033-A and 1 UCSFi001-A WT clones (n=3) at day 9 and day 14. C5: N=2. In e, f, k, l and p, N=3 for F6 and C5. Scale bars: 2.5 µm in c and 250 µm in j, l, m, p.

a

Guide	Sequence + PAM	Crispr Score	Off Target
TMEM67_exon1	GGTTAGAGACCGGTTAACCC TGG	97	0-0-0-1-21
TMEM67_exon27	ATCTCGGCGCTCACTAGCAG TGG	94	0-0-0-0-29

b

Cell line	Origin	# clone	Genotype	Method	Mutation
PCli033-A	PHENOCELL (PCI)	#1	+/-	KO	+ / *Δex1-ex27*
PCli033-A	PHENOCELL (PCI)	#14	-/-	KO	*Δex1-x27 / Δex1-ex27*

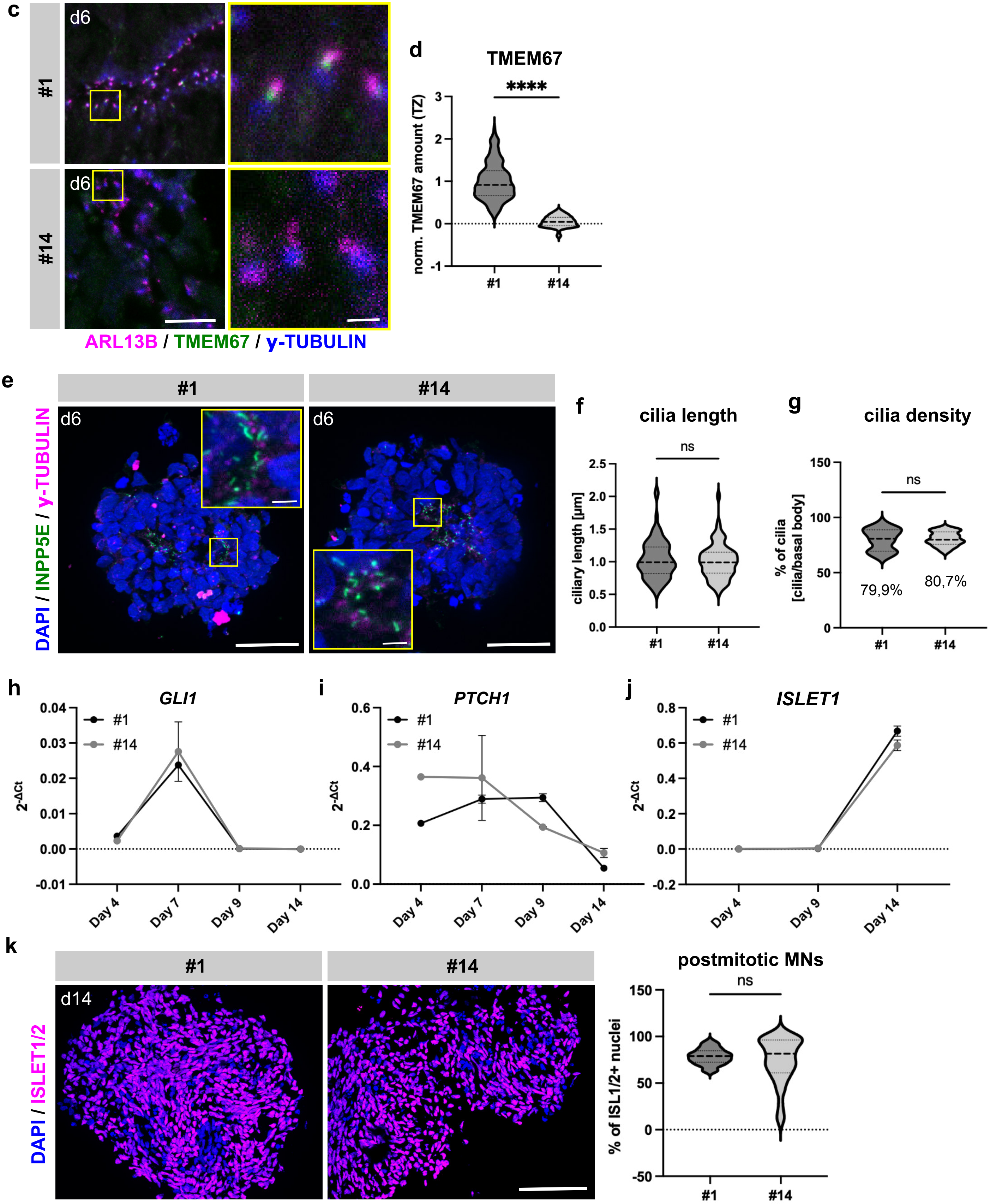

Supplementary Figure 5: Spinal MN differentiation of TMEM67-deficient hiPSC lines. a Sequences of the CRISPR-guides in exons 1 and 27 used for the generation of a full-deletion hiPSC line. The CRISPR Score is the CFD specificity score based on the CFD off-target model. The Off Target field indicates the number of off-targets for each number of mismatches, none of which are in the 12 bp adjacent to the PAM. **b** List of hiPSC lines used. The iPSC line of origin, clone number, genotype and specific mutation are indicated for each clone. **c** Immunofluorescence for TMEM67 in *TMEM67*$^{+/-}$ and *TMEM67*$^{-/-}$ day 6 organoids. Cilia are labeled by ARL13B and basal bodies by γ-TUBULIN. TMEM67 is labeled in green. **d** Quantification of the ciliary TMEM67 amount in *TMEM67*$^{+/-}$ and *TMEM67*$^{-/-}$ spinal organoids at day 6. Data are shown as median with quartiles. Asterisks denote statistical significance according to unpaired t tests with Welch's correction ($P < 0.0001$). **e** Immunofluorescence staining of primary cilia in *TMEM67*$^{+/-}$ and *TMEM67*$^{-/-}$ spinal organoids. Cilia are labeled by INPP5E and basal bodies by γ-TUBULIN. **f**, **g** Quantification of cilia length and density in *TMEM67*$^{+/-}$ and *TMEM67*$^{-/-}$ spinal organoids at day 6. Statistics: unpaired t tests with Welch's correction. **h**, **i**, **j** qPCR analyses of *GLI1*, *PTCH1* and *ISLET1* gene expression profiles in *TMEM67*$^{+/-}$ and *TMEM67*$^{-/-}$ spinal organoids over time. Data are presented as mean ± SEM. Statistics: Mann-Whitney tests. **k** Immunofluorescence analysis of MNs in *TMEM67*$^{+/-}$ and *TMEM67*$^{-/-}$ day 14 organoids. Quantifications are shown as median with quartiles. Statistics: unpaired t test with Welch's correction. **c-k** N: number of independent experimental replicates. N=3 for #1 and #14 in c, f, g, h-k. Scale bars: 10 µm in c, 50 µm in e and 150 µm in k. For magnified images (yellow squares): 1 µm in c, 2 µm in e.

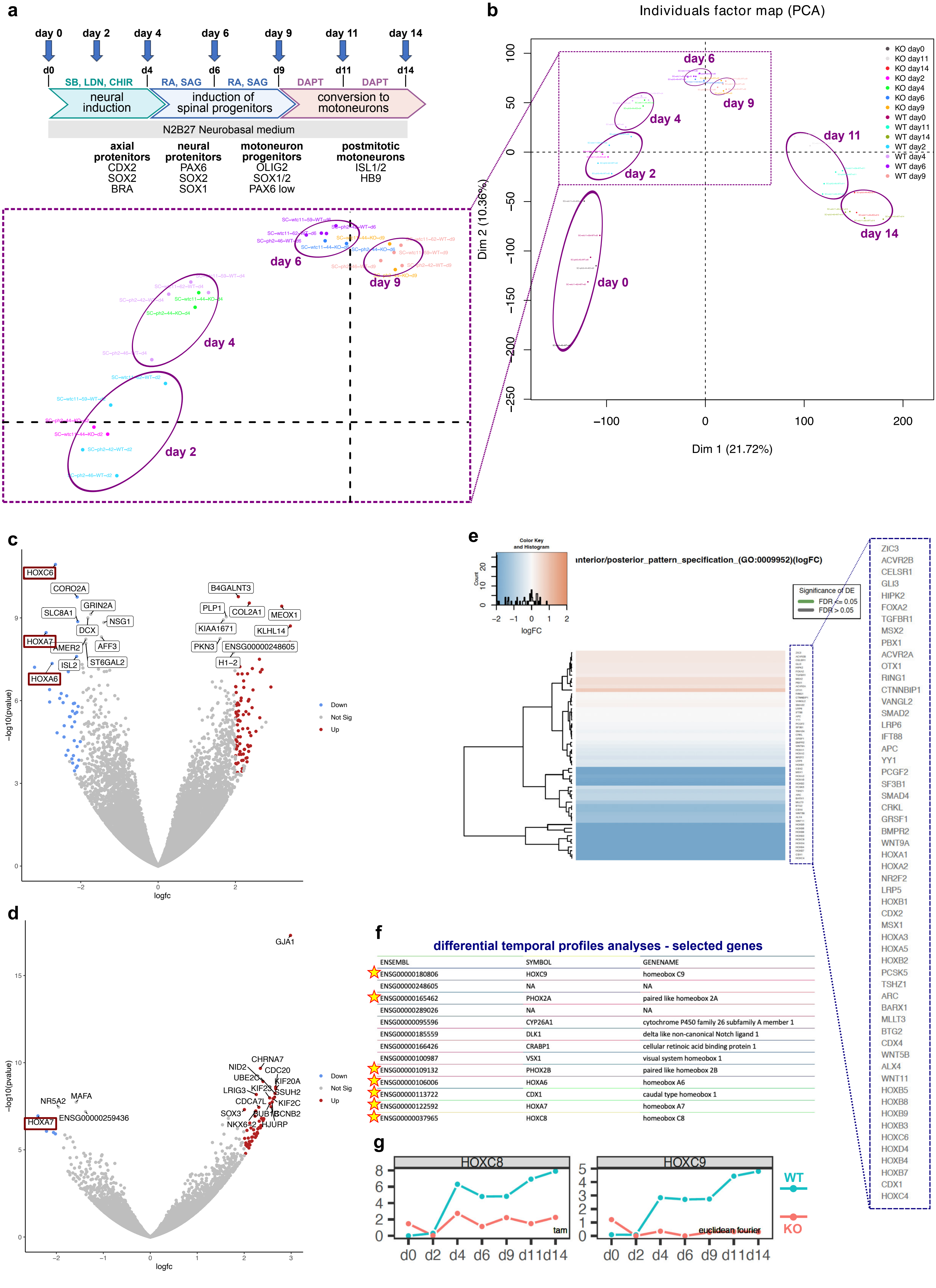
a
day 0
day 2
day 4
day 6
day 9
day 11
day 14
d0
d4
d6
d9
d11
d14
SB, LDN, CHIR
RA, SAG
RA, SAG
DAPT
DAPT
neural induction
induction of spinal progenitors
conversion to motoneurons
N2B27 Neurobasal medium
axial protenitors
CDX2
SOX2
BRA
neural protenitors
PAX6
SOX2
SOX1
motoneuron progenitors
OLIG2
SOX1/2
PAX6 low
postmitotic motoneurons
ISL1/2
HB9
b
Individuals factor map (PCA)
Dim 1 (21.72%)
Dim 2 (10.36%)
KO day0
KO day11
KO day14
KO day2
KO day4
KO day6
KO day9
WT day0
WT day11
WT day14
WT day2
WT day4
WT day6
WT day9
c
HOXC6
CORO2A
GRIN2A
SLC8A1
NSG1
DCX
HOXA7
AMER2
AFF3
ISL2
ST6GAL2
HOXA6
B4GALNT3
PLP1
COL2A1
MEOX1
KIAA1671
KLHL14
PKN3
ENSG00000248605
H1−2
Down
Not Sig
Up
logfc
−log10(pvalue)
d
GJA1
CHRNA7
NID2
CDC20
UBE2C
KIF20A
LRIG3
KIF23
SSUH2
CDCA7L
KIF2C
SOX3
BUB1B
CCNB2
NKX6−2
HJURP
NR5A2
MAFA
HOXA7
ENSG00000259436
e
Color Key and Histogram
Count
logFC
anterior/posterior_pattern_specification_(GO:0009952)(logFC)
Significance of DE
FDR <= 0.05
FDR > 0.05
ZIC3
ACVR2B
CELSR1
GLI3
HIPK2
FOXA2
TGFBR1
MSX2
PBX1
ACVR2A
OTX1
RING1
CTNNBIP1
VANGL2
SMAD2
LRP6
IFT88
APC
YY1
PCGF2
SF3B1
SMAD4
CRKL
GRSF1
BMPR2
WNT9A
HOXA1
HOXA2
NR2F2
LRP5
HOXB1
CDX2
MSX1
HOXA3
HOXA5
HOXB2
PCSK5
TSHZ1
ARC
BARX1
MLLT3
BTG2
CDX4
WNT5B
ALX4
WNT11
HOXB5
HOXB8
HOXB9
HOXB3
HOXC6
HOXD4
HOXB4
HOXB7
CDX1
HOXC4
f
differential temporal profiles analyses - selected genes
ENSEMBL | SYMBOL | GENENAME
ENSG00000180806 | HOXC9 | homeobox C9
ENSG00000248605 | NA | NA
ENSG00000165462 | PHOX2A | paired like homeobox 2A
ENSG00000289026 | NA | NA
ENSG00000095596 | CYP26A1 | cytochrome P450 family 26 subfamily A member 1
ENSG00000185559 | DLK1 | delta like non-canonical Notch ligand 1
ENSG00000166426 | CRABP1 | cellular retinoic acid binding protein 1
ENSG00000100987 | VSX1 | visual system homeobox 1
ENSG00000109132 | PHOX2B | paired like homeobox 2B
ENSG00000106006 | HOXA6 | homeobox A6
ENSG00000113722 | CDX1 | caudal type homeobox 1
ENSG00000122592 | HOXA7 | homeobox A7
ENSG00000037965 | HOXC8 | homeobox C8
g
HOXC8
HOXC9
tam
euclidean fourier
d0 d2 d4 d6 d9 d11 d14
WT
KO

Supplementary Figure 6: Bulk RNAseq analysis indicates altered antero-posterior patterning in RPGRIP1L-deficient spinal organoids. a Schematic summary of the human spinal 3D differentiation approach. Samples for bulk RNAseq analyses were collected at indicated time points. The clones were generated from two independent iPCS lines, PCIi033-1 and UCSFi001-A. 2 WT clones and 1 KO clone from each line were analyzed (WT: n=4, KO: n=2). **b** PCA analyses performed on WT and KO samples show the variation of data over time. The zoom-in on the left shows that WT and KO samples cluster closely together at single time points. **c**, **d** Volcano plots of RNAseq data on day 11 (c) and day 14 (d). The threshold for significance was set to 0.01 and the LogFC threshold to 2. **e** EGSEA at day 2 of spinal differentiation shows downregulated *anterior/posterior_pattern_specification* (GO:0009952) in RPGRIP1L-deficient organoids. Genes that correspond to this GO term are depicted on the right. **f** List of genes with different temporal expression profiles based on euclidean distance measurements between WT and *RPGRIP1L KO* organoids over the entire time course of differentiation. Asterisks indicate genes that are related to antero-posterior patterning. **g** Examples of genes with different temporal expression profiles. *HOXC8* was captured via time alignment measurement (tam) and *HOXC9* via euclidean measurement.

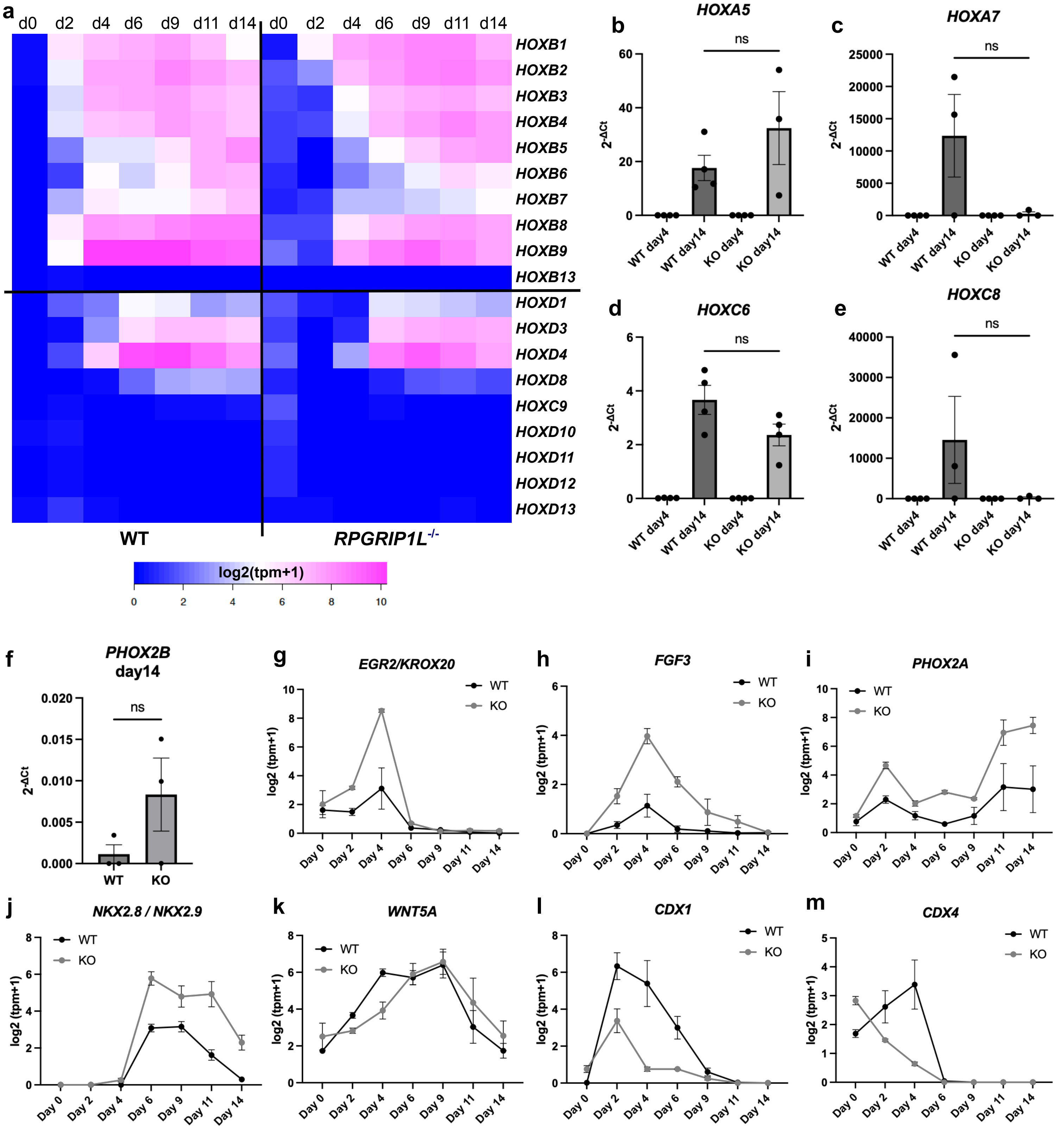

a
d0 d2 d4 d6 d9 d11 d14 d0 d2 d4 d6 d9 d11 d14
HOXB1
HOXB2
HOXB3
HOXB4
HOXB5
HOXB6
HOXB7
HOXB8
HOXB9
HOXB13
HOXD1
HOXD3
HOXD4
HOXD8
HOXC9
HOXD10
HOXD11
HOXD12
HOXD13
WT
RPGRIP1L-/-
log2(tpm+1)
b
HOXA5
c
HOXA7
d
HOXC6
e
HOXC8
ns
2-ΔCt
WT day4
WT day14
KO day4
KO day14
f
PHOX2B day14
g
EGR2/KROX20
h
FGF3
i
PHOX2A
j
NKX2.8 / NKX2.9
k
WNT5A
l
CDX1
m
CDX4
WT
KO
log2 (tpm+1)
Day 0
Day 2
Day 4
Day 6
Day 9
Day 11
Day 14

Supplementary Figure 7: Antero-posterior patterning defects in RPGRIP1L-deficient spinal organoids. a Heatmap showing *HOXB* and *HOXD* gene expressions in WT and RPGRIP1L-deficient spinal organoids over time. The graph was generated from log2(tpm+1) files of bulk RNASeq analysis. **b-e** qPCR analyses of *HOXA5*, *HOXA7*, *HOXC6* and *HOXC8* in WT and RPGRIP1L-deficient organoids at day 4 and day 14. Kruskal-Wallis and Dunn's multiple comparison tests were performed for statistical analyses. **f** qPCR analysis of *PHOX2B* in WT and RPGRIP1L-deficient organoids at day 14. Unpaired t test with Welch's correction was performed for statistical analyses. **g-m** Log2(tpm+1) graphs show expression profiles of EGR2/KROX20 (g) *FGF3* (h), *PHOX2A* (i), *NKX2.8/NKX2.9* (j), *WNT5A* (k), *CDX1* (l) and *CDX4* (m) over time. Data are shown as mean ± SEM. **a-m** N=1 number of experimental replicates per clone. n: number of different WT or KO clones analyzed per experiment. For a and g-m, 2 WT clones and 1 KO clone from each line (n=4 for WT and n=2 for KO). For b-e, 2 WT clones and 2 KO clones of each iPSC line at day 4 (n=4 for each genotype), and 2 WT and 2 KO clones in the PCli033-A background and 2 WT and 1 KO clone in the UCSFi001-A background (n=4 for WT and n=3 for KO) at day 14. For f, 2 WT and 2 KO clones in the PCli033-A background and 1 WT and 1 KO clone in the UCSFi001-A background (n=3 for each genotype).

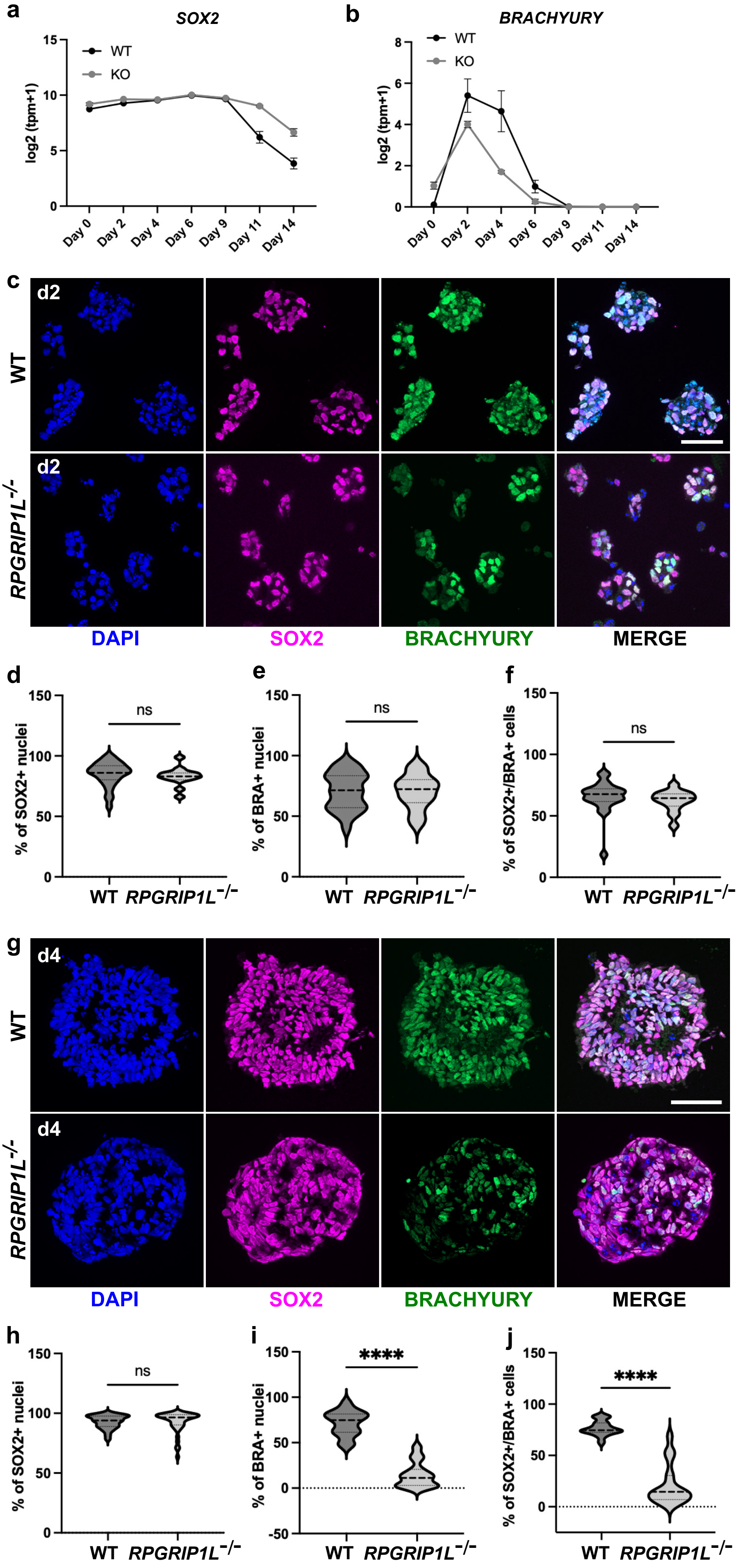
a
SOX2
WT
KO
log2 (tpm+1)
Day 0
Day 2
Day 4
Day 6
Day 9
Day 11
Day 14
b
BRACHYURY
WT
KO
log2 (tpm+1)
c
d2
WT
RPGRIP1L-/-
DAPI
SOX2
BRACHYURY
MERGE
d
% of SOX2+ nuclei
ns
WT RPGRIP1L-/-
e
% of BRA+ nuclei
ns
WT RPGRIP1L-/-
f
% of SOX2+/BRA+ cells
ns
WT RPGRIP1L-/-
g
d4
WT
RPGRIP1L-/-
DAPI
SOX2
BRACHYURY
MERGE
h
% of SOX2+ nuclei
ns
WT RPGRIP1L-/-
i
% of BRA+ nuclei
****
WT RPGRIP1L-/-
j
% of SOX2+/BRA+ cells
****
WT RPGRIP1L-/-

Supplementary Figure 8: hiPSCs adopt an NMP-like axial fate during early stages of ventral spinal differentiation. a, **b** Log2(tpm+1) graphs generated from bulk RNASeq analyses show the expression profiles of *SOX2* and *BRACHYURY* over time. Data are shown as mean ± SEM. **c**, **g** Immunofluorescence of SOX2 and BRACHYURY in WT and RPGRIP1L-deficient spinal organoids at day 2 and day 4. Scale bars: 150 µm. **d-f, h-j** Quantifications show the percentage of SOX2, BRACHYURY and SOX2/BRACHYURY positive nuclei per organoid at day 2 and day 4. Data are shown as median with quartiles. Statistics: unpaired t tests with Welch's correction ($p < 0.0001$). **a-j** n: number of different WT or KO clones analyzed per experiment. For a and b, 2 WT clones and 1 KO clone from each line (n=4 for WT and n=3 for KO). For d-f and h-j, 2 WT clones and 2 KO clones from each iPSC line (n=4 for each genotype). For d-f, number of independent experimental replicates N=1 and for h-i, number of independent experimental replicates N=3.

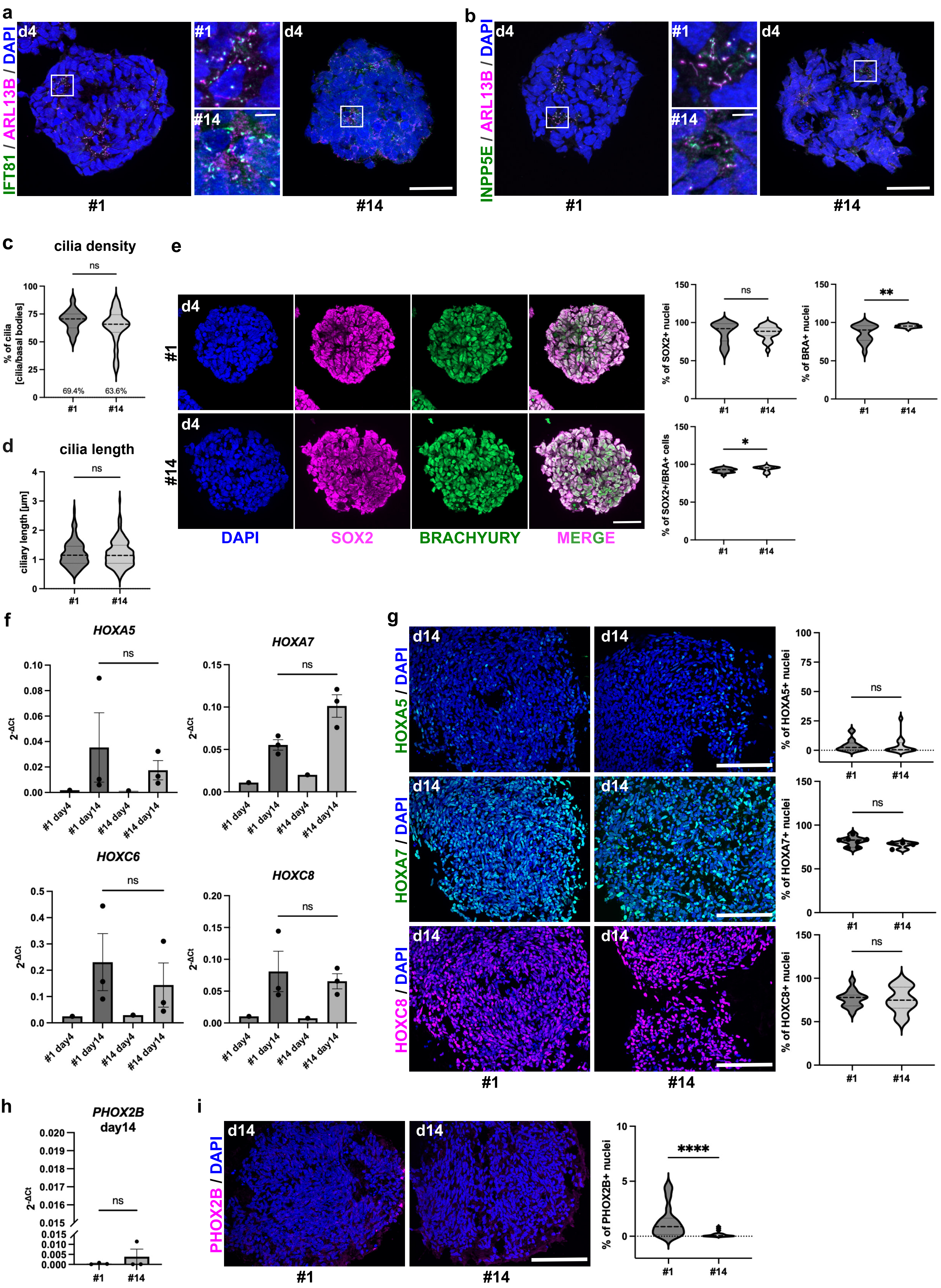
a
d4
IFT81 / ARL13B / DAPI
#1
#14
d4
#1
#14
b
d4
INPP5E / ARL13B / DAPI
#1
#14
d4
#1
#14
c
cilia density
ns
% of cilia [cilia/basal bodies]
69.4%
63.6%
#1
#14
d
cilia length
ns
ciliary length [µm]
#1
#14
e
d4
#1
d4
#14
DAPI
SOX2
BRACHYURY
MERGE
% of SOX2+ nuclei
ns
% of BRA+ nuclei
**
% of SOX2+/BRA+ cells
*
f
HOXA5
HOXA7
HOXC6
HOXC8
ns
2-ΔCt
#1 day4
#1 day14
#14 day4
#14 day14
g
d14
HOXA5 / DAPI
HOXA7 / DAPI
HOXC8 / DAPI
#1
#14
% of HOXA5+ nuclei
% of HOXA7+ nuclei
% of HOXC8+ nuclei
ns
h
PHOX2B
day14
ns
2-ΔCt
#1
#14
i
d14
PHOX2B / DAPI
#1
#14
% of PHOX2B+ nuclei
****

Supplementary Figure 9: TMEM67-deficient spinal organoids display unaltered axial progenitor specification and correct antero-posterior patterning. a, **b** Immunofluorescence of cilia in *TMEM67*$^{+/-}$ and *TMEM67*$^{-/-}$ day 4 organoids. Cilia are labeled by IFT81and ARL13B (a) or by INPP5E and ARL13B (b). **c**, **d** Quantification of cilia density and ciliary length in *TMEM67*$^{+/-}$ and *TMEM67*$^{-/-}$ day 4 organoids. Data are shown as median with quartiles. Statistics: unpaired t test with Welch's correction (c) and Mann-Whitney test (d). **e** Immunofluorescence of SOX2 and BRACHYURY in *TMEM67*$^{+/-}$ and *TMEM67*$^{-/-}$ spinal organoids at day 4. Data are shown as median with quartiles. Statistics: unpaired t tests with Welch's correction (*p=0.0424, **p=0.0057). **f** qPCR analyses of *HOXA5*, *HOXA7*, *HOXC6* and *HOXC8* in *TMEM67*$^{+/-}$ and *TMEM67*$^{-/-}$ organoids at day 4 and day 14. Statistics: Mann-Whitney tests. **g** Immunofluorescence of HOXA5, HOXA7 and HOXC8 in *TMEM67*$^{+/-}$ and *TMEM67*$^{-/-}$ spinal organoids at day 14. Quantifications show the percentages of HOXA5, HOXA7 and HOXC8 positive nuclei per organoid. Data are shown as median with quartiles. Statistics: unpaired t test with Welch's correction (HOXC8) and Mann-Whitney tests (HOXA5, HOXA7). **h** qPCR analysis of *PHOX2B* in *TMEM67*$^{+/-}$ and *TMEM67*$^{-/-}$ spinal organoids at day 14. Statistics: Mann-Whitney test. **i** Immunofluorescence of PHOX2B in *TMEM67*$^{+/-}$ and *TMEM67*$^{-/-}$ spinal organoids at day 14. Quantifications show the percentage of PHOX2B positive nuclei per organoid. Data are shown as median with quartiles. Statistics: unpaired t tests with Welch's correction ($p < 0.0001$). **a-i** N: number of independent experimental replicates. N=3 for both clones in c, d, e, g, h and i; N=3 for both clones in f at day 14 and N=1 for both clones in f at day 4. Scale bars: 50 µm in a, b, e; 150 µm in g, i. For magnified areas (white squares): Scale bars: 5 µm in a, b.

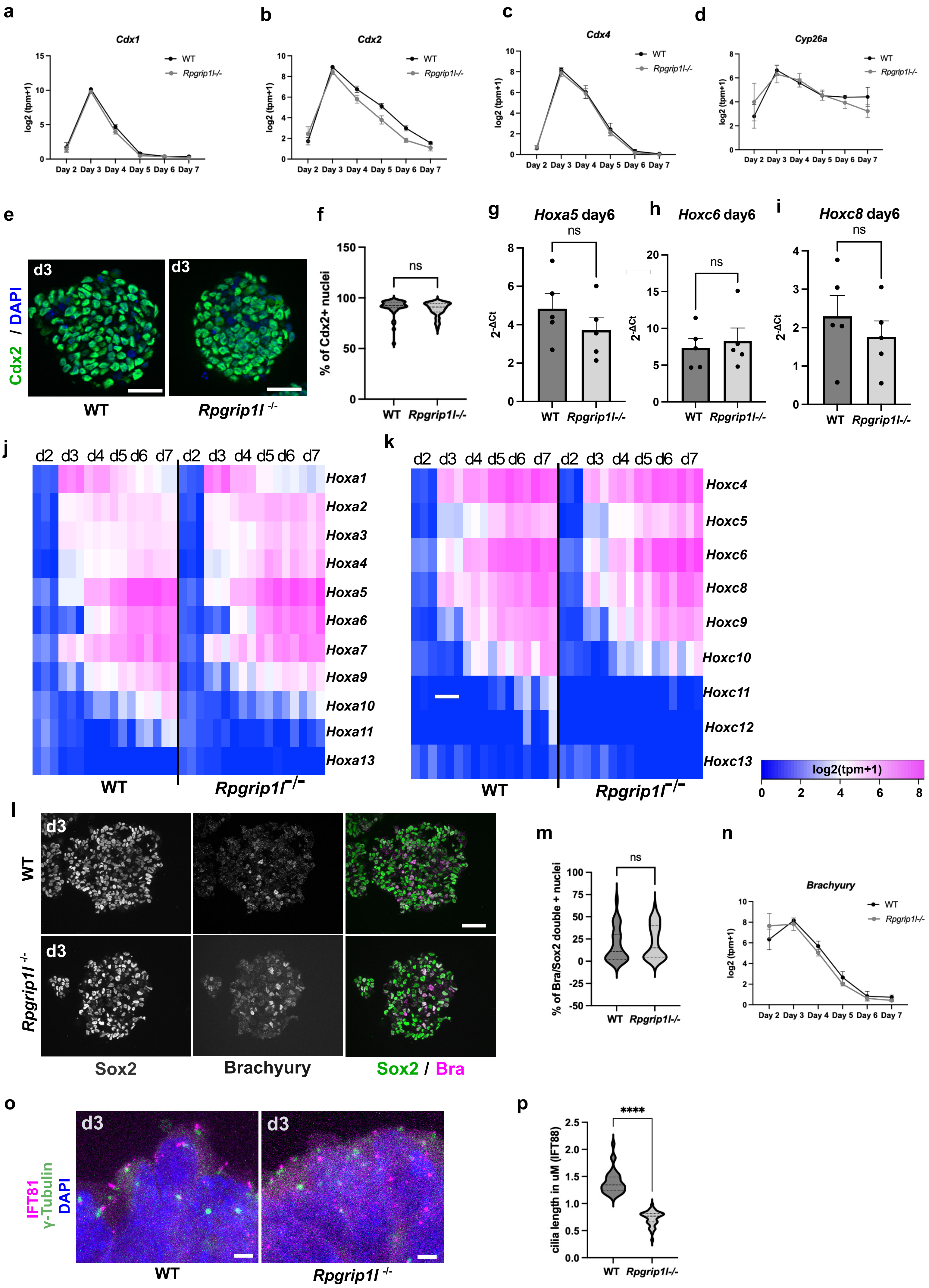

a
Cdx1
WT
Rpgrip1l-/-
log2 (tpm+1)
Day 2 Day 3 Day 4 Day 5 Day 6 Day 7
b
Cdx2
c
Cdx4
d
Cyp26a
e
d3
Cdx2 / DAPI
WT
Rpgrip1l -/-
f
% of Cdx2+ nuclei
ns
g
Hoxa5 day6
2-ΔCt
h
Hoxc6 day6
i
Hoxc8 day6
j
d2 d3 d4 d5 d6 d7
Hoxa1
Hoxa2
Hoxa3
Hoxa4
Hoxa5
Hoxa6
Hoxa7
Hoxa9
Hoxa10
Hoxa11
Hoxa13
k
Hoxc4
Hoxc5
Hoxc6
Hoxc8
Hoxc9
Hoxc10
Hoxc11
Hoxc12
Hoxc13
log2(tpm+1)
l
Sox2
Brachyury
Sox2 / Bra
m
% of Bra/Sox2 double + nuclei
n
Brachyury
o
IFT81
γ-Tubulin
DAPI
p
cilia length in uM (IFT88)
****

Supplementary Figure 10: Mouse Rpgrip1l-deficient spinal organoids do not display spinal to cranial specification defects. a-d, **n** Temporal analysis of *Cdx1, Cdx2, Cdx4* (spinal identity), *Cyp26a1* (cranial identity) and *Brachyury* (NMPs) expression in the course of the differentiation of WT and *Rpgrip1l*$^{-/-}$ organoids. Log2(tpm+1) data from bulk RNASeq analysis are displayed as mean ± SEM (N=3 for each genotype). **e** Immunofluorescence for Cdx2 on sections from WT and *Rpgrip1l*$^{-/-}$ organoids at day 3. **f** Percentage of nuclei positive for Cdx2 in WT and *Rpgrip1l*$^{-/-}$ day 3 organoids. Data are shown as violin plots, median quartiles are indicated as dotted lines. Statistics: two-sided unpaired t test with Welch's correction. N=3 independent experiments, n=1 clone per genotype. **g-i** qPCR analyses of indicated *Hox* genes from WT and *Rpgrip1l KO* organoids at day 6. Data are displayed as mean ± SEM. Statistics: two-sided Mann-Whitney test. N=5 independent experiments; n=1 clone per genotype. **j, k** Heatmap depicting the expression log2(tpm+1) of selected *Hox* genes in WT and *Rpgrip1l KO* spinal organoids over time. **l** Immunofluorescence of Sox2 and Brachyury in WT and Rpgrip1l$^{-/-}$ organoids at day 3. **m** Percentage of nuclei double positive for Sox2 and Brachyury in WT and *Rpgrip1l*$^{-/-}$ day 3 organoids. Data are shown as violin plots, median quartiles are indicated as dotted lines. Statistics: two-sided unpaired t test with Welch's correction. N=2 independent experiments; n=1 clone per genotype. **o** Immunofluorescence for the indicated ciliary markers on sections of day 3 WT and *Rpgrip1l*$^{-/-}$ organoids. **p** Quantifications of ciliary staining. Data shown are mean ± SEM. Statistics: unpaired t tests with Welch's correction ($P < 0.0001$). N=2 independent experiments. Scale bars, 10 µm in e and l.2 µm in o.

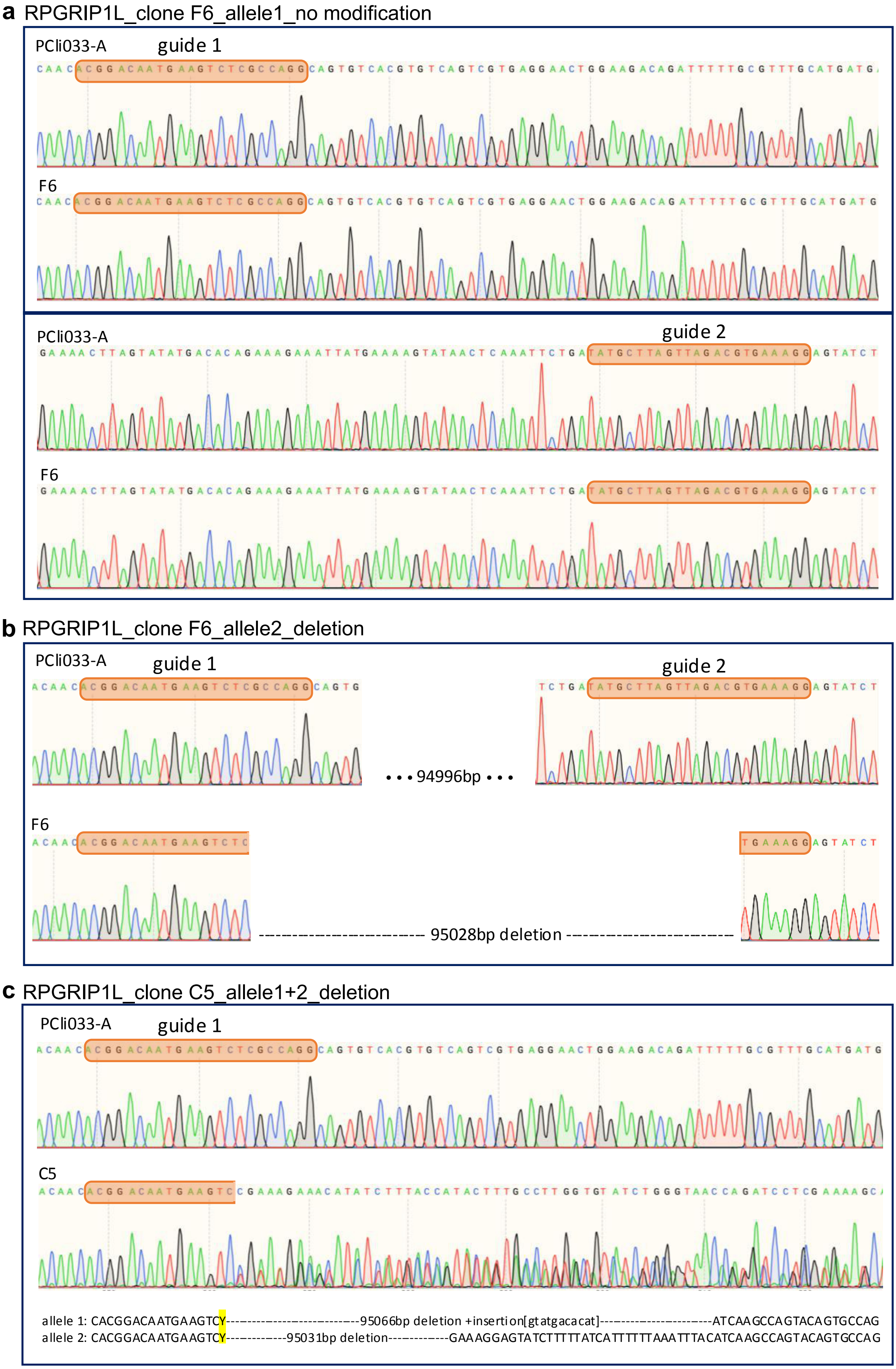
a RPGRIP1L_clone F6_allele1_no modification
PCli033-A
guide 1
F6
PCli033-A
guide 2
F6
b RPGRIP1L_clone F6_allele2_deletion
PCli033-A
guide 1
guide 2
• • • 94996bp • • •
F6
95028bp deletion
c RPGRIP1L_clone C5_allele1+2_deletion
PCli033-A
guide 1
C5
allele 1: CACGGACAATGAAGTCY--------------------------95066bp deletion +insertion[gtatgacacat]-----------------------ATCAAGCCAGTACAGTGCCAG
allele 2: CACGGACAATGAAGTCY-------------95031bp deletion-------------GAAAGGAGTATCTTTTTATCATTTTTTAAATTTACATCAAGCCAGTACAGTGCCAG

Supplementary Figure S11: Sequencing chromatograms for the full-deletion RPGRIP1L hiPSC clones. **a-c** Sequencing chromatograms for clone F6 (HET) and C5 (KO) compared to the PCli033-A control sequence. CRISPR-guides targeting exon 3 (guide 1) and exon 27 (guide 2) are highlighted in orange. **a** The WT allele of clone F6 shows no modification. **b** The mutant allele of clone F6 shows a full deletion of 95028 bps. **c** The KO clone C5 harbors big deletions on both alleles. On one allele 95031 bps are deleted, on the other allele 95066 bps were deleted with a random insertion of 11 bps.

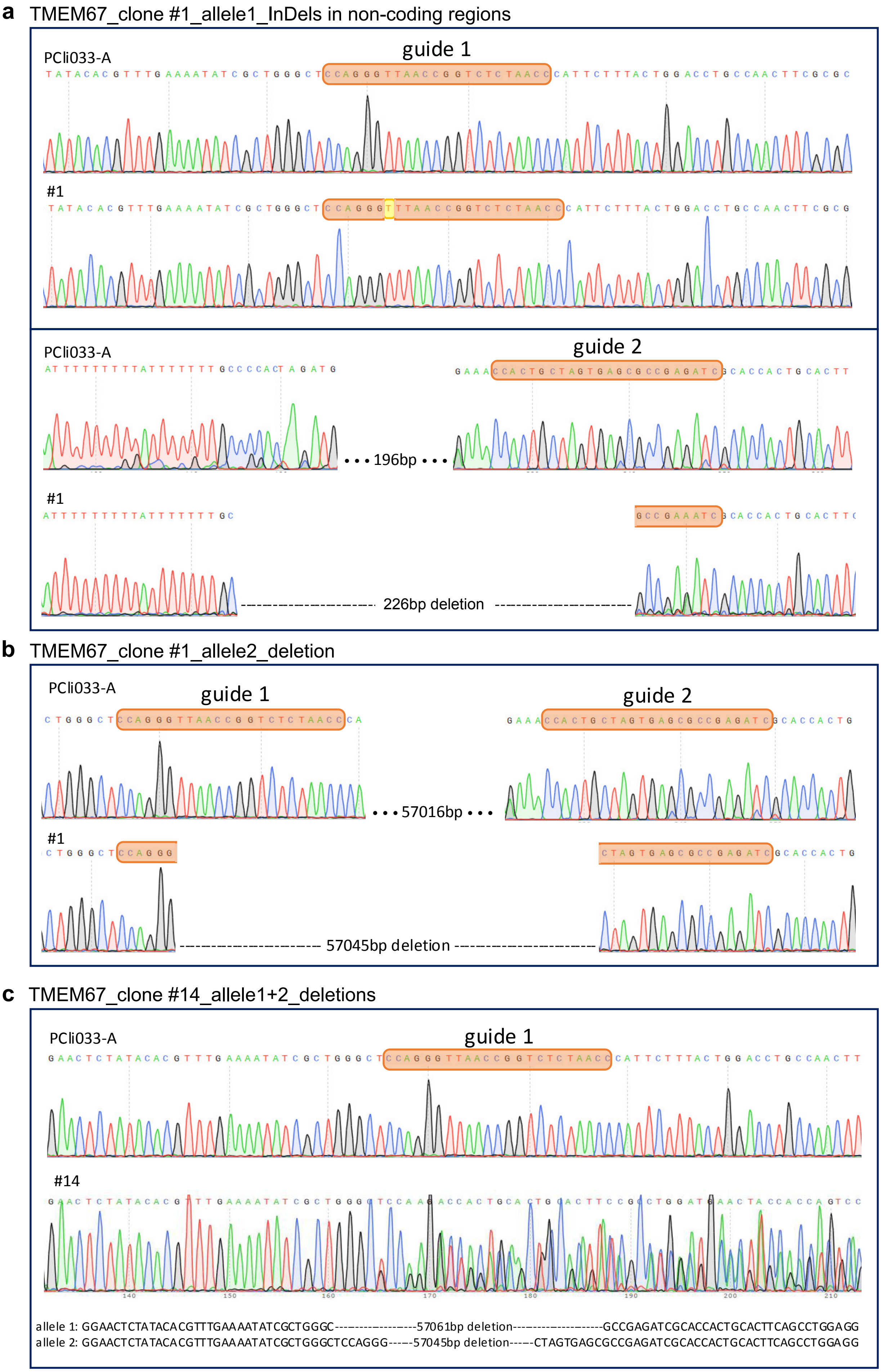
a
TMEM67_clone #1_allele1_InDels in non-coding regions
PCli033-A
guide 1
#1
PCli033-A
guide 2
196bp
#1
226bp deletion
b
TMEM67_clone #1_allele2_deletion
PCli033-A
guide 1
guide 2
57016bp
#1
57045bp deletion
c
TMEM67_clone #14_allele1+2_deletions
PCli033-A
guide 1
#14
allele 1: GGAACTCTATACACGTTTGAAAATATCGCTGGGC-----------------57061bp deletion-----------------GCCGAGATCGCACCACTGCACTTCAGCCTGGAGG
allele 2: GGAACTCTATACACGTTTGAAAATATCGCTGGGCTCCAGGG-----57045bp deletion-----CTAGTGAGCGCCGAGATCGCACCACTGCACTTCAGCCTGGAGG

Supplementary Figure S12: Sequencing chromatograms for the full-deletion TMEM67 hiPSC clones. a-c Sequencing chromatograms for clone #1 (HET) and #14 (KO) compared to the PCli033-A control sequence. CRISPR-guides targeting the 5'UTR (guide 1) and intron 27 (guide 2) are highlighted in orange. **a** The WT allele of clone #1 shows an 1bp insertion (highlighted in yellow) in a non-coding region of the 5'UTR and a deletion of 226 bp in a non-coding region in intron 27. **b** The mutant allele of clone #1 shows a deletion of 57045 bps. **c** The KO clone #14 harbors big deletions on both alleles. On one allele 75045 bps are deleted, on the other allele 75061 bps.

qPCR			
**Gene**	**species**	**Forward**	**Reverse**
GLI1	human	CCCAGTACATGCTGGTGGTT	GCTTTACTGCAGCCCTCGT
PTCH1	human	GATGGGGCCATCTCCACATT	CGCCGCAAAGAAGTACCTTACA
ISLET1	human	TGTTTGAAATGTGCGGAGTG	GCATTTGATCCCGTACAACC
HOXA5	human	GGAGATCATAGTTCCGTGAGC	GCTGAGATCCATGCATTGT
HOXA7	human	CCCTGGATGCGGTCTTCA	CCTTCGTCCTTATGCTCTTTCT
HOXC6	human	ACAGACCTCAATCGCTCAGG	GTACCGCGAGTAGATCTGGC
HOXC8	human	CCTCCGCCAACACTAACAGT	CAAGGTCTGATACCGGCTGT
PHOX2B	human	AACCCGATAAGGACCACTTTTG	AGAGTTTGTAAGGAACTGCGG
GAPDH	human	CAACGGATTTGGTCGTATTGG	GCAACAATATCCACTTTACCAGAGTTA
Gli1	mouse	TTATGGAGCAGCCAGAGAGA	GAGCCCGCTTCTTTGTTAAT
Ptch1	mouse	TGACAAAGCCGACTACATGC	AGCGTACTCGATGGGCTCT
Hoxa5	mouse	CAGATCTACCCCTGGATGCG	GTCTGGTAGCGAGTGTAGGC
Hoxc6	mouse	ACACAGACCTCAATCGCTCAG	CGAGTTAGGTAGCGGTTGAAG
Hoxc8	mouse	CGTCTCCCAGCCTCATGTTT	CGGCGCTTTCTGGTCAAATA
Olig2	mouse	TCCCCAGAACCCGATGATCTT	CGTGGACGAGGACACAGTC
Dbx1	mouse	CAACAGACCCACCAC	AGGAGCTGGCACTCT
Tbp	mouse	AGAACAATCCAGACTAGCAGC	GGGAACTTCACATCACAGCTC
**Sequencing**			
**Gene_region**	**on/off-target**	**Forward**	**Reverse**
RPGRIP1L_exon3	on-target	TTTTATCCTCCTGCTTGCGG	AAGAACGGCACTTGAATACATTT
RPGRIP1L_exon27	on-target	TGTAATGGTGACCCAGGGATGT	AGAAGGAACATAGCCTGTTTGC
TMEM67_5'UTR	on-target	TGGGTTTTAAGTCAGTGGGGT	GTTGTTGTCGCACTTCTCCG
TMEM67_intron27	on-target	TGAAGTGGGACACAGTGGAAG	ATCCATGTTCATTGGCAGTGG
NPRL2_exon8	off-target	CAAGTCCCTGCAAGAGGCAT	CTCTGCTCTTCCCGAGTCAC

Supplementary Table 1: List of qPCR and sequencing primers.

primary Antibody	host	dilution IF	dilution WB	company	reference
Ac3	rabbit	1:500	/	Abcam	ab125093
Arl13b	mouse IgG2a	1:400	/	Antibody Incorporated	75-287
Brachyury	rabbit	1:500	/	Cell Signaling/Ozyme	81694
Brachyury	goat	1:500	/	R&D systems	AF2085
Cdx2	rabbit	1:1000	/	Abcam	ab76541
Gpr161	rabbit	1:200	/	Proteintech	13398-1-AP
Hoxa5	rabbit	1:300	/	Sigma	HPA029319
Hoxa7-CoraLite®	mouse IgG1	1:250	/	Proteintech	CL488-67112
Hoxc8	rabbit	1:300	/	Sigma	HPA028911
Ift81	rabbit	1:200	/	Proteintech	11744-1-AP
Ift88	rabbit	1:300	/	Proteintech	13967-1-AP
Inpp5e	rabbit	1:300	/	Proteintech	17797-1-AP
Islet1/2	mouse IgG2b	1:50	/	DSHB	39.4D5
Nkx2.2	mouse IgG2b	1:50	/	DSHB	74.5A5
Nkx6.1	mouse IgG1	1:50	/	DSHB	F55A12
Olig2	rabbit	1:200	/	Millipore	AB9610
Pax6	rabbit	1:200	/	BioLegend	PRB-278P
Pax6	mouse IgG1	1:50	/	DSHB	PAX6
FoxA2	mouse IgG1	1:50	/	DSHB	4C7
Phox2b	rabbit	1:500	/	kindly provided by JF Brunet	/
RPGRIP1L	rabbit	1:300	/	homemade anti-human	against human RID-domain (Uniprot:A0A087WX34)
Rpgrip1l	rabbit	1:300	1:1000	homemade anti-mouse	against mouse RID-domain (PMID:26150391)
Sox2	rabbit	1:200	/	Millipore	AB5603
Sox2	mouse	1:200	/	Abcam	ab79351
Tmem67	rabbit	1:200	/	Proteintech	13975-1-AP
Tuj1	mouse IgG2a	1:300	/	BioLegend	MMS-435P
γ-Tubulin	mouse IgG1	1:200	/	Sigma	T6557
Actin	mouse IgG2a	/	1:2000	Sigma	A4700
**secondary Antibody**	**host**	**dilution IF**	**dilution WB**	**conjugate**	**company**
Mouse IgG1	goat	1:400	/	Alexa488	Molecular Probes
Mouse IgG1	goat	1:400	/	Alexa633	Molecular Probes
Mouse IgG2a	goat	1:400	/	Alexa488	Molecular Probes
Mouse IgG2a	goat	1:400	/	Alexa594	Molecular Probes
Mouse IgG2a	goat	1:400	/	Alexa633	Molecular Probes
Mouse IgG2b	goat	1:400	/	Alexa488	Molecular Probes
Mouse IgG2b	goat	1:400	/	Alexa594	Molecular Probes
Mouse IgG2b	goat	1:400	/	Alexa633	Molecular Probes
Rabbit	goat	1:400	/	Alexa488	Molecular Probes
Rabbit	goat	1:400	/	Alexa594	Molecular Probes
Rabbit	goat	1:400	/	Alexa633	Molecular Probes
HRP anti mouse	goat	/	1:5000	Jackson Immuno	115-035-003
HRP anti rabbit	goat	/	1:5000	Jackson Immuno	111-035-003

Table S2: List of primary and secondary antibodies.

**DESEQ DATA**	**day 0**		**day 2**		**day 4**		**day 6**		**day 9**		**day 11**		**day 14**	
**Gene**	**FoldChange**	**adj. p**	**FoldChange**	**adj. p**	**FoldChange**	**adj. p**	**FoldChange**	**adj. p**	**FoldChange**	**adj. p**	**FoldChange**	**adj. p**	**FoldChange**	**adj. p**
DBX2	-0.186941274465912	0.999992755782949	-0.0371174313205926	NA	1.41884199840511	0.999843504947457	0.0925065751490877	0.520472144065023	0.00798085564373704	NA	0.110790002483988	0.984356380322919	-0.0751452122754046	NA
OLIG2	-0.315806948843472	0.999992755782949	0.0353996124881901	NA	0.681514889354778	0.999843504947457	-0.710789498123939	0.0642618100823034	-0.40666840813864	0.909425652825697	0.31602357877684	0.984356380322919	0.643323797608997	0.6714026303798
NKX6.1	0.926822337069594	0.999992755782949	-0.00734289364558429	0.999928945743403	0.705245466655836	0.425437058697156	**-0.596060127469382**	**0.00878676629831874**	-0.175434500469697	0.934823395808711	0.222052033077543	0.984356380322919	1.10416660708151	0.0649845505305754
NKX2.2	0.094863909983811	0.999992755782949	0.050284433923495	NA	2.41443331713791	0.347702134407433	0.0322148067573956	NA	0.0102039222413872	0.993191468250953	0.384196617234371	0.984356380322919	0.635067717328612	0.709330139729274
TUJ1 TUBB3	0.137419404338622	0.999992755782949	-0.241483869308178	0.999928945743403	-0.0317683502722379	0.999843504947457	-0.525631334403643	0.257496529147253	-0.273694825533952	0.909425652825697	0.0973380821153323	0.984356380322919	-0.0677623698331345	0.957692938590244
MAP2	-0.205775069959076	0.999992755782949	-0.107771137786243	0.999928945743403	-1.34152243969827	0.888767644191176	0.198802426775524	0.685293011358499	-0.322253307368812	0.876441959646297	-0.435520269274038	0.984356380322919	-0.35161347304583	0.636772747642511
GLI1	0.107348391158355	0.999992755782949	-0.0657061741286489	0.999928945743403	-0.088445754450264	0.999843504947457	-0.30702247779136	0.240184014366194	-0.0700387962704957	0.964144518779646	0.756509009289376	0.984356380322919	1.25234803107015	0.170444862368604
PTCH1	0.45158405963572	0.999992755782949	0.316136534051952	0.999928945743403	-0.610939002622102	0.526213188629742	0.139949286836129	0.755727717404031	0.22359142946598	0.909425652825697	0.555812858617782	0.984356380322919	0.697152660458381	0.20863919375222
HOXA5	0.12563110418162	NA	-0.50323901579525	0.999928945743403	-2.46522110874707	0.0516310734416811	**-1.22052574538796**	**5.34932913867271e-05**	-0.659192644229813	0.512431547924915	-0.556398599108139	0.984356380322919	-1.14892543149702	0.220367113736823
HOXA7	0.115727942300252	NA	-0.101438888914404	0.589603710550248	**-5.41237120715277**	**0.000289639782485042**	**-1.10057452368697**	**0.00513933472916255**	-0.335188007070526	0.909425652825697	-0.757916994373097	0.984356380322919	**-2.39220225997012**	**0.000113901800744846**
HOXC4	0.309850083082869	NA	-0.781166324093198	0.289239960890192	**-2.84180872688252**	**0.0180296345543299**	-0.392577233337781	0.392067033039252	-0.229408453740531	0.909425652825697	-0.302799164750074	0.984356380322919	-0.415440681607327	0.496103835862732
HOXC8	0.134251027070905	NA	-0.161941762212616	0.257011344792264	**-3.42079467012008**	**0.0458139095374785**	-0.592252430538549	0.135011324244442	-0.214902101465428	0.909425652825697	-0.414774824152828	NA	-0.917966761409756	0.438594662068128
PHOX2B	0.0337333402953211	0.999992755782949	-0.0348785242974024	NA	2.8555904165501	0.84630645617989	0.0461541607689378	NA	0.0172542939917648	NA	0.0945930199588227	0.984356380322919	0.116424616601268	NA
TBX20	0.124669956005961	0.999992755782949	-0.0187197862219268	0.787996701551123	-0.420732376159818	0.781136936718622	-0.0342122727940005	0.620139992890314	-0.0452668765242352	NA	-0.11061288090662	NA	0.183415733869837	NA
CDX2	0.248607089675479	NA	**-1.29943426525191**	**0.00107579128565606**	-2.24652428006032	0.0832225387857653	-0.355726588106836	0.541570770017432	-0.345253143672659	0.909425652825697	-0.596642769504026	0.984356380322919	-0.614630377234082	0.708967991930806
ISLET1	0.948841702088542	0.999992755782949	0.00547649056272241	0.961213551665762	-0.0344067010368778	0.993616793241723	-0.0546859218175328	0.720957207140202	-0.681482383121564	0.36912714792612	-0.29873023643002	0.984356380322919	**-1.33284026707656**	**0.0276312324956031**
ISLET2	-0.207015658263226	0.999992755782949	-0.00185098043227171	0.989748530403228	-1.44770066333846	0.703986649210152	-0.0615469185698836	0.766300687081257	-0.330733479275233	0.909425652825697	-0.488113893192837	0.984356380322919	-0.124228083384201	0.946707981995983
EGR2 KROX20	0.296477052907205	0.999992755782949	**1.06272480247936**	**0.0486659001565123**	2.9191554168615	NA	0.245643938619576	0.293677834028938	-0.104550590356454	NA	0.108892152049011	0.984356380322919	0.390813013872423	NA
FGF3	NA	NA	0.556423648752666	0.735702481996449	3.09182470161746	0.717950804551541	**0.806122850752378**	**0.0129666179559297**	0.2736257309179	NA	0.585547517413279	0.984356380322919	0.0428373237158474	NA
PHOX2A	-0.0380769965609032	0.999992755782949	1.50603030720089	9.46439393204205e-05	1.10018294010939	0.973364091816274	**1.62209651151649**	**3.16195913462227e-06**	0.0661268606019388	0.954087288301341	0.100963222325493	0.984356380322919	0.207277613695511	NA
NKX2.9 NKX2.8	NA	NA	NA	NA	4.03729511300133	NA	0.11809088019409	NA	0.140147176942807	0.935562872784137	0.306529304682024	0.984356380322919	0.254075335345996	0.906275967663814
WNT5A	0.787113881184649	NA	-0.68425	0.41630039821902	**-1.98916**	**0.00181394436869744**	0.314788358362476	0.606813448913638	0.0788115575876228	0.964004228261934	0.377608669084226	0.984356380322919	1.24760837961197	0.16500518122207

			6853388062		**822658127**									
CDX1	0.459404032345429	0.999992755782949	-0.291042959350702	0.999928945743403	**-7.30679338027581**	**0.00181394436869744**	**-0.911619632746876**	**0.0223101745987616**	-0.158956667613704	NA	-0.0271890034716007	0.984356380322919	NA	NA
CDX4	0.919662900406647	0.999992755782949	-0.551264535430092	0.999928945743403	**-4.932320037771**	**0.0312312419994349**	-0.0212857342270105	NA	NA	NA	NA	NA	NA	NA
SOX2	0.266022598665522	0.999992755782949	1.00974396122085	0.0718813714513949	-0.00686494109660109	0.999843504947457	0.0291171648749504	0.952562519878802	0.067927804310804	0.962346703868404	0.945147320877804	0.984356380322919	**1.81470621778132**	**0.00621276542119315**
BRA/TBXT	0.866160290851194	0.999992755782949	-0.30583345136879	0.999928945743403	-4.23651604602446	0.0597536628456613	-0.29577379170423	0.488849938833062	-0.0072601218035299	NA	NA	NA	NA	NA

Table S3: Selected DESeq analyses of bulk RNAseq data. FoldChanges and adjusted p-values for gene expressions presented as log(tpm+1) plots in the manuscript are shown for each analysis time point. DESeq2 implements a two-sided Wald test of the Log2FC values. Adjustment of p-values for multiple testing (p-adj) was performed using the Benjamini and Hochberg procedure.
