# Supplementary material for: A differential requirement for ciliary transition zone proteins in human and mouse neural progenitor fate specification
Source: Nat Commun. 2025 Apr 5;16:3258. doi: 10.1038/s41467-025-58554-3 (PMC11972330; doi:10.1038/s41467-025-58554-3)
Supplement: Supplementary file 2 — Reporting Summary [file 41467_2025_58554_MOESM2_ESM.pdf]

nature portfolio

Corresponding author(s): Antonia WIEGERING; Aline STEDMAN; Sylvie SCHNEIDER-MAUNOURY

Last updated by author(s): Mar 11, 2025

Reporting Summary

Nature Portfolio wishes to improve the reproducibility of the work that we publish. This form provides structure for consistency and transparency in reporting. For further information on Nature Portfolio policies, see our Editorial Policies and the Editorial Policy Checklist.

Statistics

For all statistical analyses, confirm that the following items are present in the figure legend, table legend, main text, or Methods section.

n/a	Confirmed	
☐	☒	The exact sample size (*n*) for each experimental group/condition, given as a discrete number and unit of measurement
☐	☒	A statement on whether measurements were taken from distinct samples or whether the same sample was measured repeatedly
☐	☒	The statistical test(s) used AND whether they are one- or two-sided<br>*Only common tests should be described solely by name; describe more complex techniques in the Methods section.*
☒	☐	A description of all covariates tested
☐	☒	A description of any assumptions or corrections, such as tests of normality and adjustment for multiple comparisons
☐	☒	A full description of the statistical parameters including central tendency (e.g. means) or other basic estimates (e.g. regression coefficient) AND variation (e.g. standard deviation) or associated estimates of uncertainty (e.g. confidence intervals)
☒	☐	For null hypothesis testing, the test statistic (e.g. *F*, *t*, *r*) with confidence intervals, effect sizes, degrees of freedom and *P* value noted<br>*Give P values as exact values whenever suitable.*
☒	☐	For Bayesian analysis, information on the choice of priors and Markov chain Monte Carlo settings
☒	☐	For hierarchical and complex designs, identification of the appropriate level for tests and full reporting of outcomes
☒	☐	Estimates of effect sizes (e.g. Cohen's *d*, Pearson's *r*), indicating how they were calculated

Our web collection on statistics for biologists contains articles on many of the points above.

Software and code

Policy information about availability of computer code

Data collection	We did not use any software for data collection
Data analysis	BulkRNASeq analysis:<br>Paired-end RNA reads were aligned against the M. musculus mm39 and the homo sapiens hg38 genome, respectively, by using the STAR aligner (v2.7.10, Galaxy wrapper v4) [144] and the gene annotations gtf files GRCm39.109 and GRCh38.109, respectively. Quality of sequencing was controlled with fastQC (Galaxy wrapper v0.74+galaxy0) and MultiQC (Galaxy wrapper v1.11+galaxy1) [Ewels, P., et al. Bioinformatics., 2016. 32(19): p. 3047-48]. Gene expression was assessed by counting sequencing reads aligned to gene exons with featureCounts (Galaxy wrapper v2.0.3+galaxy2) [Liao, Y., G. Smyth, and W. Shi. Bioinformatics., 2014. 30(7): p. 923-930]. Raw counts were further converted to normalized gene expression values using the log2(tpm+1) transformation where tpm is the count of transcript aligned reads per length of transcript (kb) per million of total mapped reads (Galaxy tool "cpm_tpm_rpk" v0.5.2). Principal Component Analyses (PCA) were performed using r-factominer (v.2.9, Galaxy tool id "high_dimensions_visualisation" v4.3+galaxy0) [Lê, S., J. Josse, and F. Husson. Journal of Statistical Software, 2008. 25(1): p. 1-18] and heatmap visualizations were produced with the Galaxy tool "high_dim_heatmap" (v3.1.3 +galaxy0) using the normalized gene expression values. Differentially expressed genes (DEGs) were selected from the gene raw read counts using DESeq2 (Galaxy wrapper v2.11.40.8+galaxy0) [Love, M., W. Huber, and S. Anders. Genome Biol., 2014. 15(12): p. 550] and the Benjamini-Hochberg p-adjusted cutoff 0.01. DESeq2 statistical tables were used for generation of Volcano Plots (Galaxy tool id "volcanoplot" v0.0.5) [Zink, R., R. Wolfinger, and G. Mann. Clin Trials., 2013. 10(3): p. 398-406], for goseq tests of overrepresented gene ontology categories (Galaxy wrapper v1.50.0+galaxy0) [Young, M., et al. Genome Biol., 2010. 11(2): p. R14], as well as for Ensemble of Gene Set Enrichment Analyses (EGSEA) (Galaxy wrapper v1.20.0) [Alhamdoosh, M., et al. Bioinformatics., 2017. 33(3): p. 414-424].

For manuscripts utilizing custom algorithms or software that are central to the research but not yet described in published literature, software must be made available to editors and reviewers. We strongly encourage code deposition in a community repository (e.g. GitHub). See the Nature Portfolio guidelines for submitting code & software for further information.

Data

Policy information about availability of data

All manuscripts must include a data availability statement. This statement should provide the following information, where applicable:

- Accession codes, unique identifiers, or web links for publicly available datasets
- A description of any restrictions on data availability
- For clinical datasets or third party data, please ensure that the statement adheres to our policy

The RNA sequencing data for this study have been deposited in the European Nucleotide Archive (ENA) at EMBL-EBI under accession number PRJEB86489. Source data are provided with this paper.

Research involving human participants, their data, or biological material

Policy information about studies with human participants or human data. See also policy information about sex, gender (identity/presentation), and sexual orientation and race, ethnicity and racism.

Reporting on sex and gender	All cell lines used in the study, whether mouse Embryonic stem cells or Human iPSCs, were male;
Reporting on race, ethnicity, or other socially relevant groupings	*Please specify the socially constructed or socially relevant categorization variable(s) used in your manuscript and explain why they were used. Please note that such variables should not be used as proxies for other socially constructed/relevant variables (for example, race or ethnicity should not be used as a proxy for socioeconomic status).* *Provide clear definitions of the relevant terms used, how they were provided (by the participants/respondents, the researchers, or third parties), and the method(s) used to classify people into the different categories (e.g. self-report, census or administrative data, social media data, etc.)* *Please provide details about how you controlled for confounding variables in your analyses.*
Population characteristics	*Describe the covariate-relevant population characteristics of the human research participants (e.g. age, genotypic information, past and current diagnosis and treatment categories). If you filled out the behavioural & social sciences study design questions and have nothing to add here, write "See above."*
Recruitment	*Describe how participants were recruited. Outline any potential self-selection bias or other biases that may be present and how these are likely to impact results.*
Ethics oversight	Mouse breeding, mating and embryo collection were performed under ethical agreement number APAFIS-1382 from the French Ministry of Higher Education and Research. The obtaining and manipulation of human iPSC lines were performed under ethical agreements from the French Ministry of Higher Education and Research DC-2021-4734 (for the UCSFi001-A line), DC-2015-2595 and DC-2016-A00773-480 (for the PCIi033-A line) and GMO authorization number DUO-9509.

Note that full information on the approval of the study protocol must also be provided in the manuscript.

Field-specific reporting

Please select the one below that is the best fit for your research. If you are not sure, read the appropriate sections before making your selection.

☒ Life sciences ☐ Behavioural & social sciences ☐ Ecological, evolutionary & environmental sciences

For a reference copy of the document with all sections, see nature.com/documents/nr-reporting-summary-flat.pdf

Life sciences study design

All studies must disclose on these points even when the disclosure is negative.

Sample size	Sample size is indicated in the figure legends for each piece of data presented in this study.
Data exclusions	No data were excluded from the study.
Replication	Information about replication is provided in the figure legends for each piece of data presented in this study.
Randomization	n.a.
Blinding	n.a.

Reporting for specific materials, systems and methods

We require information from authors about some types of materials, experimental systems and methods used in many studies. Here, indicate whether each material, system or method listed is relevant to your study. If you are not sure if a list item applies to your research, read the appropriate section before selecting a response.

Materials & experimental systems

n/a	Involved in the study
☐	☒ Antibodies
☐	☒ Eukaryotic cell lines
☒	☐ Palaeontology and archaeology
☒	☐ Animals and other organisms
☒	☐ Clinical data
☒	☐ Dual use research of concern
☒	☐ Plants

Methods

n/a	Involved in the study
☒	☐ ChIP-seq
☒	☐ Flow cytometry
☒	☐ MRI-based neuroimaging

Antibodies

Antibodies used

A table of all antibodies used is provided in the supplementary material, Table S2.

Validation

For commercial antibodies, validation is provided on the provider's website (see references in Table S2). For the mouse and human Rpgrip1l antibodies produced as part of this work, the validation is provided by the use of mutant lines (see manuscript).

Eukaryotic cell lines

Policy information about cell lines and Sex and Gender in Research

Cell line source(s)

As for human iPSCs: The PCIi033-A line was provided by PHENOCELL (PCI, reference number PCi_CAU2). The UCSFi001-A line (also called WTC11) is deposited at the Coriell Institute for Medical Research under the identifier GM25256. Both iPSC lines are males. The study also uses mouse ESC lines produced in the lab, also both males as determined by PCR analysis. Using male cell lines in all the experiments in the study avoids sex bias in the analysis, in particular in RNAseq data.

Authentication

iPSCs displayed stem cell properties (expression of stemness markers seen in transcriptome) and were able to form EBs and differentiate into neural progenitors and neurons (see manuscript).

Mycoplasma contamination

Cells lines were tested negative for mycoplasma contamination at the beginning and end of the study and at regular intervals during the study.

Commonly misidentified lines
(See ICLAC register)

No commonly miidentified line was used in this study

Plants

Seed stocks

Report on the source of all seed stocks or other plant material used. If applicable, state the seed stock centre and catalogue number. If plant specimens were collected from the field, describe the collection location, date and sampling procedures.

Novel plant genotypes

Describe the methods by which all novel plant genotypes were produced. This includes those generated by transgenic approaches, gene editing, chemical/radiation-based mutagenesis and hybridization. For transgenic lines, describe the transformation method, the number of independent lines analyzed and the generation upon which experiments were performed. For gene-edited lines, describe the editor used, the endogenous sequence targeted for editing, the targeting guide RNA sequence (if applicable) and how the editor was applied.

Authentication

Describe any authentication procedures for each seed stock used or novel genotype generated. Describe any experiments used to assess the effect of a mutation and, where applicable, how potential secondary effects (e.g. second site T-DNA insertions, mosiacism, off-target gene editing) were examined.
