# Supplementary material for: A differential requirement for ciliary transition zone proteins in human and mouse neural progenitor fate specification
Source: Nat Commun. 2025 Apr 5;16:3258. doi: 10.1038/s41467-025-58554-3 (PMC11972330; doi:10.1038/s41467-025-58554-3)
Supplement: Supplementary file 4 — Source Data [file 41467_2025_58554_MOESM4_ESM.zip › source data_westen blot.pdf]

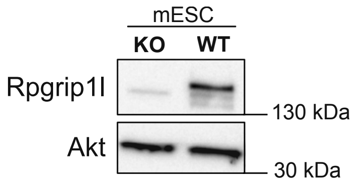
mESC
KO
WT
Rpgrip1l
Akt
130 kDa
30 kDa

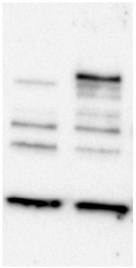

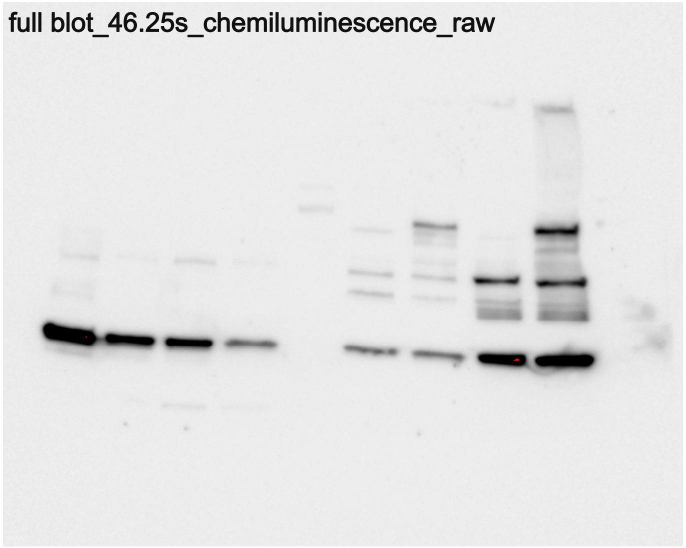
full blot_46.25s_chemiluminescence_raw

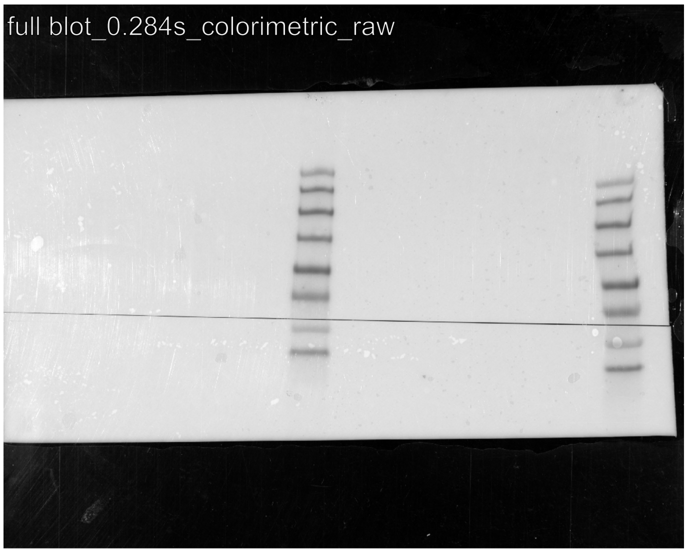
full blot_0.284s_colorimetric_raw

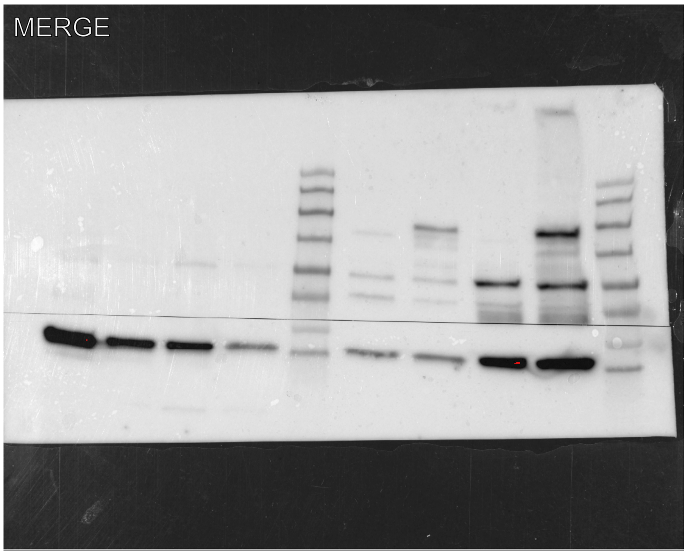
MERGE

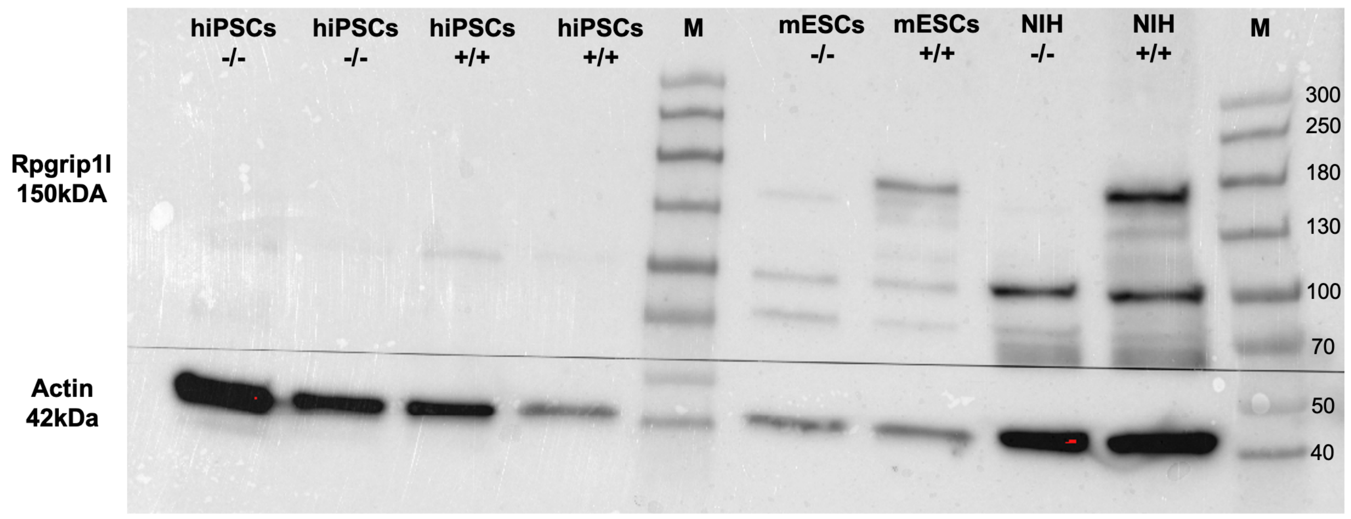
hiPSCs -/-
hiPSCs -/-
hiPSCs +/+
hiPSCs +/+
M
mESCs -/-
mESCs +/+
NIH -/-
NIH +/+
M
Rpgrip1l 150kDA
Actin 42kDa
300
250
180
130
100
70
50
40

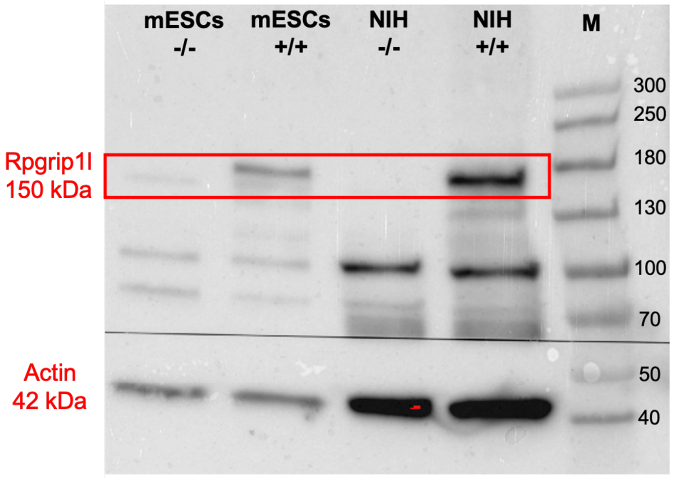
mESCs -/-
mESCs +/+
NIH -/-
NIH +/+
M
Rpgrip1l 150 kDa
Actin 42 kDa
300
250
180
130
100
70
50
40

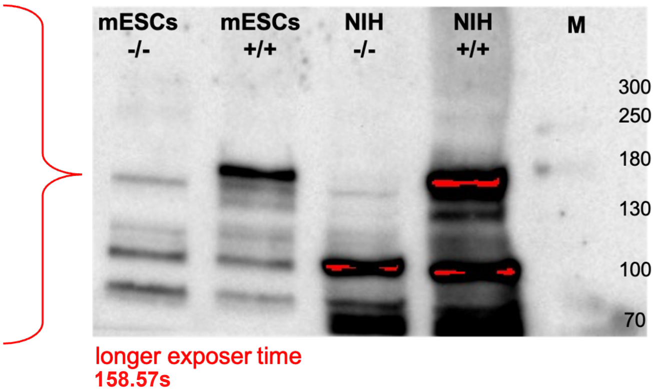
mESCs -/-
mESCs +/+
NIH -/-
NIH +/+
M
300
250
180
130
100
70
longer exposer time
158.57s
