# Supplementary material for: A differential requirement for ciliary transition zone proteins in human and mouse neural progenitor fate specification
Source: Nat Commun. 2025 Apr 5;16:3258. doi: 10.1038/s41467-025-58554-3 (PMC11972330; doi:10.1038/s41467-025-58554-3)
Supplement: Supplementary file 3 — Transparent Peer Review file [file 41467_2025_58554_MOESM3_ESM.pdf]

nature portfolio

Peer Review File

A differential requirement for ciliary transition zone proteins in human and mouse neural progenitor fate specification

Corresponding Author: Dr Antonia Wiegering

This file contains all reviewer reports in order by version, followed by all author rebuttals in order by version.

Version 0:

Reviewer comments:

Reviewer #1

(Remarks to the Author)
The manuscript by Wiegering et a al, is an interesting and well written analysis of the role of RPGRIP1L, and broadly the ciliary transition zone, in development of neurons in the spinal cord of mice and humans using iPSC organoid models. The experiments are well designed and overall, the data and figures provide compelling evidence of differential effects in the 2 mammalian models. The authors use multiple markers and experiments to show that RPGRIP1L loss in human progenitor cells has distinct ciliopathy related effects compared to mouse progenitor cells. These findings are important for understanding the pleiotropic effects of cilia mutations in human patients compared to results from mouse models, as well as identifying RPGRIP1L role in antero-posterior patterning.

Comments:

There are no statistics reported for data in Figure 2a that confirm that WT expression is significantly increased, that KO is not different, or from each other as indicated. “Consistently, while expression of direct targets of the pathway, Gli1, Patched1 and Gli2 was upregulated in controls from day 2, upon SAG addition into the EB culture medium, their expression barely increased in the mutants, showing impaired activation of SHH transduction in absence of Rpgrip1l (Figure 2A, B, C).”
Figure 2E, it is unclear what the mean % of cilia refers to. Is this percentage of ciliated cells?
For Figure 2F, I suggest indicating that cilia length is determined by IF88 length on the figure.
Showing some data confirming similarity of phenotypes between the hiPSCs beyond RPGRI1L loss would be useful.
While the authors show that they generated several clones with RPGRIP1L loss, it is unclear which hiPSC line was used for the main experiments and why it was chosen. Was the same cell line used for all experiments shown or were they mixed? The Authors should clarify this, and figures would be improved with labeling the cell lines on them.

Comparison of the heterozygous deletion to the parental line for some ciliary markers would help confirm there is not an effect of loss of one allele.

Results for figure 3D should come before Figure 3E.

Immunofluorescence for HOXA7 and HOXC4 would be informative to fully understand changes given that the mRNA levels may not relate to protein levels considering HOXA5 results.

Reviewer #2

(Remarks to the Author)
The study by Wiegering and colleagues aims to understand the role of two cilia TZ proteins and SHH activation in neural specification during spinal cord development using spinal organoids, as well as the differences between mouse and human. While the study is interesting and discovers a new potential role of cilia/SHH in rostro-caudal fate acquisition during spinal

cord development, it seems that the work opens more questions than the ones it answers. Why is cilia/SHH critical for MN specification in mouse but not in human?, and why are they essential for rostro-caudal fate acquisition in human but not in mouse? Given that the phenotypes of two structural cilia protein KOs do not fully overlap, should we talk about specific cilia TZ proteins instead of cilia? How is it that RPGRIP1L loss impedes the formation of cilia in NMPs but not in the NPs generated from them? These are all very interesting questions, and some of them are nicely debated in the discussion, but in my opinion, the authors should have focused only on a couple of them and dug dipper.

Major criticisms:

1. Quantifications of the level of RPGRIP1L protein in all the mouse and hiPSC lines used in the study must be shown to prove that they are indeed KOs. The only quantifications shown in the paper are on Fig 4D and S2C and they are based on immunofluorescence of unknown number of cells, organoids and experiments.

2. It´s not clear why the authors spent the effort and time in generating several RPGRIP1L mutant clones (theoretically KO as they claim to be introducing “premature STOP” codons in the sequence) from 2 different hiPSC lines (Fig S2) and yet they don´t use these lines at all in their experiments (only to perform RNAseq), and instead use a single KO clone generated by a different genome editing approach. Some key experiments should be also repeated in those clones, at least in one per line.

3. The claim that the altered rostro-caudal neural specification in the human RPGRIP1L KO spinal organoids is rooted on the lack of cilia in the NMPs is not really supported by causal, functional data but is rather a correlative finding, especially since it´s only shown in 1 hiPSC line (and 1 clone) and only when this TZ protein is missing but not when another TZ protein (TMEM67) is lacking. At least rescue experiments by overexpressing RPGRIP1L at the NMP stage and measuring whether that rescues the abnormal rostro-caudal patterning should be performed.
Also, how do the authors explain that the lack of these two TZ proteins, RPGRIP1L and TMEM67, while having similar functions, as the authors indicate, have such drastically different consequences in NMPs?, meaning, “strong reduction in the cilia number” in the RPGRIP1L KO (Fig. 6H-J – for which quantifications must also be provided) vs no difference in the number of cilia in the TMEM67 KO (quantifications must also be provided).

4. Regarding the species-specific differences of TZ proteins in cilia formation and SHH signaling, despite the mouse organoids have a deficiency of cilia and SHH signaling, the authors claim that the antero-posterior patterning defect upon RPGRIP1L loss is specific to human and not present in mouse organoids. However, to support that statement measurements in mouse organoids of the expression levels and abundance (number of cells) of hindbrain vs spinal markers should be shown, besides the Hox genes shown in Fig S9H-J, which by the way, do not show the most rostral Hox (1-6).

The authors claim that the altered rostro-caudal specification is found in human RPGRIP1L KO organoids but not in mouse, and that it is caused by the lack of cilia specifically and only at the NMP stage. Whether mouse KO NMPs have cilia or not should therefore be shown to clarify whether cilia in NMPs contributes to specify the RC identity in spinal cord development, across species, or whether these TZ proteins have completely different, and unknown, roles in cell specification in mouse and human.

If the authors have access to the ms ESCs, it would be interesting to show whether spinal organoids derived from Tmem67-/- show the same phenotype as the Rpgrip1l-/- ones (no rostro-caudal defects, in principle, but no ventralization). This would help to understand whether it’s a single cilia TZ protein or the cilia itself what determines the RC and DV neural fate in the mouse spinal cord development.

5. It’s somehow surprising that the authors have used quite different protocols for their mouse and human organoids. While for mouse, they go straight for WNT activation simultaneously to the caudalizing and ventralizing signals (RA+SAG), for human, neural induction with SB+LDN is the first step, together with WNT activation and prior to the RA+SAG. As the authors know, time, duration, concentration and combination of patterning molecules is key to guide the differentiation of PSCs into different cell fates. The authors should provide an explanation for such protocol difference.
Also, to generate NMPs from PSCs, for most published protocols a 2-4day pulse of CHIR+FGF2 is needed prior to neural induction (https://doi.org/10.1016/j.xcrm.2024.101659, https://doi.org/10.1016/j.stem.2019.12.007; https://doi.org/10.1242/dev.119768; https://doi.org/10.1371/journal.pbio.1001937 and others), which the authors do not follow. Therefore, to prove that their mouse and human spinal organoids are indeed generated from EBs composed by NMPs, these should, at least, be stained for SOX2 and TBXT at early stages of their development (before day 4 according to their protocols) and the percentage of double positive cells must be shown. In page 7 they claim “Within four days, WT and RPGRIP1L-deficient cells adopted a caudal NMP-like axial fate” but the data supporting that claim is not shown. This data should come early in the manuscript, not in Fig 6.

6. The authors should clearly state the number of independent experiments performed for each figure panel. For example, quantifications for Fig. 1E-G are supposed to be “n sections analyzed ≥8, from ≥2 experimental replicates”. As the authors know, organoids are very heterogeneous both across intra- and inter-experiments. At least 3 different sections from 3 different organoids (n=3) from at least 3 independent experiments (N=3) should be always analyzed to guarantee the robustness and reliability of the data. Regading the mouse RNAseq, was it performed in 3 EBs from the same experiment (n=3) or from 3 independent experiment with multiple EBs each pooled together (N=3)? 3 independent experiments is always the minimum to be able to reach any conclusion. Also, each of those experimental points should be shown separately rather than pooled to show the heterogeneity/robustness of the results.

7. The quantifications of immunostained spinal organoid sections are per “positive area”. Instead, the number of cells

positive for whatever marker over the total number of cells should be quantified (at least definitely for the nuclear markers measured -which is most of them-) to provide accurate quantifications.
What exactly each graph shows is sometimes difficult to grasp. E.g. in Fig 2E (S2C, S3C and others), does "mean %of ARl13b+ cilia" mean the percentage of cells in the organoid with cilia? Or Fig S4F (containing important data to support that the RPGRIP1L KO organoids do not contain an enhanced % of pMNs and yet it's a supplemental figure coming from an unclear N and with no statistics). This graph should show the # cells+ / total #cells because if the organoids have different size or different cell densities, these results do not mean anything.

Also, in relationship with the NKX2.2 results, the authors say "Here, expression of NKX2.2 plays a primary role in ventral neuronal patterning and requires high SHH signaling…", which implies that the specification of NKX2.2- neural types in the ventral spinal cord do not rely on SHH signaling. It seems that the authors used this argument to link this section with the next, on SHH, but does not seem correct.
8. most of the mRNA expression levels shown in the multiple graphs come solely from the RNAseq performed. qPCR from additional independent experiments must be performed as validation of that single RNAseq dataset.
9. To ease the readability and put all results into context, the discussion should not contain separate sections.

Minor comments:
- The authors should acknowledge that a few studies describing the role of ciliary proteins on the establishment of DV patterning via SHH regulation using brain or spinal organoids have been published (e.g. doi: 10.1016/j.celrep.2022.110811, doi: 10.1371/journal.pone.0301670)
- Although of relative importance depending on the intention of the authors, statements like "we could not detect any difference between…" should be replaced by "we did not detect…", because "could not" implies the purpose of finding something specific and that implies bias.
- In page 5, lines 29-30, the statement "Strikingly, Rpgrip1l-deficient progenitors failed to commit to ventral spinal identities" does not seem appropriate, since, unless I misunderstood it, that result was precisely what was expected.
- Fig S1D-E need further explanation because currently they aren´t very informative (the genes in D aren´t readable). What does the color indicate? And how high in the GSEA ranking does SHH signaling and spinal cord patterning appear?
- In page 7, where is the data supporting the claim "Importantly, for each genotype, Wtc11 and 1 Ph2 hiPSC lines behaved the same in terms of differentiation efficiency, cilia phenotype and gene expression profiles"?
- Some figure panels are not referred to in order (e.g. Fig 3E before 3D, 5G before 5F, S4B is cited before S3D-F; and S4D-E don't seem to be cited at all), which makes it confusing to go over the figures.
- In some figures (e.g. Fig 3 and S4A-C) only a small fraction of the organoids is shown. The entire organoids should be shown instead.
- In Fig. 4E, organoid images with only DAPI should be shown to indicate where the ventricle-like structures are located. In addition, no clearARL13B signal is detected in the outside of the organoids shown, so maybe that statement needs to be rephrased in page 9?
- In figure 5E, the statement that the rostral MN marker PHOX2B is expressed in the RPGRIP1L KO organoids but not in WT is incorrect. PHOX2B is also expressed in WT but to a lesser extent. As mentioned before, the RNAseq data must be validated by qPCR in multiple independent experiments.
- Fig S7G is referred to in the text as data on axial progenitors when it seems to refer to spinal organoids. This should be corrected.
- The data shown in Fig 6C (RNAseq) and 6E (immunostaining) does not fit. This should be explained.
- Line 23 in page 12, do the authors mean Hoxd10 instead of HoxPG10?

Reviewer #3

(Remarks to the Author)

Overview and noteworthy results
The study by Wiegering et al. highlights species-specific roles for the transition zone gene RPGRIP1L in spinal cord development, demonstrating that findings in mouse models may not fully capture the complexity of human disease mechanisms. While mouse Rpgrip1L mutants show severe defects in SHH signaling and motor neuron specification, human RPGRIP1L-deficient organoids retain normal SHH signaling and retain cilia, but display a distinct shift in anterior-posterior motor neuron identities. More specifically, human RPGRIP1L-deficient organoids exhibit a shift from thoracic to hindbrain motor neuron identities, marked by altered HOX gene expression, a novel role for RPGRIP1L not seen in mice. This divergence between species underscores the risk of over-reliance on mouse models for understanding human neurodevelopmental diseases. By using human stem cell-derived organoids, this work suggests that human cell lines can offer a more accurate representation of disease phenotypes and are likely vital to our understanding and treating complex diseases like Joubert Syndrome (which can be caused by defects in RPGRIP1L). The findings suggest that using human-specific models could reshape our approach to studying neurodevelopment and developing targeted therapies. I believe the findings in this paper are very interesting. These findings have broad implications not only for ciliopathy research but also for the study of other diseases where species-specific differences might play a critical role.

Will this work be of significance to the field?
It is widely known that ciliopathy phenotypes in mice are more severe than in humans. The best example I can think of is

hydrocephalus, where ciliary defects in mice frequently result in hydrocephalus, but this phenotype is observed to a much lesser degree in humans. This example speaks to motile cilia and may be a product of different brain anatomies. In this paper, through a detailed examination of transition zone mutants, the authors provide compelling evidence of more subtle trafficking (and thus signaling) phenotype differences between mice and humans. I think this would be of high interest to the field and will expand upon our understanding of human ciliopathies.

Does the work support the conclusions and claims?
Yes, the work strongly supports the conclusions and claims made. The data is exceptionally thorough and well-presented. As a developmental biologist, I feel like most studies focus on a snapshot of developmental time. What I found striking about this paper was the detailed transcriptional analysis performed throughout the differentiation process. This time-course transcriptomic analysis provided beautiful insights into how RPGRIP1L deficiency impacts the gene expression landscape, highlighting specific changes in anterior-posterior patterning, including shifts in HOX gene expression and the misspecification of motor neuron identities. The dynamic profiling of gene expression is especially compelling and directly links cilia-related defects to alterations in spinal progenitor differentiation, solidifying the authors' claims. Most surprising to me was the novel finding that human RPGRIP1L-deficient organoids do not exhibit the same SHH signaling defects as mouse models, but instead show unique anterior-posterior patterning shifts. Admittedly, I was skeptical at first, as negative data (i.e. a loss of a defect) is hard to support and can easily be explained by an incomplete knockout or knockdown. However, the analysis of multiple clones and the use of multiple approaches to ultimately create the RPGRIP1L-deficient organoids was compelling.

Is the methodology sound?
My area of expertise is spinal cord development and hedgehog signaling. The methods and analysis in this paper were superb. I am familiar with the cell culture techniques and the detail provided in the methods is more than sufficient to allow others to reproduce the experiments.

Final thoughts
This is a well-written paper and seemingly the exciting beginning of many more studies. I was particularly interested in how much further these species-specific differences exist. For example, perhaps the composition of the mouse and human transition zone are different. In addition, as alluded to in the discussion, it will be very interesting to identify the origin of the anterior-posterior patterning changes – maybe it is due to alterations in Wnt signaling, which would be fascinating because the role of primary cilia in Wnt signaling is still quite a controversial. In conclusion, the paper seems very complete and I support its publication. While perhaps beyond the scope of this study, the only thing that would ask for that I think would complete this study is an analysis of Joubert Syndrome iPSCs, to see if the phenotypes compare to the RPGRIP1L-deficient cells generated in this study. Small point, I was also very puzzled by the result in Figure 3D -- it seems like an outlier on many levels (transient effect and inconsistent with all the other data in the figure).

Version 1:

Reviewer comments:

Reviewer #1

(Remarks to the Author)
The authors have addressed my previous concerns adequately and improved all aspects of the manuscript. The data is more clearly presented with the necessary information to make the study reproducible. Conclusions are supported by their data and not overstated.

Reviewer #2

(Remarks to the Author)
As I wrote in my original review, I found this story interesting, both from the point of view of the conceptual knowledge gained on the role of the cilia in spinal cord NP specification and AP patterning and from the inter-species differences angle. I had, however, lots of comments and concerns back then, and although the authors have clearly made an effort to address them, some of them remained unanswered and their responses to others have raised additional doubts. I firmly believe that fully addressing them would significantly improve the reliability of the data and the robustness of the claims. They are indicated below according to their numbering in the rebuttal letter.

1. Doesn't matter if these mouse cells have been published before (even by this same group), it is a must to show proof that the KO cells one works with indeed lack the protein of interest. We all know that immunostaining quantifications can be totally random (e.g. is the acquisition parameters aren't identical across samples, and especially if the sampling isn't blind), which is much harder to alter in a WB. The WB does not have to replace the immunostaining, both panels should be shown in the figure.

Where are the "sequencing data for each clone" provided? For the above reason, if WB used can't be shown because the AB works for immunostaining but not WB, then at the very least the sequencing data of the targeted clones must be shown. Figure S2b is simply a list of mutations, which, as the authors should understand, is not a proof of anything, especially when a paper is being revised for potential publication and when the whole study is based on these edited cells. The Sanger

sequencing chromatogram aligned to a reference sequence must be shown.
Even RNAseq data showing mRNA expression of RPGRIP1L mRNA in the mutated and KO cells could be shown, like for the mouse cells in Fig. S1b, although, that's just mRNA and not protein, which is what this study cares about.
Also, why the "human specific RPGRIP1L antibody" (3rd blot) detects a band of the right mw in the NIH3T3 cells (mouse fibroblasts)? Either that AB is not human-specific or that band is not RPGRIP1L and therefore the WB shown for the mouse cells unfortunately can't be trusted either.

2. I'm more confused than before because at least the original text said which lines were used for each experiment (even if this was wrongly missing in the figure legends), but in the revised version this information in the text is crossed out and is still missing in the legends, so there is no way to know which data comes from which cell line. For example, all fig. 3 seems to refer to one single WT line (and clone) and one single KO line (and clone), unknown which one, and yet all throughout the figure legend it's written "WT: n=4; KO: n=4", what does that mean?? The exact same happens to every single figure. Or does it mean that the results from all the WT and the KO clones, even from different lines, are combined in the same graphic?, but what is the logic of that then?, why claiming to make multiple clones from multiple lines if all the results will be cluttered into the same plot? This sounds very strange.

Which cell line has been used in each experiment is obviously basic information that should be at least in the legend, if not in the main text too.
The justification of "The small standard deviations show the low variability between individual clones within genotypes and between the two cell lines" does not seem accurate as, in many of the bar graphs present in the revised version (some of which still don't display the independent experimental values) the error bars are several orders of magnitude (e.g. Fig. S4i, n, S7b-e, S9f, etc), and some of the line-based graphs still don't have stats (e.g. Fig. 3d-e, 5b-f, k-l, 6e, etc).

3. I find this response insufficient. Using a lentivirus to overexpress a 150 kDa protein is more than possible, and the time constrains aren't well supported either as the organoids are not analyzed past day 14. If the suggested rescue experiment is not done, 1) that claim can't be made but the idea only proposed, and 2) the new experiment the authors referred to must be solid, although still correlative. Unfortunately, I find these new results very worrisome. In the original version, Fig. 6E showed NO difference in the #CDX2+ cells between RPGRIP1L KO and WT day 4 organoids, however, in the new Fig 6d data the statistical significance has 3 stars?

Much later, and only due to a different (and theoretically minor) comment, the authors somehow justify this surprising change by saying "we included new CDX2 stainings and quantifications at day 2 of differentiation as well as increased the number of clones and experiments analysed at day 4 of differentiation" and by doing that the CDX2 results drastically changed. However, weren't also in the original version all clones analyzed and merged together (strangely as commented above)?, so what more clones are added now? Unfortunately, this also sounds odd.

To the comment: "we correlate the loss of cilia in RPGRIP1L deficient progenitors to a reduced CDX expression at the axial progenitors stage and to an altered specification of the NMP-like (SOX2+/BRA+) fate… We show this correlation in all RPGRIP1L-deficient clones of both cell lines, in total 5 RPGRIP1L- deficient clones (Figure 5 and Figure 6 for RPGRIP1L InDel mutants; Figure S4 for the RPGRIP1L full deletion clone) ". Fig 5 does not mention CDX2, SOX2/T or even day 2-4 organoids, and neither does S4.

5. The authors should still provide an explanation as to why using these patterning molecules in a different order for mouse and human. Saying that you follow published protocols to maximize MN specification isn't enough, as there endless different protocols out there making similar claims. What's the rationale the authors used?, do they know why they used those molecules in that order?

In addition, how can one "convincingly" claim that the human and mouse organoids are comparable in NMP composition when, for example, your day 4 human (WT) organoids are 100% SOX2+ and 100% BRACHYURY+ (e.g. Fig. S9e), however your mouse day 3 organoids show high SOX2 levels (# cells not quantified, DAPI not shown) but Brachyury is barely present? (Fig. S10i)? That simple comparison says that your NMP-made spinal organoids are not "sufficiently comparable". Maybe the mouse analysis should have been done at day 2 instead of at day 3 to have a really comparable human-mouse model.
Also, as the authors should know, NMPs are typically identified by the double expression of SOX2 and TBXT (BRACHYURY) (they actually say in the revised discussion "Later (at day 4), reduced numbers of BRACHYURY+/SOX2+ NMPs are found in RPGRIP1L mutants"). However, no quantification of double+ cells is shown for any of their human organoids, which is shown for their mouse organoids (Fig. S10h) and, strangely measures 0% double positive cells, which could indicate, among other things, that those mouse organoids at day 3 are already committed to neural lineage and therefore aren't NMPs anymore.

7. And are those numbers now 5% or 0.05% (first black bar)? The legend in combination with the Y axis indicates the latter (wrongly, I guess).

Minor:
- The table included in Fig. S1b on "selected" DEG doesn't answer my question of "how high in the GSEA ranking does SHH signaling and spinal cord patterning appear?"

Version 2:

Reviewer comments:

Reviewer #2

(Remarks to the Author)
I thank the authors for the effort made to thoroughly address the latest concerns I raised. I don’t have any further comment.

Open Access This Peer Review File is licensed under a Creative Commons Attribution 4.0 International License, which permits use, sharing, adaptation, distribution and reproduction in any medium or format, as long as you give appropriate credit to the original author(s) and the source, provide a link to the Creative Commons license, and indicate if changes were made.
In cases where reviewers are anonymous, credit should be given to 'Anonymous Referee' and the source.
The images or other third party material in this Peer Review File are included in the article’s Creative Commons license, unless indicated otherwise in a credit line to the material. If material is not included in the article’s Creative Commons license and your intended use is not permitted by statutory regulation or exceeds the permitted use, you will need to obtain permission directly from the copyright holder.
To view a copy of this license, visit https://creativecommons.org/licenses/by/4.0/

Manuscript “A differential requirement for ciliary transition zone proteins in human and mouse neural progenitor cell fate” by Wiegering et al.

RESPONSE TO REVIEWER COMMENTS

We thank the reviewers for their appraisal of our work and for their insightful comments and questions. We believe our work has significantly improved by taking their comments into account.

Reviewer #1 (Remarks to the Author):

The manuscript by Wiegering et a al, is an interesting and well written analysis of the role of RPGRIP1L, and broadly the ciliary transition zone, in development of neurons in the spinal cord of mice and humans using iPSC organoid models. The experiments are well designed and overall, the data and figures provide compelling evidence of differential effects in the 2 mammalian models. The authors use multiple markers and experiments to show that RPGRIP1L loss in human progenitor cells has distinct ciliopathy related effects compared to mouse progenitor cells. These findings are important for understanding the pleiotropic effects of cilia mutations in human patients compared to results from mouse models, as well as identifying RPGRIP1L role in antero-posterior patterning.

Comments:

1. There are no statistics reported for data in Figure 2a that confirm that WT expression is significantly increased, that KO is not different, or from each other as indicated. “Consistently, while expression of direct targets of the pathway, Gli1, Patched1 and Gli2 was upregulated in controls from day 2, upon SAG addition into the EB culture medium, their expression barely increased in the mutants, showing impaired activation of SHH transduction in absence of Rpgrip1l (Figure 2A, B, C).”

We agree with the reviewer that statistics were lacking. We have chosen to show plots of normalized counts versus time to illustrate variations in gene expression during the course of differentiation of WT and *Rpgrip1l* KO organoids. However, there are no appropriate statistical tests to assess the significance of these differences using the log2tpm+1 data, explaining why no statistics were shown on the corresponding figures. As part of our bulk RNAseq analysis, a pairwise comparison between control and mutant organoids at each time point of the differentiation was performed using DEseq2. We now provide a table of selected significantly differentially expressed genes between WT and *Rpgrip1l* KO mouse organoids at day 4, including the log2FC and adjusted p-value. *Gli1* and *Patched1* were added to this list (Figure S1b of the revised manuscript). Moreover, to validate these results, we performed qPCR for *Gli1* and *Patched1* on N=4 independent experiments. Graphs showing the fold change in expression of *Gli1* and *Patched1* in WT and *Rpgrip1l* KO organoids at different time points of the differentiation, as well as the results of the statistical analysis using the Mann-Whitney test, are now shown in Figure 2a, b. The temporal expression profile for these genes based on log2(tpm+1) graphs have been moved to Figure S1g, h.

2. Figure 2E, it is unclear what the mean % of cilia refers to. Is this percentage of ciliated cells?

We agree that the graph legends lacked clarity. Figure 2E (Fig. 2d in the revised version) refers to the percentage of cilia containing Arl13b: using a co-staining for gamma-tubulin and Arl13b proteins, we manually scored the number of gamma-tubulin positive basal bodies topped by Arl13b stained axonemes. To improve this point we have now changed the axis legend: “mean % of cilia” has been changed for “% of Arl13b+ cilia”. We also clarified it in the revised version of the manuscript (p.6 lines 21f).

3. For Figure 2F, I suggest indicating that cilia length is determined by IF88 length on the figure.

We changed the figure accordingly (Figure 2e in the revised manuscript).

4. Showing some data confirming similarity of phenotypes between the hiPSCs beyond RPGRIP1L loss would be useful. While the authors show that they generated several clones with RPGRIP1L loss, it is unclear which hiPSC line was used

for the main experiments and why it was chosen. Was the same cell line used for all experiments shown or were they mixed? The Authors should clarify this, and figures would be improved with labeling the cell lines on them.

We agree with the reviewer that the explanation of which cell lines/clones were used for each experiment and why they were used was not clearly given in the manuscript. We have carefully restructured the text and figure legends to clearly state how many clones were used for each experiment and why we used different KO clones for RPGRIP1L (p.7 lines 1ff; p.9 lines 28ff; p.10 lines 6ff; p.18 lines 28ff). We hope it is now clear that each individual result presented in the main figures is based on the analysis of 4 WT and 4 *RPGRIP1L KO* clones (n=4) generated by *InDel* mutations. Two WT and two KO clones of each iPSC line (PCIi033-A and UCSFi001-A) were used for the experiments to increase genetic background variation. The small standard deviations show the low variability between individual clones within genotypes and between the two cell lines. To provide more details on the variation between clones, we attached below 4 graphs that show the quantification of immunofluorescence data for OLIG2 (related to Figure S3b in the revised version), ISLET1 (related to Figure S3f in the revised version), BRACHYURY (related to Figure S8h in the revised version) and HOXA7 (related to Figure 5g in the revised version) for each analysed clone at specific differentiation stages.

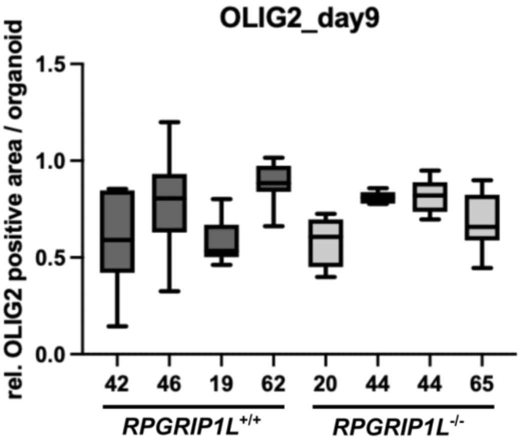

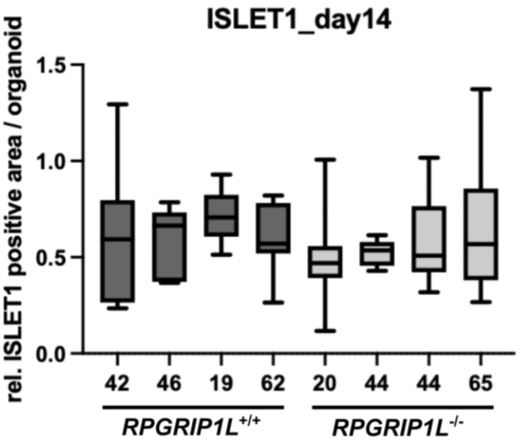

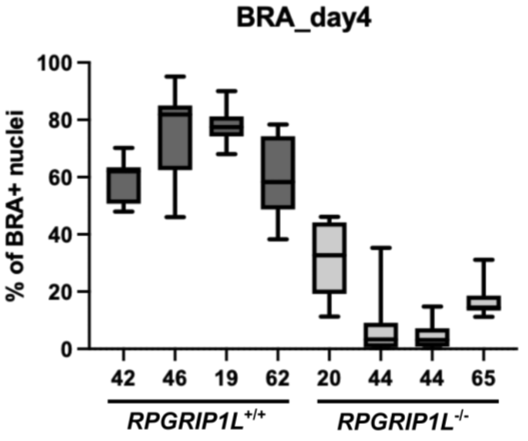

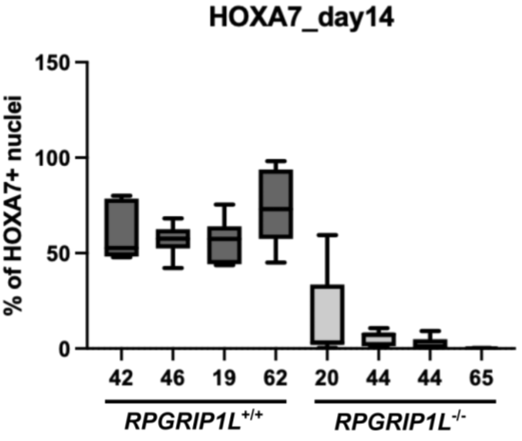

5. Comparison of the heterozygous deletion to the parental line for some ciliary markers would help confirm there is not an effect of loss of one allele.

In the revised version of the manuscript, we compared the ciliary RPGRIP1L amount in heterozygous and homozygous deletion clones to WT controls (Figure S4d) and did not detect a reduction in ciliary RPGRIP1L amount in the heterozygous clone compared to WT controls. Furthermore, measurements of ciliary length (Figure S4e) and density (Figure S4f) show the same values in the heterozygous RPGRIP1L clone compared to WT controls (Figure 4d, e). In addition, we included WT controls in the measurements of ISLET1/2-positive areas per organoid (Figure S4k) to show that the percentage of MNs (ISLET1/2+ cells) is unaltered in heterozygous RPGRIP1L mutants compared to WT.

6. Results for figure 3D should come before Figure 3E.

We thank the reviewer for noticing this incoherence between the text and the figure. We have edited the figure channels (Figure 3e, f, g, h) in correspondence to the text.

7. Immunofluorescence for HOXA7 and HOXC4 would be informative to fully understand changes given that the mRNA levels may not relate to protein levels considering HOXA5 results.

We agree with the reviewer and included HOXA7 immunofluorescence analyses for all clones to the revised version of our manuscript (Figure 5g; Figure S4m; Figure S9g). We also tested other HOX antibodies such as HOXC6 and HOXC9 that unfortunately did not work in our hands.
However, differences between *HOX* mRNA expression and translated HOX protein expression during spinal differentiation of hiPSCs was reported before (Dasen et al., *Nature* 2023, https://doi.org/10.1038/nature02051; Mouilleau et al., *Development* 2021, https://doi.org/10.1242/dev.194514). For example, it was shown that *HOXC6* and *HOXC8* were expressed in spinal organoids at mRNA levels, but only HOXC8 protein levels were detectable at later stages (Mouilleau et al., *Development* 2021, https://doi.org/10.1242/dev.194514).

Reviewer #2 (Remarks to the Author):

The study by Wiegering and colleagues aims to understand the role of two cilia TZ proteins and SHH activation in neural specification during spinal cord development using spinal organoids, as well as the differences between mouse and human.

While the study is interesting and discovers a new potential role of cilia/SHH in rostro-caudal fate acquisition during spinal cord development, it seems that the work opens more questions than the ones it answers. Why is cilia/SHH critical for MN specification in mouse but not in human?, and why are they essential for rostro-caudal fate acquisition in human but not in mouse? Given that the phenotypes of two structural cilia protein KOs do not fully overlap, should we talk about specific cilia TZ proteins instead of cilia? How is it that RPGRIP1L loss impedes the formation of cilia in NMPs but not in the NPs generated from them? These are all very interesting questions, and some of them are nicely debated in the discussion, but in my opinion, the authors should have focused only on a couple of them and dug dipper.

Major criticisms:

1. Quantifications of the level of RPGRIP1L protein in all the mouse and hiPSC lines used in the study must be shown to prove that they are indeed KOs. The only quantifications shown in the paper are on Fig 4D and S2C and they are based on immunofluorescence of unknown number of cells, organoids and experiments.

We performed WB analyses for Rpgrip1l on mESCs [and NIH3T3 cells as controls (Wiegering et al., 2018)] to validate the deletion of Rpgrip1l. The WB result below demonstrates the absence of Rpgrip1l protein in lysates of Rpgrip1l-deficient mESCs using the mouse-specific Rpgrip1l antibody.

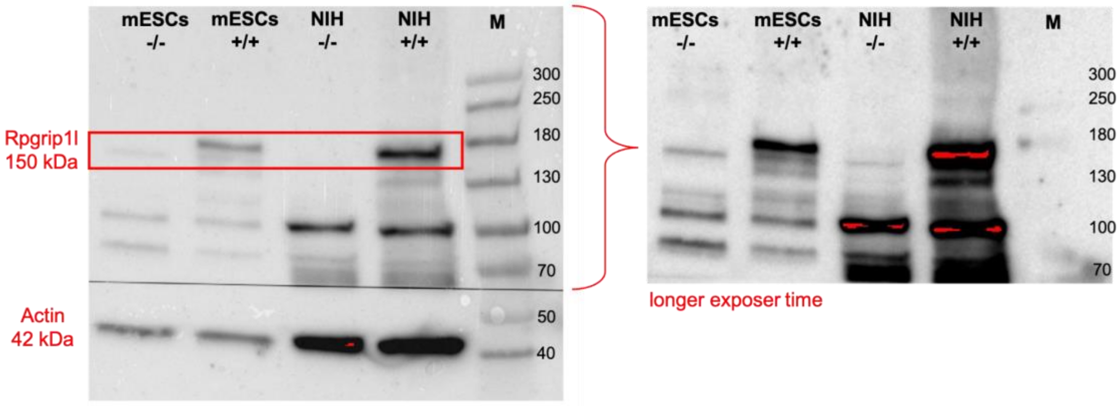

Since the mESCs were isolated from Rpgrip1l-deficient mouse models that were validated and published by us and others (Vierkotten et al., *Development* 2007 https://doi.org/10.1242/dev.003715; Gerhardt et al., *JCB* 2015 https://doi.org/10.1083/jcb.201408060; Wiegering et al., *EMBO* 2018 https://doi.org/10.15252/embj.201797791), we would rather not include WB analyses for the validation of the Rpgrip1l deletion in this manuscript. Moreover, we think that Immunofluorescence analyses that not only validate the absence of Rpgrip1l from the ciliary TZ in mutant cells, but also show its presence at the TZ of WT cell types of interest (Fig. 2c), are more informative here. In addition to the loss of Rpgrip1l protein, *Rpgrip1l* mRNA was found downregulated in mutant organoids. *Rpgrip1l* is among the top deregulated genes in the DESeq2 analysis we performed in the course of our RNAseq experiment. A table of such genes, including *Rpgrip1l*, is now provided as Supplementary Figure 1b.

We also performed WB analyses for RPGRIP1L in hiPSCs with the mouse-specific and human-specific RPGRIP1L ABs. The mouse-specific Rpgrip1l AB did not give a signal at the correct size on human samples [see below: blot 1) mouse specific Rpgrip1l AB]. The human-specific RPGRIP1L AB worked in some rare cases, in which we could detect a faint signal at the correct height in WT HEK (used as control; see Wiegering et al., 2018) and WT hiPSC samples. This signal was not detectable in RPGRIP1L KO HEK and KO hiPSC samples [see below: blot 2) human specific RPGRIP1L antibody]. However, given the faint signal and the high number of more intense non-specific bands, we decided to not include this WB to the manuscript.

For hiPSC clones, immunofluorescence analysis and sequencing data was provided for each clone (Figure 4D now Figure 4g; Figure S2C now Figure S2c; and Figure S3C now Figure S4d). We agree that it was not always clear how many clones, organoids or experiments were analysed for each figure. We apologise for this and have carefully revised the manuscript by clearly indicating the number of clones and experiments in each figure legend and by describing the measurements in greater detail in the Materials and methods section - Quantification of fluorescence staining and statistical analyses. In general, at least 10 organoids per clone from at least 3 independent experiments were analysed for all stainings and quantifications. In the case of cilia measurements, at least 100 cilia per clone were used for quantification.

1) mouse specific Rpgrip1l AB:

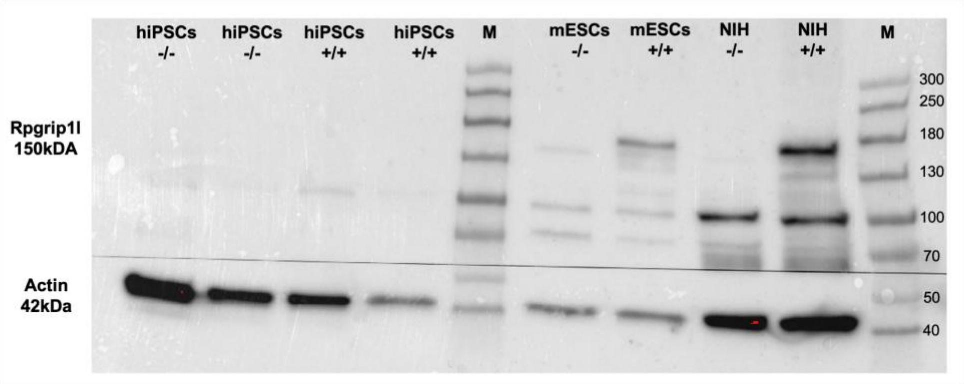

2) human specific RPGRIP1L antibody:

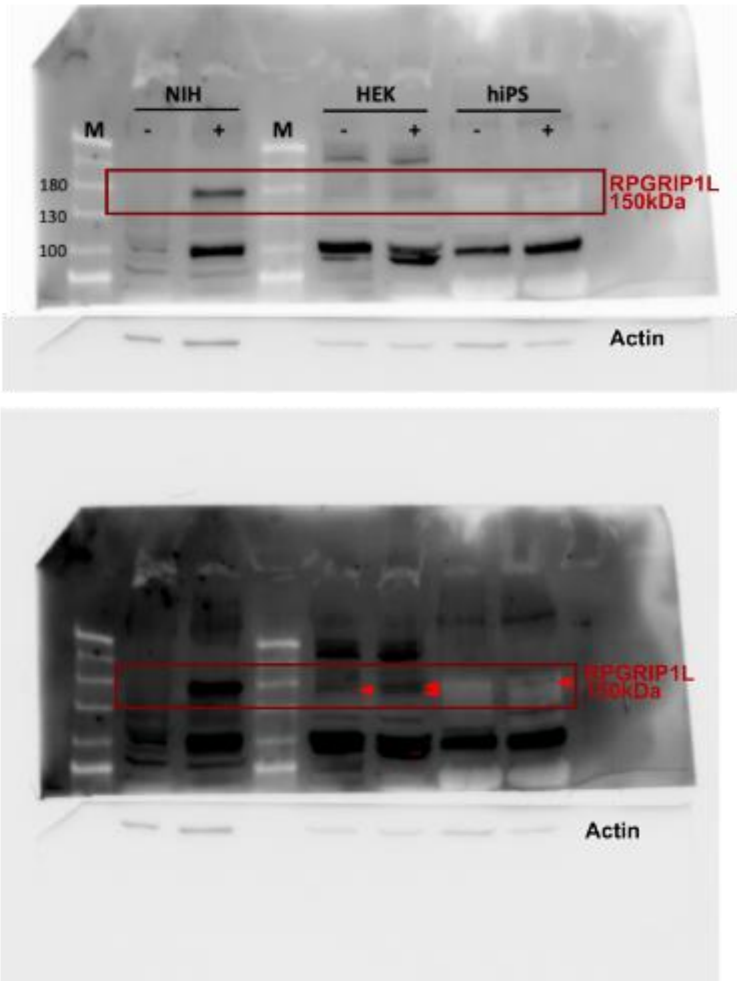

2. It´s not clear why the authors spent the effort and time in generating several RPGRIP1L mutant clones (theoretically KO as they claim to be introducing "premature STOP" codons in the sequence) from 2 different hiPSC lines (Fig S2) and yet they don´t use these lines at all in their experiments (only to perform RNAseq), and instead use a single KO clone generated by a different genome editing approach. Some key experiments should be also repeated in those clones, at least in one per line.

We agree that the explanation of which cell lines/clones were used for each experiment and why they were used was not clearly given in the first version of our manuscript. We have carefully restructured the text and figure legends to clearly state how many clones were used for each experiment and why we used different KO clones for RPGRIP1L (p.7 lines 1ff; p.9 lines 28ff; p.10 lines 6ff; p.18 lines 28ff). We hope it is now clear that each result presented in the main figures is based on the analysis of a total of 4 WT (now specified as n= 4) and 4 *RPGRIP1L KO* clones (n= 4) generated by *InDel* mutations. 2 WT and 2 KO clones were generated in the PCIi033-A hiPSC line, and the 2 others WT and KO clones in the UCSFi001-A hiPSC line. We chose to use 2 different cell lines and 2 clones for each genotype per cell line as controls of the effect of the genetic background on the phenotype. The small standard deviations show the low variability between individual clones within genotypes and between the two cell lines.

3. The claim that the altered rostro-caudal neural specification in the human RPGRIP1L KO spinal organoids is rooted on the lack of cilia in the NMPs is not really supported by causal, functional data but is rather a correlative finding, especially since it´s only shown in 1 hiPSC line (and 1 clone) and only when this TZ protein is missing but not when another TZ protein (TMEM67) is lacking.

At least rescue experiments by overexpressing RPGRIP1L at the NMP stage and measuring whether that rescues the abnormal rostro-caudal patterning should be performed.

Also, how do the authors explain that the lack of these two TZ proteins, RPGRIP1L and TMEM67, while having similar functions, as the authors indicate, have such drastically different consequences in NMPs?, meaning, "strong reduction in the cilia number" in the RPGRIP1L KO (Fig. 6H-J – for which quantifications must also be provided) vs no difference in the number of cilia in the TMEM67 KO (quantifications must also be provided).

We agree with the reviewer's comment on the missing cilia measurements and added the quantifications of cilia length and density to the revised version of our manuscript (Figure 6, Figure S8 and Figure S9).

To us, the differences in ciliary phenotype, NMP specification and AP patterning between RPGRIP1L and TMEM67 deficient organoids, are highly interesting as they will allow further analyses to understand cell type specific requirements for distinct ciliary proteins and their contribution to TZ stability and ciliary gating. We discuss this exciting perspective, as well as known differences between RPGRIP1L and TMEM67 functions, in the revised manuscript (p.4 lines 6 ff and p.15 lines 12 ff).

We further thank the reviewer for the very interesting proposal, to re-express RPGRIP1L in axial progenitors to precisely identify the stage and cell type whose perturbation leads to the AP patterning defect. However, we think that such an experiment would be very challenging (RPGRIP1L is a large protein of 1315 aminoacids, difficult to express in sufficient amounts) and too time consuming to be compatible with this manuscript revision. Moreover, our new experiments provide information on the stage of progenitor differentiation at which the phenotype appears: we have carefully reexamined and quantified the phenotype at early stages of spinal differentiation by performing labeling for CDX2 and co-labelling for SOX2/BRA at day 2 and day 4. We find that the number of CDX2-positive axial progenitors is significantly reduced as soon as day 2 (Figure 6 of the revised version). Such an early phenotype is consistent with a defect in WNT signaling, known to induce posterior neural identity via the activation of *CDX* genes (Gouti et al 2014, ref. 85 in the manuscript). Moreover, we have now quantified the ciliary phenotype at day 2 and day 4 and confirmed a drastic loss of cilia in *RPGRIP1L* mutant cells at both stages. We have modified our discussion (p.15 lines 20ff and pp.16 lines 4ff) to take these new results into account.

Indeed, we correlate the loss of cilia in RPGRIP1L deficient progenitors to a reduced CDX expression at the axial progenitors stage and to an altered specification of the NMP-like (SOX2+/BRA+) fate, resulting in a defective antero-posterior patterning. We show this correlation in all RPGRIP1L-deficient clones of both cell lines, in total 5 RPGRIP1L-deficient clones (Figure 5 and Figure 6 for RPGRIP1L *InDel* mutants; Figure S4 for the RPGRIP1L full deletion clone). The fact that early TMEM67-deficient progenitors have cilia, adopt the NMP-like axial fate and do not show antero-

posterior patterning defect further supports the idea of a correlation between cilia loss and defects in setting up HOX-mediated AP fate in axial progenitors.

4. Regarding the species-specific differences of TZ proteins in cilia formation and SHH signaling, despite the mouse organoids have a deficiency of cilia and SHH signaling, the authors claim that the antero-posterior patterning defect upon RPGRIP1L loss is specific to human and not present in mouse organoids. However, to support that statement measurements in mouse organoids of the expression levels and abundance (number of cells) of hindbrain vs spinal markers should be shown, besides the Hox genes shown in Fig S9H-J, which by the way, do not show the most rostral Hox (1-6). The authors claim that the altered rostro-caudal specification is found in human RPGRIP1L KO organoids but not in mouse, and that it is caused by the lack of cilia specifically and only at the NMP stage. Whether mouse KO NMPs have cilia or not should therefore be shown to clarify whether cilia in NMPs contributes to specify the RC identity in spinal cord development, across species, or whether these TZ proteins have completely different, and unknown, roles in cell specification in mouse and human.
If the authors have access to the ms ESCs, it would be interesting to show whether spinal organoids derived from Tmem67-/- show the same phenotype as the Rpgrip1l-/- ones (no rostro-caudal defects, in principle, but no ventralization). This would help to understand whether it's a single cilia TZ protein or the cilia itself what determines the RC and DV neural fate in the mouse spinal cord development.

To support our statement that mouse *Rpgrip1l KO*, in contrast to human organoids, do not undergo a spinal to cranial shift in antero-posterior identity, we now provide quantifications of Cdx2 immunostainings performed on WT and mutant organoid sections at day 3, from 3 independent experiments (Supplementary Figure 10f). We show that an average of 90% of nuclei in mouse day 3 organoids are positive for Cdx2, characterizing an axial trunk identity, and that this percentage is not significantly different between control and *Rpgrip1l KO* organoids, in sharp contrast with the human phenotype. We also performed immunostainings of WT and mutant organoids sections for the hindbrain marker Phox2b, and did not observe any expression of this marker in both genotypes. The quantification of Phox2b positive nuclei is presented below as violin plots where median and quartiles are indicated by dotted lines. We also provide for the reviewer a graph showing the temporal expression profile of the hindbrain marker *egr2* obtained in our bulk RNAseq, where it is obvious that in contrast to the human data (Supplementary Figure 7g), this gene is very lowly expressed in mouse organoids and that its expression doesn't differ between WT and KO organoids. As asked by the reviewer, we now provide a heatmap for the *Hox* gene clusters *HoxA* and *HoxC* depicting the log2(tpm+1) of all *Hox* genes expressed from these clusters, even the most rostral ones (Supplementary Figure 10m, n). These heatmaps clearly show that both control and mutant mouse organoids express high levels of *Hox6* and *Hox8* genes, characteristic of a brachial/anterior thoracic identity. We also validated these results by performing qPCR on 4 independent experiments, different from the RNAseq. These data are depicted in Supplementary Figure 10j-l. We believe that these results allow us to assert that murine organoids deficient for Rpgrip1l do not undergo spinal to cranial shifts of identity.

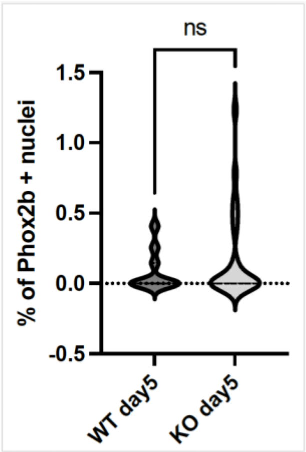

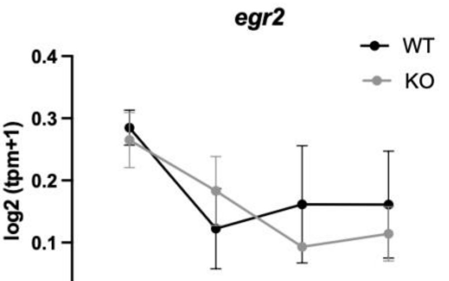

We agree with the reviewer that data on cilia at the NMP stage in mouse organoids were lacking. We performed immunostaining for the axonemal marker Ift81 on sections of WT and *Rpgrip1l KO* organoids at day 3 and found that comparably to what was observed in spinal organoids at later stages, cilia mutated for *Rpgrip1l* were blunted as evidenced with the measure of Ift81 length, which reduced from 1,4 µm in control organoids to 0,7 µm in mutant organoids. These

results were added to the Supplementary Figure 10, as Figures S10o and S10p in the revised manuscript. We think that indeed Rpgrip1l requirement for ciliary stability/construction might differ between human and mouse axial progenitors, explaining the differences in ciliary phenotypes between human mutant organoids, where the ciliary density is altered, and mouse, where cilia are present but are blunted.
We agree with the reviewer that testing the phenotype of mutant organoids derived from *Tmem67* KO mESCs would be interesting, unfortunately we do not have access to these cells. More generally, a detailed analysis of the function of cilia and different TZ proteins in early axial progenitors, although very interesting, is not central to the message of this paper and might be the subject of a future study.

5. It's somehow surprising that the authors have used quite different protocols for their mouse and human organoids. While for mouse, they go straight for WNT activation simultaneously to the caudalizing and ventralizing signals (RA+SAG), for human, neural induction with SB+LDN is the first step, together with WNT activation and prior to the RA+SAG. As the authors know, time, duration, concentration and combination of patterning molecules is key to guide the differentiation of PSCs into different cell fates. The authors should provide an explanation for such protocol difference.

Also, to generate NMPs from PSCs, for most published protocols a 2-4day pulse of CHIR+FGF2 is needed prior to neural induction (https://doi.org/10.1016/j.xcrm.2024.101659, https://doi.org/10.1016/j.stem.2019.12.007; https://doi.org/10.1242/dev.119768; https://doi.org/10.1371/journal.pbio.1001937 and others), which the authors do not follow. Therefore, to prove that their mouse and human spinal organoids are indeed generated from EBs composed by NMPs, these should, at least, be stained for SOX2 and TBXT at early stages of their development (before day 4 according to their protocols) and the percentage of double positive cells must be shown. In page 7 they claim "Within four days, WT and RPGRIP1L-deficient cells adopted a caudal NMP-like axial fate" but the data supporting that claim is not shown. This data should come early in the manuscript, not in Fig 6.

We indeed used different protocols and are aware that it could alter the efficiency of NMP formation or their level of caudalization. However we chose to use protocols suited to maximise the efficiency of motoneuron and ventral spinal differentiation for each species, relying on already published studies (Li et al., *Nat Biotechnol* 2005, https://doi-org.insb.bib.cnrs.fr/10.1038/nbt1063; Wichterle et al., *Cell* 2002, https://doi.org/10.1016/S0092-8674(02)00835-8; Thiry et al., *J. Neuroscience* 2020, https://doi.org/10.1016/j.neuroscience.2020.04.041; Mouilleau et al., *Development* 2021, https://doi-org.insb.bib.cnrs.fr/10.1242/dev.194514). Here it is demonstrated that FGF addition is not required to generate NMPs prior to neural induction, since FGF is produced endogenously by 3D axial progenitors as in embryos. Indeed, most protocols recently developed to produce 3D gastruloids induce NMPs by addition of the Wnt activator CHIR (Moris et al., *Nature* 2020, https://doi-org.insb.bib.cnrs.fr/10.1038/s41586-020-2383-9; Turner et al., *Development* 2017, https://doi.org/10.1242/dev.150391).

We firmly believe that we convincingly demonstrate high efficiency in obtaining spinal organoids from mouse and human PSCs that are sufficiently comparable to draw conclusions about the species-specific differences that we emphasise in our study. However, to answer the reviewer's comment and prove that our spinal organoids are indeed generated from NMPs as it was published before (human: Maury et al., *Nat Biotechnol.* 2015, https://doi.org/10.1038/nbt.3049 and Mouilleau et al., Development 2021, https://doi.org/10.1242/dev.194514; mouse: Duval et al., *Development* 2019, https://doi.org/10.1242/dev.175430), we performed combined antibody stainings for SOX2 and BRACHYURY in early mouse and human spinal organoids, and included these data in the revised version of our manuscript (Supplementary Figures 8c-h, 9e and 10e, h, i). We show that NMP-like cells co-expressing SOX2 and BRACHYURY are present in both human and mouse organoids models generated in our study. Moreover, we now present the temporal expression profiles of *Brachyury* obtained from the bulk RNAseq analysis of mouse organoids which clearly demonstrate that mouse organoids undergo a transient pic of *Brachyury* expression between day 2 and 3, suggesting that NMP like cells are indeed induced (Supplementary Figure 10g). Finally, to address the reviewer's concerns about the differences in protocols used for both species, we applied an alternative protocol of spinal differentiation to mESCs. Here, similar to the human protocol, cells were neuralized with SB+LDN prior to addition of CHIR to activate Wnt at day 2. RA and SAG were then added to the medium from day 3 onwards. 2 independent differentiation experiments were carried out, from which we performed immunostainings to test for the presence of Cdx2 positive and Brachyury/Sox2 double positive axial progenitors. Altogether, our results show that even when applying a differentiation protocol closer to the human one, mouse mutant organoids do not present alterations in the induction of axial fate. We also found that *Cdx2* induction was less efficient in this protocol, with significantly less cells stained for Cdx2 in organoids (see graph below (****$P < 0.0001$, unpaired t tests with Welch's correction).

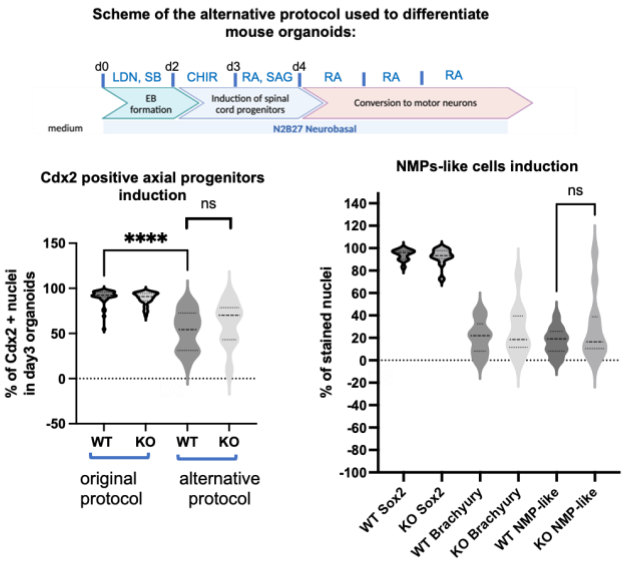

6. The authors should clearly state the number of independent experiments performed for each figure panel. For example, quantifications for Fig. 1E-G are supposed to be "n sections analyzed ≥8, from ≥2 experimental replicates". As the authors know, organoids are very heterogeneous both across intra- and inter-experiments. At least 3 different sections from 3 different organoids (n=3) from at least 3 independent experiments (N=3) should be always analyzed to guarantee the robustness and reliability of the data. Regading the mouse RNAseq, was it performed in 3 EBs from the same experiment (n=3) or from 3 independent experiment with multiple EBs each pooled together (N=3)? 3 independent experiments is always the minimum to be able to reach any conclusion. Also, each of those experimental points should be shown separately rather than pooled to show the heterogeneity/robustness of the results.

We thank the reviewer for drawing our attention on this point, since it was indeed not clearly stated how many organoids and clones were used in each experiment. We analysed sections from at least 10 organoids per clone in at least three independent experiments per condition. We carefully revised the figure legends as well as the material and methods section and adopted the reviewers suggestion by using the following nomenclature:
n = number of different clones
N = number of experiments
As now stated in the material and method section (p. 22 lines11 ff), for the mouse RNAseq analysis, pooled organoid samples from Wt and *Rpgrip1l* KO were collected at each time of the differentiation in the course of 3 independent experiments (N=3). The heterogeneity or robustness of our experiments is indicated by small standard errors in our experiments, either between individual clones of one genotype, between the two cell lines or between different experiments. As our results are furthermore corroborated by several complementary methods (RNAseq, IF, qPCR), we consider them to be reliable.

To provide more details on the variation between hiPSC clones, we attached below 4 graphs that show the quantification of immunofluorescence data for OLIG2 (related to Figure S3b in the revised version), ISLET1 (related to Figure S3f in the revised version), BRACHYURY (related to Figure S8h in the revised version) and HOXA7 (related to Figure 5g in the revised version) for each analysed clone at specific differentiation stages.

Quantification of OLIG2, ISLET1, BRACHYURY and HOXA7 in WT and RPGRIP1L KO (*InDel*) clones:

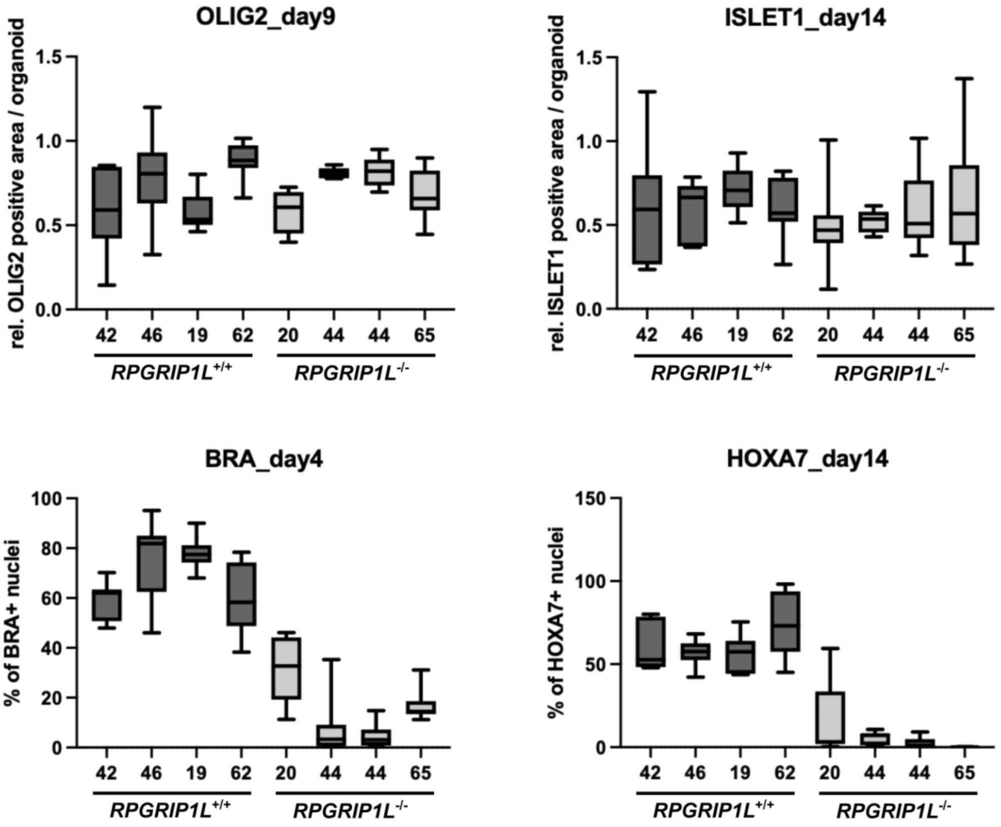

Furthermore, we attached below several heatmaps showing the independent expression values (log2(tpm+1)) of each clone and time point generated from our RNAseq analysis.

Expression of progenitor markers along the D/V axis in WT and *RPGRIP1L KO* clones (related to Figure 3c of the revised manuscript):

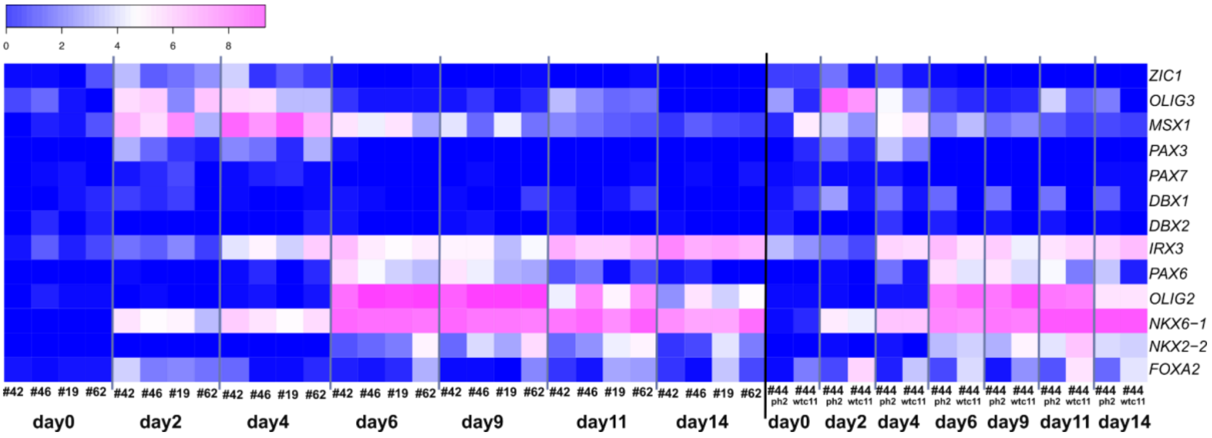

Heatmap showing the expression of *HOXA* and *HOXC* genes in WT and *RPGRIP1L KO* clones (related to Figure 5a, d of the revised manuscript):

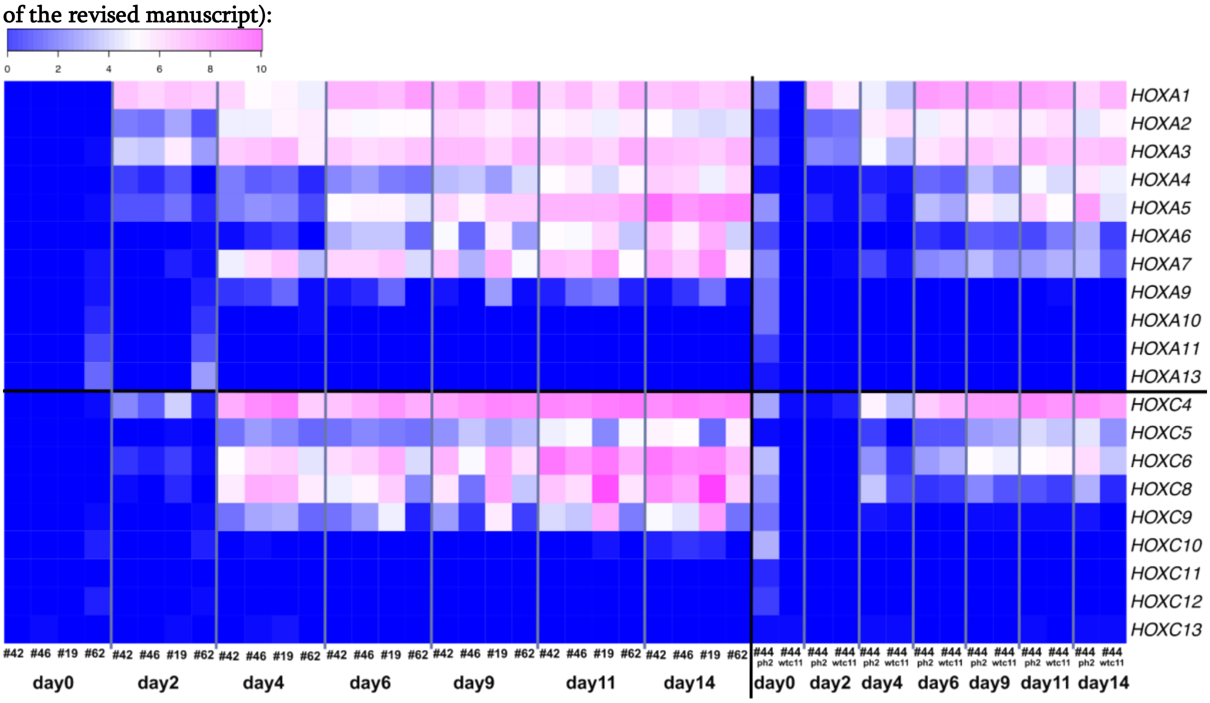

Due to space limitations and also for reasons of comprehensibility, we prefer to stick to graphs and heatmaps presenting mean values in the manuscript.

7. The quantifications of immunostained spinal organoid sections are per "positive area". Instead, the number of cells positive for whatever marker over the total number of cells should be quantified (at least definitely for the nuclear markers measured -which is most of them-) to provide accurate quantifications.

We validated the method of quantifying the positive stained areas by re-analysing images via automated segmentation of nuclei using Cellpose (python environment), combined with manual corrections when necessary (Material and Methods section: Quantification of fluorescent staining and statistical analyses). The results were consistent (see the graph attached below), so that we retained the method of measuring positive areas for previously analysed experiments. For new experiments or experiments in which we increased the number of analysed organoids, we implemented the new method of nuclear segmentation with cellpose.

Graphs showing the quantification of immunofluorescence stainings of HOXA5 and HOXC8 (related to Figure 5c of the original manuscript and Figure 5i, h of the revised manuscript):

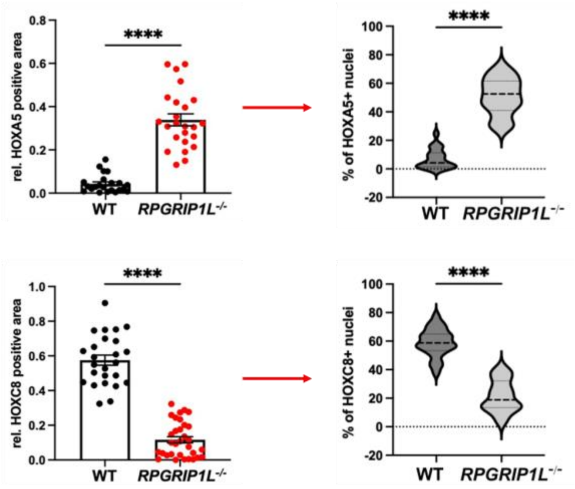

What exactly each graph shows is sometimes difficult to grasp. E.g. in Fig 2E (S2C, S3C and others), does "mean %of ARl13b+ cilia" mean the percentage of cells in the organoid with cilia? Or Fig S4F (containing important data to support that the RPGRIP1L KO organoids do not contain an enhanced % of pMNs and yet it's a supplemental figure coming from an unclear N and with no statistics). This graph should show the # cells+ / total #cells because if the organoids have different size or different cell densities, these results do not mean anything.

We apologize for the incoherent labeling of our graphs and the unclear statements about the methods of measurement. We revised the figure legends carefully and homogenized the methods of quantification and illustration.
Regarding Figure S4F (Figure S3g in the revised version of our manuscript), we fully agree with the reviewer and presented the data as "% of cells / organoid slice" in the revised version of our manuscript to take into account the different sizes and cell densities of the organoids. As indicated in the figure legend combined with information in the material and methods section, 4 WT and 4 KO clones were analysed at each stage, taking into account at least 10 different organoids per clone. Chi-square tests did not reveal any significant difference here.

Also, in relationship with the NKX2.2 results, the authors say "Here, expression of NKX2.2 plays a primary role in ventral neuronal patterning and requires high SHH signaling…", which implies that the specification of NKX2.2- neural types in the ventral spinal cord do not rely on SHH signaling. It seems that the authors used this argument to link this section with the next, on SHH, but does not seem correct.

We corrected this text passage in the revised version of our manuscript (p- 8 line 16-18).

8. most of the mRNA expression levels shown in the multiple graphs come solely from the RNAseq performed. qPCR from additional independent experiments must be performed as validation of that single RNAseq dataset.

Even though most of the sequencing data was validated by immunofluorescence analyses, we completed the validation of the bulk RNAseq data by qPCR from independent experiments. Validation for selected genes are now shown in Figure 1k-l; Figure 2a,b and Supplementary Figure S3e, h, i; S7b-f and S10j-l. Furthermore, we expanded qPCR analyses to full deletion clones of RPGRIP1L (Figure S4g, h, i, n, o) and TMEM67 (Figure S5h-j and Figure S9f, h).

9. To ease the readability and put all results into context, the discussion should not contain separate sections.

We revised the discussion carefully and removed separate sections.

Minor comments:

- The authors should acknowledge that a few studies describing the role of ciliary proteins on the establishment of DV

patterning via SHH regulation using brain or spinal organoids have been published (e.g. doi: 10.1016/j.celrep.2022.110811, doi: 10.1371/journal.pone.0301670)

We agree with the reviewer and included the proposed as well as additional references in the revised version of our manuscript (p.3 lines 30ff, p.5 line 12 and p.6 line 31).

- Although of relative importance depending on the intention of the authors, statements like "we could not detect any difference between…" should be replaced by "we did not detect…", because "could not" implies the purpose of finding something specific and that implies bias.

We revised the wording in several statements according to the reviewer's suggestion.

- In page 5, lines 29-30, the statement "Strikingly, Rpgrip1l-deficient progenitors failed to commit to ventral spinal identities" does not seem appropriate, since, unless I misunderstood it, that result was precisely what was expected.

The reviewer is completely right. We expected this result and therefore we have corrected this sentence in the revised version of our manuscript.

- Fig S1D-E need further explanation because currently they aren´t very informative (the genes in D aren´t readable). What does the color indicate? And how high in the GSEA ranking does SHH signaling and spinal cord patterning appear?

We made changes to Supplementary Figure S1 to ameliorate the clarity of the information provided. In Figure S1d,e, the color key was added, and the list of genes magnified. Information concerning the GSEA is now provided in the figure legend. We also included a table of selected differentially expressed genes between mouse WT and mutant organoids at day 4, including the Log2FC and p-value obtained from analysing our bulk RNAseq data using DEseq2 (Supplementary Figure S1b).

- In page 7, where is the data supporting the claim "Importantly, for each genotype, Wtc11 and 1 Ph2 hiPSC lines behaved the same in terms of differentiation efficiency, cilia phenotype and gene expression profiles"?

We again apologize that we did not clearly state how many clones, organoids and experiments were used for each analysis. As we described in our response to major criticism #6, 2 WT and 2 KO clones of each hiPSC line (WT: n=4 and KO: n=4) were used for each analysis in the main figures and both cell lines were used in the bulk RNA seq experiment. The small standard deviations on our graphs (for example in Figure 3b of the revised manuscript) show the low variability between individual clones within a line and between the two cell lines. Furthermore, PCA results show the close clustering of both hiPSC lines at each analysed time point of the differentiation (Supplementary Figure 6b of the revised manuscript).

- Some figure panels are not referred to in order (e.g. Fig 3E before 3D, 5G before 5F, S4B is cited before S3D-F; and S4D-E don't seem to be cited at all), which makes it confusing to go over the figures.

We re-organised the figure panels and referred to each figure panel in the revised version of our manuscript.

- In some figures (e.g. Fig 3 and S4A-C) only a small fraction of the organoids is shown. The entire organoids should be shown instead.

We replaced figure panels to show entire organoids in Supplementary Figures S3a, b, f; S4l, m, p and S9e, g, i of the revised version of our manuscript.

- In Fig. 4E, organoid images with only DAPI should be shown to indicate where the ventricle-like structures are located. In addition, no clearARL13B signal is detected in the outside of the organoids shown, so maybe that statement needs to be rephrased in page 9?

We think that the Arl13b staining makes it easier to see the ventrice-like structures, since cilia are present at ventricular borders. We rephrased the statement (p.9 lines 6-8 in the revised version).

- In figure 5E, the statement that the rostral MN marker PHOX2B is expressed in the RPGRIP1L KO organoids but not in WT is incorrect. PHOX2B is also expressed in WT but to a lesser extent. As mentioned before, the RNAseq data must be validated by qPCR in multiple independent experiments.

We validated the RNAseq data by qPCR analyses of *PHOX2B* (Supplementary Figure S7f) and revised the statement carefully (from p.11 line 35 to p.12 line 2).

- Fig S7G is referred to in the text as data on axial progenitors when it seems to refer to spinal organoids. This should be corrected.

We thank the reviewer for revealing this mistake and revised the text accordingly (p.12 line 15f) (Supplementary Figure 7k in the revised version).

- The data shown in Fig 6C (RNAseq) and 6E (immunostaining) does not fit. This should be explained.

We agree with the reviewer and wondered about this result, too. In fact, our statistics on immunofluorescence data were not strong enough. In the revised version of our manuscript, we included new CDX2 stainings and quantifications at day 2 of differentiation as well as increased the number of clones and experiments analysed at day 4 of differentiation. Thanks to this, we can now demonstrate the significant reduction of CDX2 in RPGRIP1L-deficient organoids at day 2 and day 4 of differentiation (Figure 6a-d).

- Line 23 in page 12, do the authors mean Hoxd10 instead of HoxPG10?

This sentence has been changed.

Reviewer #3 (Remarks to the Author):

Overview and noteworthy results

The study by Wiegering et al. highlights species-specific roles for the transition zone gene RPGRIP1L in spinal cord development, demonstrating that findings in mouse models may not fully capture the complexity of human disease mechanisms. While mouse Rpgrip1L mutants show severe defects in SHH signaling and motor neuron specification, human RPGRIP1L-deficient organoids retain normal SHH signaling and retain cilia, but display a distinct shift in anterior-posterior motor neuron identities. More specifically, human RPGRIP1L-deficient organoids exhibit a shift from thoracic to hindbrain motor neuron identities, marked by altered HOX gene expression, a novel role for RPGRIP1L not seen in mice. This divergence between species underscores the risk of over-reliance on mouse models for understanding human neurodevelopmental diseases. By using human stem cell-derived organoids, this work suggests that human cell lines can offer a more accurate representation of disease phenotypes and are likely vital to our understanding and treating complex diseases like Joubert Syndrome (which can be caused by defects in RPGRIP1L). The findings suggest that using human-specific models could reshape our approach to studying neurodevelopment and developing targeted therapies. I believe the findings in this paper are very interesting. These findings have broad implications not only for ciliopathy research but also for the study of other diseases where species-specific differences might play a critical role.

Will this work be of significance to the field?

It is widely known that ciliopathy phenotypes in mice are more severe than in humans. The best example I can think of is hydrocephalus, where ciliary defects in mice frequently result in hydrocephalus, but this phenotype is observed to a much lesser degree in humans. This example speaks to motile cilia and may be a product of different brain anatomies. In this paper, through a detailed examination of transition zone mutants, the authors provide compelling evidence of more subtle trafficking (and thus signaling) phenotype differences between mice and humans. I think this would be of high interest to the field and will expand upon our understanding of human ciliopathies.

Does the work support the conclusions and claims?

Yes, the work strongly supports the conclusions and claims made. The data is exceptionally thorough and well-presented. As a developmental biologist, I feel like most studies focus on a snapshot of developmental time. What I found striking about this paper was the detailed transcriptional analysis performed throughout the differentiation process. This time-course transcriptomic analysis provided beautiful insights into how RPGRIP1L deficiency impacts the gene expression landscape, highlighting specific changes in anterior-posterior patterning, including shifts in HOX gene expression and the misspecification of motor neuron identities. The dynamic profiling of gene expression is especially compelling and directly links cilia-related defects to alterations in spinal progenitor differentiation, solidifying the authors' claims. Most surprising to me was the novel finding that human RPGRIP1L-deficient organoids do not exhibit the same SHH signaling defects as mouse models, but instead show unique anterior-posterior patterning shifts. Admittedly, I was skeptical at first, as negative data (i.e. a loss of a defect) is hard to support and can easily be explained by an incomplete knockout or knockdown. However, the analysis of multiple clones and the use of multiple approaches to ultimately create the RPGRIP1L-deficient organoids was compelling.

Is the methodology sound?

My area of expertise is spinal cord development and hedgehog signaling. The methods and analysis in this paper were superb. I am familiar with the cell culture techniques and the detail provided in the methods is more than sufficient to allow others to reproduce the experiments.

Final thoughts

This is a well-written paper and seemingly the exciting beginning of many more studies. I was particularly interested in how much further these species-specific differences exist. For example, perhaps the composition of the mouse and human transition zone are different. In addition, as alluded to in the discussion, it will be very interesting to identify the origin of the anterior-posterior patterning changes – maybe it is due to alterations in Wnt signaling, which would be fascinating

because the role of primary cilia in Wnt signaling is still quite a controversial. In conclusion, the paper seems very complete and I support its publication. While perhaps beyond the scope of this study, the only thing that would ask for that I think would complete this study is an analysis of Joubert Syndrome iPSCs, to see if the phenotypes compare to the RPGRIP1L-deficient cells generated in this study.

We thank the reviewer for this interesting opening to pathology; investigating the anteroposterior patterning and, more generally, the phenotype of spinal organoids from JS patients with mutations with RPGRIP1L is indeed in our short-term projects. However, we think it is beyond the scope of this manuscript. We have indicated this perspective in the last paragraph of the discussion (p.17 lines 3ff).

Small point, I was also very puzzled by the result in Figure 3D -- it seems like an outlier on many levels (transient effect and inconsistent with all the other data in the figure).

We have revised the text for Figure 3D (Figure 3h in the revised version of the manuscript) to discuss possible explanations for this result. We think that the increase in NKX2.2 can be most likely explained by the increase in visceral hindbrain MNs expressing NKX2.2 but not OLIG2 (p.8 lines 10-16 and p.9 lines 1f).

Reviewer #2 (Remarks to the Author):

As I wrote in my original review, I found this story interesting, both from the point of view of the conceptual knowledge gained on the role of the cilia in spinal cord NP specification and AP patterning and from the inter-species differences angle. I had, however, lots of comments and concerns back then, and although the authors have clearly made an effort to address them, some of them remained unanswered and their responses to others have raised additional doubts. I firmly believe that fully addressing them would significantly improve the reliability of the data and the robustness of the claims. They are indicated below according to their numbering in the rebuttal letter.

We thank the reviewer for his/her careful review of our manuscript and insightful suggestions. We have now modified the manuscript accordingly, and hope we have addressed all the reviewer's unanswered questions.

1. Doesn't matter if these mouse cells have been published before (even by this same group), it is a must to show proof that the KO cells one works with indeed lack the protein of interest. We all know that immunostaining quantifications can be totally random (e.g. is the acquisition parameters aren't identical across samples, and especially if the sampling isn't blind), which is much harder to alter in a WB. The WB does not have to replace the immunostaining, both panels should be shown in the figure.

As requested by Reviewer 2, a Western Blot showing the complete absence of the Rpgrip1l protein in KO mouse ES cells was added in Fig. 2f. The original WB was also uploaded as a "raw data file".

For the human cell lines, none of the tested RPGRIP1L antibodies worked in WB, including a new home-made antibody raised against the human protein. For IF data, to remove the reviewer's doubts about our quantification procedure, we provide raw confocal image data (N=1 for each *InDel* clone) which show that the same imaging parameters were used for WT and mutant clones.
https://dropsu.sorbonne-universite.fr/s/X2zzJg3tQkGJwZ5

Where are the "sequencing data for each clone" provided? For the above reason, if WB used can't be shown because the AB works for immunostaining but not WB, then at the very least the sequencing data of the targeted clones must be shown. Figure S2b is simply a list of mutations, which, as the authors should understand, is not a proof of anything, especially when a paper is being revised for potential publication and when the whole study is based on these edited cells. The Sanger sequencing chromatogram aligned to a reference sequence must be shown. Even RNAseq data showing mRNA expression of RPGRIP1L mRNA in the mutated and KO cells could be shown, like for the mouse cells in Fig. S1b, although, that's just mRNA and not protein, which is what this study cares about.

As requested by the reviewer, we now provide the sequencing chromatograms for each clone used in this study in Supplementary Fig. 2b, and new Supplementary Fig. 11 and 12 of the revised manuscript. We also provide raw sequencing data to the reviewer as .ab1 files that can be opened in SnapGene viewer or similar softwares.
https://dropsu.sorbonne-universite.fr/s/X2zzJg3tQkGJwZ5

Also, why the "human specific RPGRIP1L antibody" (3rd blot) detects a band of the right mw in the NIH3T3 cells (mouse fibroblasts)? Either that AB is not human-specific or that band is not RPGRIP1L and therefore the WB shown for the mouse cells unfortunately can't be trusted either.

We agree that our previous response to the reviewer was not clear, as we wrongly stated that this antibody was human-specific. We meant to explain that it was raised against the human RPGRIP1L protein (see Supplementary Table S2). Indeed, it does recognize the mouse protein. The reason why this antibody does not work in WB for human samples is unclear to us.

2. I'm more confused than before because at least the original text said which lines were used for each experiment (even if this was wrongly missing in the figure legends), but in the revised version this information in the text is crossed out and is still missing in the legends, so there is no way to know which data comes from which cell line. For example, all fig. 3 seems to refer to one single WT line (and clone) and one single KO line (and clone), unknown which one, and yet all throughout the figure legend it's written "WT: n=4; KO: n=4", what does that mean?? The exact same happens to every single figure. Or does it mean that the results from all the WT and the KO clones, even from different lines, are combined in the same graphic?, but what is the logic of that then?, why claiming to make multiple clones from multiple lines if all the results will be cluttered into the same plot? This sounds very strange. Which cell line has been used in each experiment is obviously basic information that should be at least in the legend, if not in the main text too.

We are sorry reviewer 2 found it difficult to understand what cells were used in the experiments shown despite the consequent changes we made to ameliorate the overall clarity of the paper. As already explained in the results section page 7 line 1 to 7 (marked-up manuscript version), as well as in the material and methods section page 18 line 29 to page 19 line 11 (marked-up manuscript version), the Crispr/Cas9 genome editing was performed in 2 different hiPS cell lines. For each of these cell lines, 2 control and 2 KO clones were used. WT and KO clones from the 2 different cell lines showed a similar molecular signature (Supplementary Figure 6) and behaved similarly (as we have already demonstrated in our previous point to point letter by providing IF and RNAseq data for single clones), showing the robustness of *RPGRIP1L KO* phenotype. Therefore, we considered the number of clones used for each genotype (n=4 WT and n=4 *RPGRIP1L KO*) independent of the cell line. The number of experimental replicates performed for each clone is indicated as “N”.

In agreement with reviewer 2’s request, the details of which lines were used in each experiment were indicated in each figure legend of the revised manuscript.

The justification of “The small standard deviations show the low variability between individual clones within genotypes and between the two cell lines” does not seem accurate as, in many of the bar graphs present in the revised version (some of which still don’t display the independent experimental values) the error bars are several orders of magnitude (e.g. Fig. S4i, n, S7b-e, S9f, etc), and some of the line-based graphs still don’t have stats (e.g. Fig. 3d-e, 5b-f, k-l, 6e, etc).

Indeed, results shown in Fig S4, S7 and S9 refer to the RPGRIP1L full deletion and to the TMEM67 full deletion clones, for which we only have one clone of each genotype (n=1).

According to the reviewers comment, we added statistical information to several quantifications (Fig. 3g, Suppl. Fig. 3 e, g, h, i, Suppl. Fig. 4 g, h, i, n, o, Suppl. Fig. 5 h, i, j, Suppl. Fig. 7 b-f, Suppl. Fig. 9 f, h) and carefully revised the figure legends to provide full information about number of clones, number of experiments, and statistical tests (see also Material and Method section Quantification of fluorescent staining and statistical analyses).

Statistical analyses were performed on line-based graphs as indicated above, except when normalized count [log2(tpm+1)] values from our RNAseq data were plotted against time. This representation was shown to illustrate variations in gene expressions during the course of differentiation. However, for statistical analysis of differentially expressed genes, we used DESeq2. As this method normalizes the reads on the sequencing depths of each sample, it is a more accurate way of across-sample comparisons in RNAseq than [log2(tpm+1)]. We provide DESeq2 data (FoldChanges and adjusted p-values) for each analyzed time point in a new Supplementary Table S3 and DESeq2 gene list for each time point to the reviewer.
(txt files that can be opened with Excel: https://dropsu.sorbonne-universite.fr/s/X2zzJg3tQkGJwZ5)

3. I find this response insufficient. Using a lentivirus to overexpress a 150 kDa protein is more than possible, and the time constrains aren't well supported either as the organoids are not analyzed past day 14. If the suggested rescue experiment is not done, 1) that claim can't be made but the idea only proposed, and 2) the new experiment the authors referred to must be solid, although still correlative. Unfortunately, I find these new results very worrisome. In the original version, Fig. 6E showed NO difference in the #CDX2+ cells between RPGRIP1L KO and WT day 4 organoids, however, in the new Fig 6d data the statistical significance has 3 stars?

Much later, and only due to a different (and theoretically minor) comment, the authors somehow justify this surprising change by saying “we included new CDX2 stainings and quantifications at day 2 of differentiation as well as increased the number of clones and experiments analysed at day 4 of differentiation” and by doing that the CDX2 results drastically changed. However, weren’t also in the original version all clones analyzed and merged together (strangely as commented above)?, so what more clones are added now? Unfortunately, this also sounds odd.

In line with the reviewer’s comment, since we do not provide the formal demonstration that a defect in NMP specification is causing the antero-posterior phenotype, we have mitigated our statement that altered rostro-caudal neural specification in the human RPGRIP1L KO spinal organoids is rooted on the lack of cilia in the NMPs (page 13 line 14f and line 32f, page 16 line 9).

Concerning the CDX2 expression data, we do not agree with Rev2's statement that the results drastically changed between the two versions of the manuscript. A reduction in CDX2 was already indicated in the first version of our manuscript (Figure 6c, e - figure panel 2 for RPGRIP1L KO), where we had clearly stated that this data was coming from n=2 WT and n=2 KO clones. To strengthen our data, in our first revision we provided new quantitative data on CDX2 protein expression, adding two clones and one experimental replicate. With this new experiment, the quantification of the data showed a significant reduction in the number of CDX2 positive axial progenitors in RPGRIP1L deficient organoids at both days 2 and 4. We provide the reviewer with a link to the two sets of raw data of CDX2 IF. https://dropsu.sorbonne-universite.fr/s/X2zzJg3tQkGJwZ5

The reduction is further supported by our RNAseq temporal profiles where a clear reduction in *CDX2* transcripts is observed in mutant organoids as soon as day 2 (Figure 6e, and new Supplementary Table S3). Finally, immunostainings and RNAseq experiments show a decreased proportion of BRA expressing progenitors from day 4 in RPGRIP1L mutant organoids (Supplementary Figure 8), reinforcing our conclusion on the decrease of NMPs upon RPGRIP1L deficiency.

To the comment: “we correlate the loss of cilia in RPGRIP1L deficient progenitors to a reduced CDX expression at the axial progenitors stage and to an altered specification of the NMP-like (SOX2+/BRA+) fate… We show this correlation in all RPGRIP1L-deficient clones of both cell lines, in total 5 RPGRIP1L- deficient clones (Figure 5 and Figure 6 for RPGRIP1L InDel mutants; Figure S4 for the RPGRIP1L full deletion clone) “. Fig 5 does not mention CDX2, SOX2/T or even day 2-4 organoids, and neither does S4.

We apologize to the reviewer for this incoherence between our previous response letter and the manuscript text and figures.
Indeed, in our manuscript, we discuss the observed phenotype and the correlation of RPGRIP1L loss and defective NMP specification in *InDel* RPGRIP1L mutants only (Figure 6 for cilia and NMPs, and Figure 5 for AP patterning) (from page 12 line 24 until page 13 line 13 in the marked-up manuscript version). We did not cite the full deletion RPGRIP1L clone here, as mentioned by the reviewer, data on cilia and NMPs are not provided. Nevertheless, we discuss the absence of phenotypes in TMEM67 mutants, which is in line with our hypothesis (Supplementary Fig. 8 and 9) (page 13 line 19-33 in the marked-up manuscript version).

5. The authors should still provide an explanation as to why using these patterning molecules in a different order for mouse and human. Saying that you follow published protocols to maximize MN specification isn't enough, as there endless different protocols out there making similar claims. What's the rationale the authors used?, do they know why they used those molecules in that order?
In addition, how can one "convincingly" claim that the human and mouse organoids are comparable in NMP composition when, for example, your day 4 human (WT) organoids are 100% SOX2+ and 100% BRACHYURY+ (e.g. Fig. S9e), however your mouse day 3 organoids show high SOX2 levels (# cells not quantified, DAPI not shown) but Brachyury is barely present? (Fig. S10i)? That simple comparison says that your NMP-made spinal organoids are not "sufficiently comparable". Maybe the mouse analysis should have been done at day 2 instead of at day 3 to have a really comparable human-mouse model.

Also, as the authors should know, NMPs are typically identified by the double expression of SOX2 and TBXT (BRACHYURY) (they actually say in the revised discussion "Later (at day 4), reduced numbers of BRACHYURY+/SOX2+ NMPs are found in RPGRIP1L mutants"). However, no quantification of double+ cells is shown for any of their human organoids, which is shown for their mouse organoids (Fig. S10h) and, strangely measures 0% double positive cells, which could indicate, among other things, that those mouse organoids at day 3 are already committed to neural lineage and therefore aren't NMPs anymore.

We quantified the number of BRA/SOX2 double positive cells in WT, *RPGRIP1L KO*, and *TMEM67 KO* human organoids and included the results in the revised version of the manuscript (Supplementary Fig. 8f, j and Supplementary Fig. 9e).

The protocols we used in this study are adapted from Duval et al., *Development* 2019 and Mouilleau et al., *Development* 2021. Both are 3D embryoid bodies-based differentiations which rely on an early pulse of WNT activation to induce axial caudal CDX2+ progenitors, and retinoic acid to prevent mesodermal differentiation and neuralize spinal progenitors. In Supplementary Figure 10m, we show that day 3 mouse organoids have an average of 20% Sox2; Brachyury double-positive cells, and range from 0 to 60%, which was probably misjudged by Reviewer 2 due to the graphical representation. We have modified the tick interval of the Y axis in our violin plot for better readability. Presence of NMPs was also confirmed by our RNAseq results highlighting the sharp and transient increase in *Brachyury*, *Cyp26a*, and *Cdx1-2-4* expression between days 2 and 3, as expected upon Wnt induction by CHIR (Supp Fig10 a-d, n). From these results, we conclude that at least part of the progenitors in mouse organoids transit through an NMP stage between day 2 and day 3.

However, as stressed by the reviewer, most progenitors already committed to the neural fate at day 3, probably due to the addition of RA at day 2. As mentioned by the reviewer, we can't exclude that the inter-species differences in NMPs phenotype between mouse and human are due to differences between the protocols used.
In agreement with the reviewer's requests, we removed our statements from page 14 line 3-5 and line 20-21 (marked-up manuscript version), and added a paragraph on page 16 line 17-30 (marked-up manuscript version) of the discussion section, where we comment on these possibilities and mitigate this inter-species difference.

7. And are those numbers now 5% or 0.05% (first black bar)? The legend in combination with the Y axis indicates the latter (wrongly, I guess).

In our first response to the reviewer, we presented the graphs of Figure 5c of the original manuscript and Figure 5i, h of the revised manuscript. In the graphs of the original manuscript, we plotted the relative HOXA5 and HOXC8 positive areas, as it was clearly indicated on the Y axis of the graphs (rel. HOXA5 positive area). In the revised version, we plotted the percentage of HOXA5+ or HOXC8+ nuclei. This was clearly indicated on the Y axis of the graphs (% of HOXA5+ nuclei). A relative amount of 0.05 is equal to 5 %.

Minor:
- The table included in Fig. S1b on "selected" DEG doesn't answer my question of "how high in the GSEA ranking does SHH signaling and spinal cord patterning appear?"

As stated in the Supp Fig1 legend, in the EGSEA performed on differentially expressed genes between WT and Rpgrip1l$^{-/-}$ organoids on day 4, spinal cord patterning ranked 1st for c5 GO Gene Sets (c), and SHH pathway ranked 1st for h Hallmark Signatures (d).
